# Sidekick: A Low-Cost Open-Source 3D-printed liquid dispensing robot

**DOI:** 10.1016/j.ohx.2022.e00319

**Published:** 2022-05-26

**Authors:** Rodolfo Keesey, Robert LeSuer, Joshua Schrier

**Affiliations:** a Department of Chemistry, Fordham University, 441 E. Fordham Road, The Bronx, NY 10458, USA; b Department of Chemistry and Biochemistry, SUNY Brockport, 350 New Campus Drive, Brockport, NY 14420, USA

**Keywords:** Laboratory Automation, 3D Printing, Chemical Experimentation

## Abstract

The Sidekick is a desktop liquid dispenser, compatible with standard SBS microplates and designed for accessible laboratory automation. It features an armature-based motion system and a fully 3D-printed chassis to reduce overall mechanical complexity and accommodate user modification. Liquid dispensing is achieved with four commercially available solenoid driven positive displacement pumps that deliver liquid in 10 µL increments. A Raspberry Pi Pico RP2040 processor programmed in MicroPython is used for control, and exposes a USB serial interface for users to submit commands using either a simple vocabulary of commands or a subset of G-Code. At a total cost of $710 USD, the Sidekick offers laboratories an easy to build, easily maintained, open-source liquid dispensing system for both research and pedagogical introductions to lab automation.

## Graphical abstract

**Figure:**
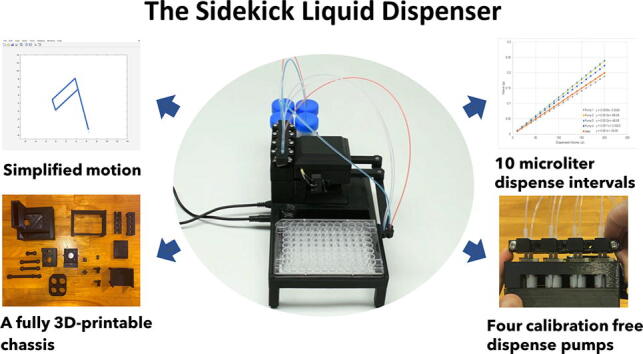

## Hardware in context

Automation increases productivity and decreases human error. Chemical laboratory automation has more than 140 years of history, evolving from hydraulic control devices in 1875 to mechanical, electromechanical, and robotic systems [1]. More recently, the idea of autonomous experimentation systems that combine automation with artificial intelligence has become an active area of research in chemistry and materials science [2], [3]. However, as devices became more complicated, it was no longer possible for benchtop scientists to build their own devices, and instrumentation became the realm of the equipment manufacturer [4]. Increased laboratory productivity and precision justified high-cost automation, leading to manufacturers focusing on more expensive but capable devices [5]. However, this can place such equipment out of reach for resource-limited, basic research and educational laboratories, reducing research productivity and training opportunities [6]. Liquid handling is one such foundational task in chemical experimentation, and commercial liquid handlers often cost tens of thousands of dollars. Opentrons offers one of the least expensive commercial solutions for automation in the form of the Opentrons OT-2, but even this machine starts at $5000 [7]. Open-source hardware can bridge the financial gap to commercial grade equipment, allowing smaller labs to experiment with automation, or acquire hardware for pedagogical purposes.

A variety of open-source liquid handling machines seek to lower the upfront cost of automation [8]–[10]. OTTO is a Cartesian liquid handling machine that uses linear rail guided motion and liquid aspiration/dispensing via micropipette. This design is entirely open source and assembled with off the shelf components for an estimated USD $1500 [8]. OTTO addresses the reliability concerns of DIY devices by adding additional sensors that detect possible alignment issues in sample preparation [4]. FINDUS uses a similar Cartesian movement system guided by a less expensive linear bearing system. By combining this bearing system with 3D-printed parts, FINDUS reduces the reported build cost to less than USD $400 [9]. PHIL is a microscope stage-top liquid handling robot, designed for microfluidics and biological research. Unlike OTTO and FINDUS, PHIL uses a 3D-printed robotic arm, and open-source peristaltic pumps [10].

While these designs have impressive capabilities at a low cost, they often require tens of hours of build time, the sourcing of dozens of parts, and construction and maintenance of the liquid dispensing systems [8], [9]. These obstacles can both reduce likelihood of adoption and increase the true cost of the system. Additionally, both OTTO and FINDUS rely on a micropipette for liquid dispensing. The micropipette adds an extra hidden cost to the final project; a P200 micropipette from a reputable manufacturer costs $170-$360 [11], [12]. While PHIL sidesteps these issues with multichannel peristaltic pumps, the time to assemble and calibrate multiple microfluidic pumps may be intimidating to the end user. To address these issues, we developed the Sidekick, a fully 3D-printed liquid dispensing robot designed for use in research and education. The Sidekick increases the approachability of open-source liquid handling automation by replacing the linear Cartesian movement system with a simpler armature-based system. It delivers liquids with four solenoid-driven displacement pumps, removing the need for assembly. These parts are combined with a fully 3D-printed chassis and a MicroPython control system. The Sidekick costs $710 to build and offers a balance between ease of use and affordability.

## Hardware description

### Hardware overview

The Sidekick’s design prioritizes simplicity of hardware, software, and assembly. Most open-source liquid handlers use Cartesian gantry motion systems like those in 3D printers [8], [9], [13], [14]. These designs are well suited for many automated liquid handling tasks, but their build complexity may be daunting for labs looking to experiment with automation. These designs often use commercial micropipettes for liquid handling, adding additional maintenance and cost that is often not included in the bill of materials estimates [8], [9].

The Sidekick addresses these issues by replacing the Cartesian kinematic system with an armature-based design, which lowers part number and increases the number of 3D-printable components. This kind of armature system was inspired by drawing robots and greatly decreases the number of components required for planar motion. The reduced mechanical complexity in turn lowers build time, machine size, and materials cost. The motion system of the Sidekick costs $152, the entire machine requires the end user to source only 19 different parts, and can be assembled in under four hours (excluding the time to 3D-print the parts). Including the total working area of the armature, the Sidekick is 275 mm long, 215 mm wide, 161 mm tall, which allows it to be used in space-constrained environments.

To keep the liquid dispensing subsystem simple, the Sidekick uses four commercial micropumps that dispense increments of 10 μL upon a voltage pulse cycle. Using four pumps allows the Sidekick to dispense four different liquids. The primary advantage of commercial micropumps is that they require no calibration, greatly simplifying the initial construction and ongoing maintenance. Should the user damage the liquid handling system (e.g., by using corrosive materials), they are modular and can be replaced. These pumps are included in the bill of materials, eliminating any hidden costs associated with adapting laboratory micropipettes. However, if flowrate or accuracy does not meet the user’s needs, the Sidekick could be adapted to use other multiple open-source options, such as the Ender 3 adapted syringe pump [15], the Poseidon syringe pump [16], or the FAST peristaltic pump [17]. Finally, we note that the Sidekick is designed only to dispense liquids, and not perform liquid aspiration (as with FINDUS, OTTO, or OpenTron OT-2). This eliminates the need for disposable tips or tip washing, and is sufficient to conduct many types of chemical synthesis experiments. However, the lack of aspiration precludes performing serial dilutions or other types of transfers between sample wells.

The Sidekick is controlled by a Raspberry Pi Pico and is programmed in MicroPython, a subset of Python 3 designed for microcontrollers [18]. Kinematic calculations are calculated on the Pico and directly translated into armature angular position. The Sidekick takes text commands via the serial port and thus can be controlled from any programming language that has access to the serial port on a host computer. It accepts commands both in a simplified format (in which the user specifies which pump to use, the desired location, and volume to dispense) as well as a subset of G-Code commands.

The chassis of the Sidekick is entirely 3D-printed in PETG or PLA, reducing material costs, part sourcing time and allowing users to customize part dimensions to incorporate imaging, spectroscopy, or other functionality. Altogether, these design choices make the Sidekick a liquid handling robot with:

• Low mechanical complexity and part number.

• Assembly time under four hours.

• Four-channel dispense system.

• Cost under $710.

2.2: Dispense Armature.

**Armature Design**: The Sidekick is designed as a 2 DOF parallel manipulator with a four bar linkage system inspired by designs like Line-us [19], Tracey.io[20], and the Mantis Liquid Dispenser [21]. Such a manipulator is more effective for our purposes than a cartesian robot, or a SCARA arm (which has a powered joint at the elbow) [22] as parallel manipulators allow for the driving motors to be mounted at the base of the linkage system [23], reducing the weight of the armature, and a four bar linkage allows the motors to be mounted in the same axis, reducing the footprint of the device. These types of robots are mechanically simple, lowering cost and build time. In contrast, most consumer grade 3D printers and open-source liquid handlers share the architecture of a 3-axis Cartesian robot [8], [9], [13], [14]. The Cartesian design maximizes stability and precision, and the orthogonal axis makes kinematics calculations trivial. While the popularity of 3D printing hardware has lowered the cost to create these types of robots, they still have significant upfront price and build time. Builders must source linear bearings, pulleys, and motors to accomplish linear motion. If a user is only dispensing onto a single (ANSI SLAS 4–2004 [24]) SBS microplate, the creation of such a large machine is unnecessary. In contrast, the Sidekick’s design requires four 3D-printed arms, three ball bearings, and two stepper motors. Positional accuracy is achieved by implementing a homing system, and high-resolution stepper motors.

This design is well suited to dispensing into a single 96-well SBS microplate, and the increased complexity of motion programming inherent to the linkage system is hidden to the end user. The inverse and direct kinematics functions are preprogrammed, and the experimentalist can simply direct the end effector to a desired position using the standard nomenclature for SBS microplate positions. The smaller operating area reduces the amplification of positional error that would occur with a longer armature. The lack of any z-axis motion needed for aspiration or tip ejection alleviates the need for strict end effector rigidity.

Fig. 2 shows the usable area of the dispense arm, and Fig. 3 shows a schematic of the dispense arm, which consists of four 3D-printed arms, connected at each joint by 10 mm ball bearings. The base of the armature is connected to two stepper motors. These motors are responsible for moving the dispense head across the usable area. Such a design allows for any reachable location to be described by the angular position of Arm One and Arm Two.

**Fig. 1:**
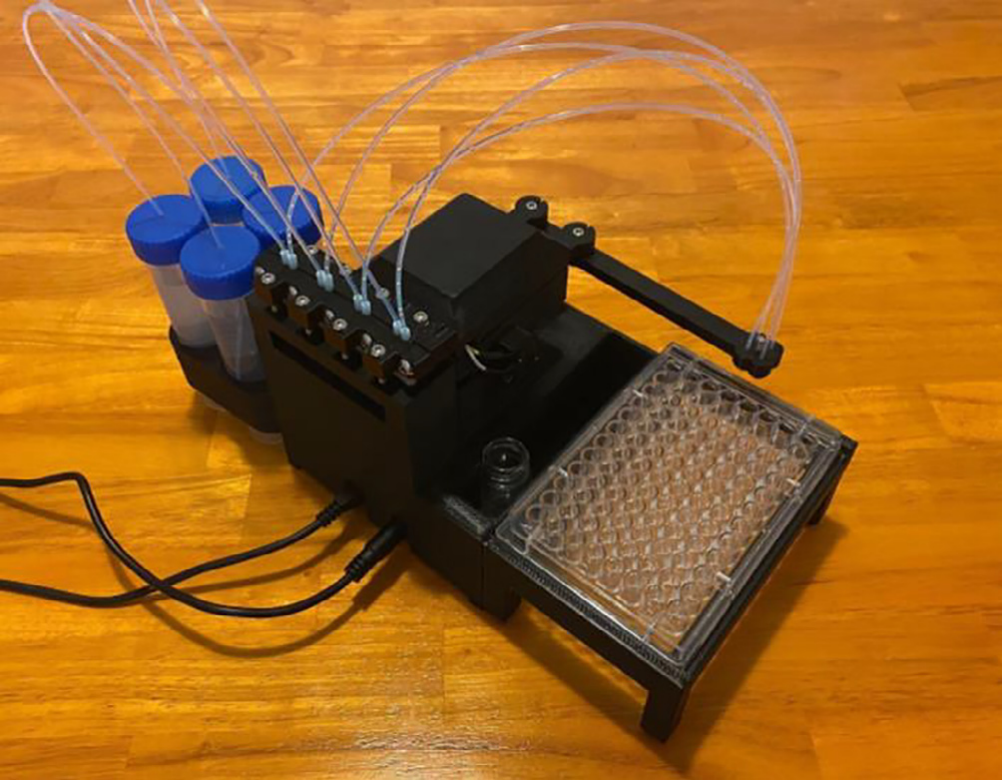
The Sidekick Liquid Handler.

**Fig. 2:**
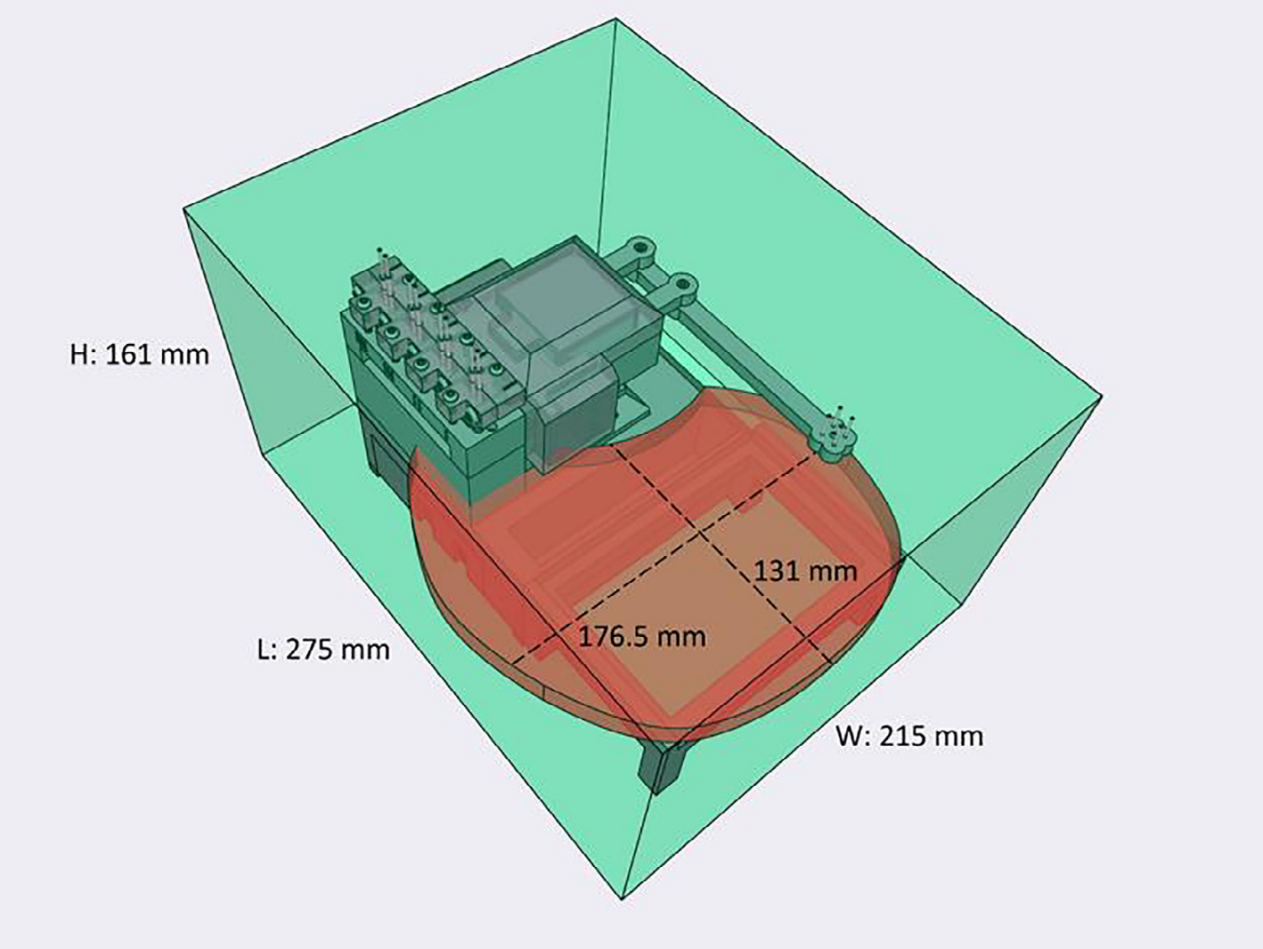
A visual representation of the Sidekick’s working area. The red area denotes the area reachable by the dispensing armature (131 mm × 176.5 mm). The green box illustrates the minimum area needed to contain the Sidekick’s movement (275 mm × 215 mm × 161 mm). (For interpretation of the references to color in this figure legend, the reader is referred to the web version of this article.)

**Fig. 3:**
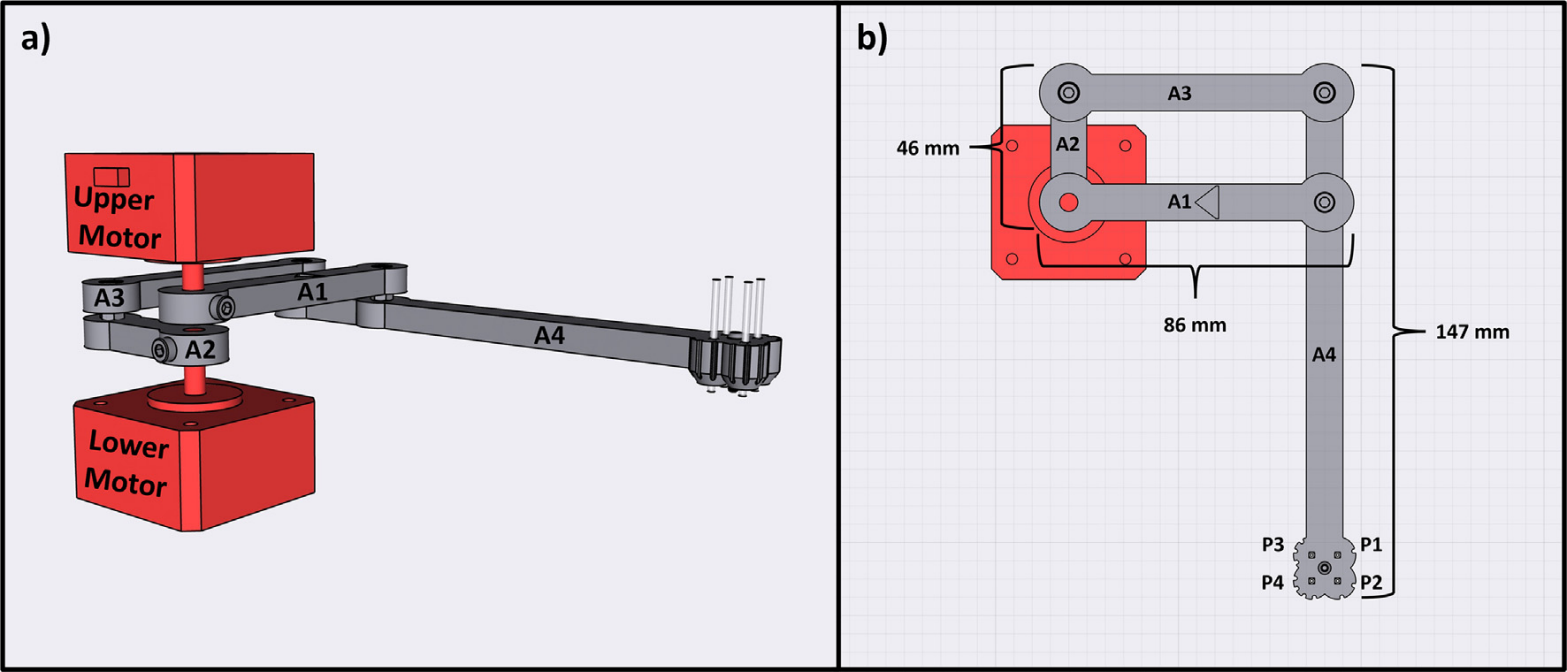
Diagram of the Sidekick’s dispense armature. (a) Schematic of the armature linkage. (b) Dimensions of the armature and the location of the dispense nozzles. Liquids can be dispensed from P1, P2, P3 or P4.

**Fig. 4:**
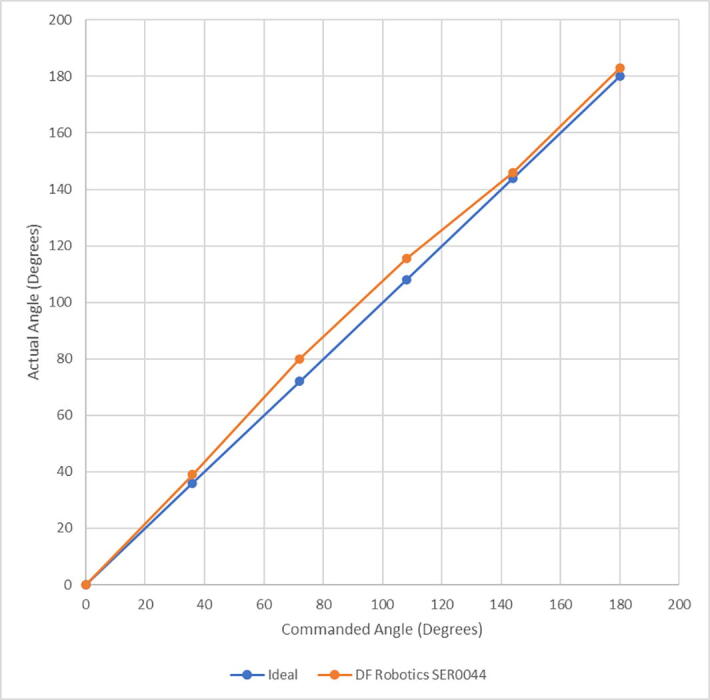
DF Robotics SER0044 Experimental vs. Commanded Angle, Nonlinearity. In contrast, the deviation from linearity of the NEMA 17 stepper motors used in the final build is indistinguishable from ideal at this scale.

**Motor Choice:** The difference in motor angular positioning between pump placements over each well can be as low as 2.2°. This difference is derived from the inverse kinematics calculations that convert between cartesian coordinates of well position to angular position of the motor shafts. For example, the motor driving Arm 1 is at 98.9° from zero when pump 1 is positioned over well A1, and at 101.1° when pump 2 is positioned over well A1. Because of these small differences in angular position, a motor with high angular resolution is necessary. Angular resolution of the motors was measured using a compass and a 3D printed attachment to the shaft of the motor. We initially considered servo motors, as they simplify control and do not require any additional drivers or encoders, but we found typical hobbyist servo motors did not provide the requisite angular resolution. Backlash in the gearings, and an imprecision in the encoders of ± 1.5° precluded their use. We tested the MG90S and the SER0047 micro servos and found that their gearing backlash exceeded 1°. When attempting to position the armature over the 96-well plate, the end effector would vary from the center of each well by more than 3 mm. The backlash and the low angular resolution of the encoders made it impossible to account for this inaccuracy by any computational method. We then tested the larger Feetech 3443, TowerPro SG5010, and the DF Robotics SER0044 servo motors. While these larger servos did not have any measurable backlash, their positional feedback suffered from noticeable non-linearity (Fig. 4). While this non-linearity could be accounted for by applying a regression and correcting the positional command, this would have to be calibrated by the end user, which seemed impractical.

Despite the need for an additional driver and the loss of closed loop control, this led us to consider NEMA 17 pancake stepper motors, as they offer precise motion and a small footprint. Choosing a stepper motor with a step-angle of 0.9° offers a baseline angular resolution that meets the Sidekick’s requirements. Micro-stepping enables even greater angular resolution, opening the possibility of using 1.8° stepper motors to further save on cost. In testing, these 0.9° steppers with 1/8 micro stepping reliably centered the end effector over each well within 1.0 mm precision. The loss of closed loop control, and the possibility of skipping steps are not concerns due to the low resistance to armature motion. The pancake stepper motors run on 12–24 V, and are rated at 1.2A per phase, with a holding torque of 11 N/cm. The Sidekick drives these steppers at 12 V at 1A per phase. We excluded larger robotics-oriented servos with the required angular resolution, such as the Dynamixel XL-320 for two reasons. First, the servos are each $8 more expensive than the stepper motors considered here, and also require a proprietary driver [25] that is $10 more expensive than the stepper motor; this would raise the cost of the motion subsystem from $151 to $177, a 17% increase.

2.3: Motor Control.

**Driver Choice:** Stepper drivers were chosen based upon footprint, power requirements, and operational noise. Several commonly used and widely available drivers were tested to explore the tradeoff between cost and functionality.

Adafruit TB6612: This is a popular hobbyist driver that is quiet in operation but lacks an adjustable reference voltage (V-Ref). V-Ref allows the user to regulate the amount of current sent to the motor. Without the adjustable current, motor overheating was observed in 30-minute continuous operation manifesting in skipped steps and high motor surface temperature.

Allegro A4988: This is a dedicated stepper driver, offering adjustable V-Ref and higher micro stepping capabilities. While the adjustable max current solves the overheating problems, the A4988 suffers from high operational noise. The driver whine during movement and holding torque is very audible. While driver noise can be mitigated by reducing current and increasing micro stepping [26], neither option was able to reduce the noise to the level of the TMC 2209 (vide infra).

TI DRV8825: This is also an acceptable option with smooth reliable motion and good thermals, but like the A4988, it suffers from high operational noise that cannot be mitigated by micro-stepping or reducing current. While it may be possible to increase micro stepping past the 1/64th steps that were tested, the Pico’s processor becomes the limiting factor and skips steps. This could potentially be solved using the Pico’s programmable IO pins, but with an increase in programming complexity.

TMC 2209: This was the best choice in thermals, noise, and accuracy. These drivers lack any noticeable noise in operation, offer high micro stepping capabilities, and adjustable V-Ref. These were the final choice for the Sidekick’s drivers. These drivers are used in conjunction with two limit switches that mark the home position of the dispense armature to drive precise and accurate motion. Additionally, these drivers feature stall protection, which allows for the possibility of adding feedback to increase reliability and eliminating the need for mechanical limit switches during the homing process in future iterations of the design.

**Kinematics:** The Sidekick’s kinematics calculation uses a preset lookup table that converts between 96-well SBS plate locations and stepper motor angular position. To accommodate for variations in assembly and plate placement, the Sidekick can generate plate maps for any N × M array of wells. This is used to either fine tune effector placement in case the preset lookup table is not sufficiently accurate, or to generate a new map for a different configuration of wells. After calibrating by centering the effector over each of the four corner wells, linear interpolation is used to determine the location of the remaining wells.

For fine tuning effector placement and manual movement, the Sidekick calculates the kinematics on the Raspberry Pico. This allows the use of non-standard plates or more bespoke applications that require precise locational placement. The kinematics are calculated for each of the four nozzles, and the center point of the effector. These algorithms are all written in MicroPython, and the Sidekick’s motion is controlled via commands read from the USB serial port.

2.4: Pump System.

Four main options were considered when choosing pumps: Micropipette adaptations, open-source peristaltic pumps, open-source positive displacement pumps, and commercial positive displacement pumps. Below we discuss the tradeoff between cost, complexity, and precision.

The adaptation of handheld micropipettes for liquid dispensing robots is a common design choice [8], [9]. While micropipettes offer excellent precision and accuracy, their adaptation for automated use is both expensive and calibration heavy. Additionally, to aspirate, dispense, and change tips requires z-axis mobility. This would add additional cost to the motion system and increase calibration times for the end user.

Peristaltic pumps are ubiquitous in open-source lab automation hardware systems [10], [27], [28], [29] and their design is described in standalone hardware articles [17], [30], [31]. Given their support and performance, they were seriously considered for the multichannel pumping system. These pumps can be purchased, but there are also open-source pump designs, like the FAST, which offers flow rates as low as 0.7 µL/min [17], [30]. In theory, this low flow rate would allow for microliter-level accuracy during dispensing. Ultimately, the time required to build and calibrate these peristaltic pumps was the deciding factor. As the Sidekick is equipped with four channels, these pumps would have to be built and calibrated multiple times. At a price of $50 per pump, the savings did not justify the increase in complexity and build time [30].

Open-source positive displacement pumps offer wider functionality than pumps. Their ability to aspirate through a disposable pipette tip allows for the intake of liquids and widens functionality beyond dispensing. There are numerous open-source vacuum displacement pump options, but the current open-source designs are rarely tested to volumes under 10 µL [15], [16]. These pumps often utilize glass syringes or plastic coupled with a stepper motor and a lead screw to drive linear motion, so they are less mechanically complex than peristaltic pumps [15], [16], [31], [32].

The commercial positive displacement pumps from the Lee Company were ultimately chosen for their best out of the box performance. The LPM series of pumps are a range of chemically inert, solenoid driven positive displacement pumps. The pumps are energized by a 12-volt square wave, and aspirate fluid from the inlet when energized then dispense when de-energized. The wetted materials of the pumps are a combination of polyether ether ketone (PEEK) and fluoro elastomer (FKM) [33]. The Sidekick is equipped with four of the 10 μL variants (LPMA1250110L). As we wanted to focus on accessibility and cost, the use of calibration free commercial displacement pumps offering 10 μL aliquots with ± 15% manufacturers tolerance struck a balance between performance and ease of use [34]. Given the comparable performance and cost of open-source peristaltic pumps, the LPM pumps offer the requisite accuracy while skipping the development and calibration of assembling an open-source design. The 10 μL aliquot offers sufficient granularity to perform a wide variety of dispensing tasks, considering that a standard [24] SBS 96-well plate typically has a functional volume range of 80 to 340 μL. A disadvantage of selecting this type of pump, in contrast to the alternatives discussed above, is the lack of sample aspiration capability—it only aspirates from a fixed source reservoir and dispenses to the selected target destination. While this prevents the use of our device for certain types of experiments (for example, dilution series) many types of automated chemical experiments only need liquid dispensing (for example, synthesis of halide perovskites [35]). The Sidekick uses 1/32″ ID PTFE tubing for liquid pathing from the reservoir and dispensing into the target well. This narrow tubing serves two purposes. The narrower tubing reduces cross sectional area at the dispense tip, decreasing the possibility of dripping during travel motions, and is more flexible, reducing the resistance on the armature when bending the tubing during travel.

2.4: Electronic Components.

**Fig. 5:**
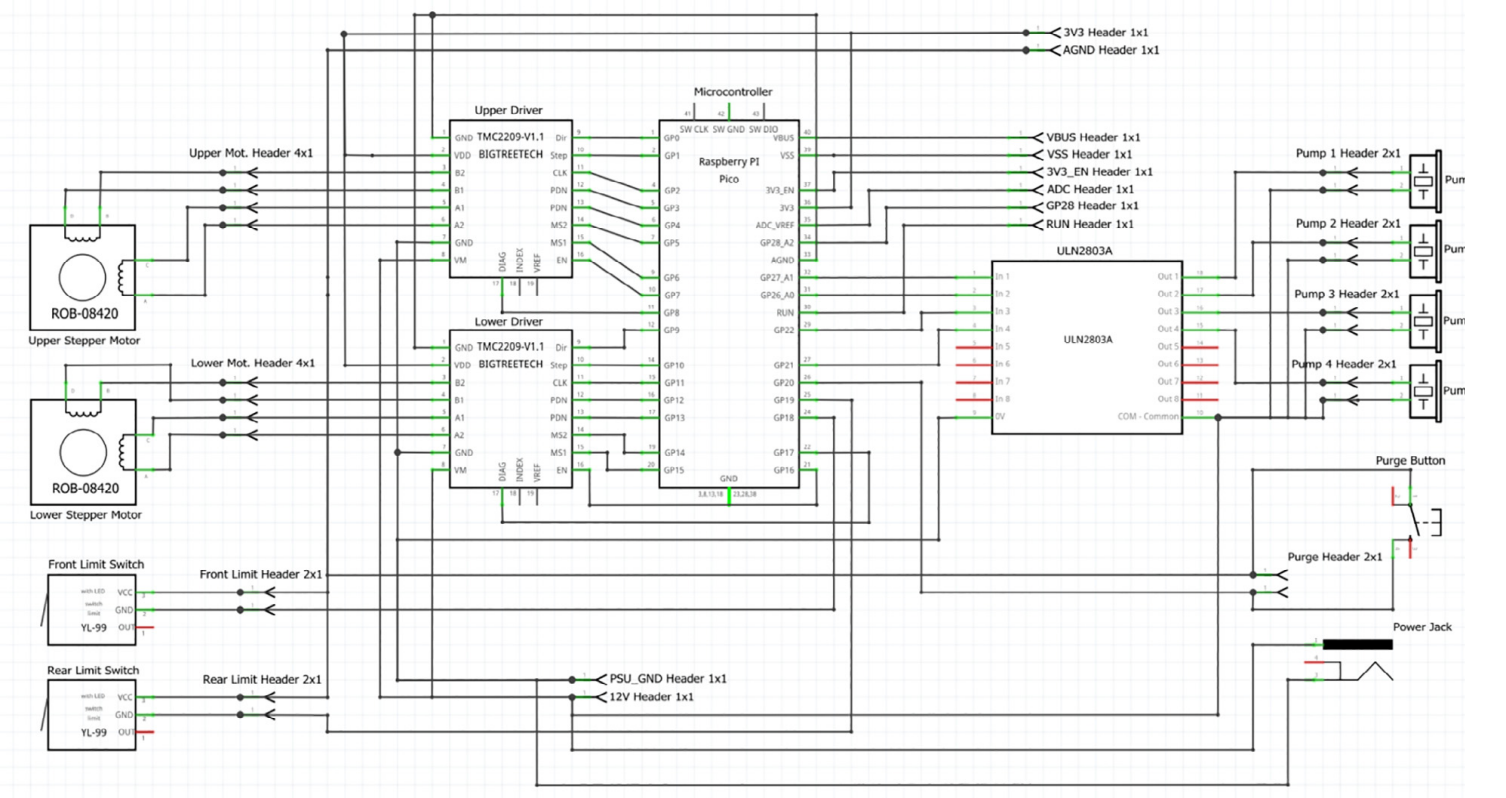
Electrical schematic.

An electrical schematic is shown in Fig. 5. The Sidekick is controlled by a Raspberry Pi Pico running MicroPython [36]. Each of the two stepper motors are driven by a TMC 2209 stepper driver. The four pumps are driven by a ULN2803A Darlington Array. The remaining unused pins of the Pico are routed out to an accessible header pin through holes, so that the end user can add additional hardware. The Pico is powered by the 5 V USB bus, and the stepper motors and pumps are powered by a generic 12-volt, 5-amp power supply. The components are mounted on a custom PCB designed in Fritzing [37].

## Design files summary

**Table:** 

**Design file name**	**File type**	**Open source license**	**Location of the file**
Base	STL		https://github.com/rodolfokeesey/Liquid-Handler/tree/main/3D_Assets/MainBody
Button Housing	STL		https://github.com/rodolfokeesey/Liquid-Handler/tree/main/3D_Assets/MainBody
Button Housing Front	STL		https://github.com/rodolfokeesey/Liquid-Handler/tree/main/3D_Assets/MainBody
Foot	STL		https://github.com/rodolfokeesey/Liquid-Handler/tree/main/3D_Assets/MainBody
PCB Tray	STL		https://github.com/rodolfokeesey/Liquid-Handler/tree/main/3D_Assets/MainBody
Arm One	STL		https://github.com/rodolfokeesey/Liquid-Handler/tree/main/3D_Assets/Armature
Arm Two	STL		https://github.com/rodolfokeesey/Liquid-Handler/tree/main/3D_Assets/Armature
Arm Three	STL		https://github.com/rodolfokeesey/Liquid-Handler/tree/main/3D_Assets/Armature
Arm Four	STL		https://github.com/rodolfokeesey/Liquid-Handler/tree/main/3D_Assets/Armature
Upper Motor Mount	STL		https://github.com/rodolfokeesey/Liquid-Handler/tree/main/3D_Assets/MotorMounts
Lower Motor Mount	STL		https://github.com/rodolfokeesey/Liquid-Handler/tree/main/3D_Assets/MotorMounts
Motor Cap	STL		https://github.com/rodolfokeesey/Liquid-Handler/tree/main/3D_Assets/MotorMounts
Front Switch Mount	STL		https://github.com/rodolfokeesey/Liquid-Handler/tree/main/3D_Assets/MotorMounts
Rear Switch Mount	STL		https://github.com/rodolfokeesey/Liquid-Handler/tree/main/3D_Assets/MotorMounts
FrontAdapter Clamp	STL		https://github.com/rodolfokeesey/Liquid-Handler/tree/main/3D_Assets/Pumps s
RearAdapter Clamp	STL		https://github.com/rodolfokeesey/Liquid-Handler/tree/main/3D_Assets/Pumps
Pump Mount	STL		https://github.com/rodolfokeesey/Liquid-Handler/tree/main/3D_Assets/Pumps
96 Well Plate Holder	STL		https://github.com/rodolfokeesey/Liquid-Handler/tree/main/3D_Assets/96WellTrayHolder_Reservoir
50 ml Tube Holder	STL		https://github.com/rodolfokeesey/Liquid-Handler/tree/main/3D_Assets/96WellTrayHolder_Reservoir
SidekickV3	fzz		https://github.com/rodolfokeesey/Liquid-Handler/tree/main/PCB
SidekickV3	zip		https://github.com/rodolfokeesey/Liquid-Handler/tree/main/PCB
Pico Snapshot	folder		https://github.com/rodolfokeesey/Liquid-Handler/tree/main/Pico_Snapshot
Serial Example Putty	pdf		https://github.com/rodolfokeesey/Liquid-Handler/blob/main/Supporting_Documentation/Serial_Example_Putty.pdf

Item Descriptions:

**Base:** The base of the Sidekick liquid handler. The purge button, motor assembly, pump assembly, and plate tray are attached to the base.

**Button Housing:** Houses the purge button and attaches to the base.

**Button Housing Front:** Covers the front of the purge button and secures it into place.

**Foot:** Standoff feet that attach to the bottom of the base and plate holder. Eight (8) copies of this should be printed.

**PCB Tray:** Holds the PCB and connects to the bottom of the base.

**Arm One:** The first arm of the armature movement system, this arm is attached to the upper stepper motor.

**Arm Two:** The second arm of the armature movement system, this arm is attached to the lower stepper motor.

**Arm Three:** The third arm of the armature movement system, this arm is attached to arms two and four.

**Arm Four:** The fourth arm of the armature movement system, this arm is attached to arms three and one. The end of this arm houses the multichannel dispenser.

**Upper Motor Mount:** Houses the upper stepper motor, and the front and rear limit switches.

**Lower Motor Mount:** Houses the lower stepper motor and attaches directly to the base.

**Motor Cap:** Sits on top of the upper motor mount to hide exposed wiring.

**Front Switch Mount:** Houses the front limit switch, attaches to the upper motor mount.

**Rear Switch Mount:** Houses the rear limit switch, attaches to the upper motor mount.

**Front Adapter Clamp:** Front clamp to adapt the pump outlet to the smaller diameter output/input tubing.

**Rear Adapter Clamp:** Rear clamp to adapt the pump outlet to the smaller diameter output/input tubing.

**Pump Mount:** Houses up to four LPM pumps and attaches them to the base.

**96 Well Plate Holder:** Holds a SBS plate for liquid dispensing and attaches to the base.

**50 ml Tube Holder:** Holds four 50 ml falcon tubes, used as a reservoir for dispensing.

**SidekickV3.fzz:** A PCB blueprint in the Fritzing software’s proprietary format.

**SidekickV3.zip:** The PCB files in a compressed format. This is uploaded to a PCB manufacturing service for production.

**Pico Snapshot:** A folder containing all the Python scripts necessary for running the Sidekick. These files are uploaded onto the Pico.

**Serial Example Putty:** A pdf file with an example of how to connect to the Sidekick via serial using Putty.

Bill of materials summary.

See Attached Excel Spreadsheet. The cost was last updated on 18 Feb 2022; a previous version prepared in November 2021 priced the materials at <$650.

Build instructions.

5A: Software/Hardware Set Up.

Step 1: Install MicroPython onto Pico.

Flash the Raspberry Pi Pico with MicroPython. Follow the directions in this link:

https://www.raspberrypi.org/documentation/microcontrollers/micropython.html.

Step 2: Install Thonny IDE.

After flashing MicroPython onto the Pico, we need to upload the Sidekick’s code to the microcontroller.

Download the Thonny IDE. This will be used to load the provided code onto the Pico.

https://thonny.org/.

Once downloaded, install, and follow the prompts for set up with MicroPython and the Raspberry Pi Pico.

Step 3: Download the Sidekick snapshot.

Go to the Sidekick GitHub page to download all the necessary files:

https://github.com/rodolfokeesey/Liquid-Handler.

Go to the folder marked “Pico Snapshot” and download the contents. Now return to Thonny. Navigate to View -> Files.

Step 4: Upload the Sidekick snapshot to Pico.

In the upper panel, navigate to where you downloaded the snapshot files. On this (Windows) machine it’s under:

C:\Users\User Name\Desktop\PicoSnapshot10_1_21.

Then, right click on each item in the snapshot, and click “Upload to /” in the dropdown. This saves each of the files onto the Pico.

Step 5: Opening the main loop.

Once all the files are uploaded, press the “Open Icon” then select the Raspberry Pi Pico. Open the main.py file.

Step 6: Running the main loop.

**Fig. 6:**
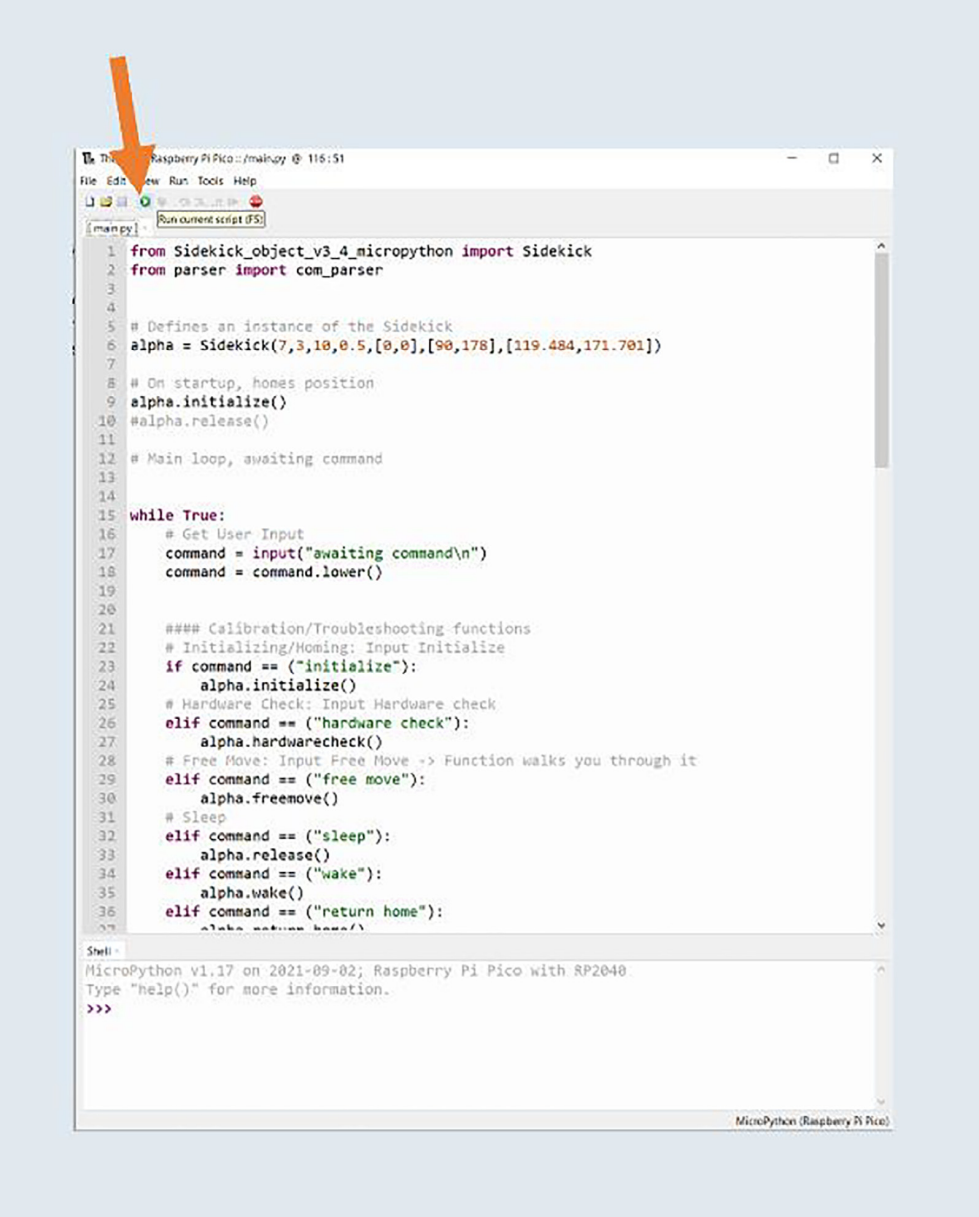
Running main.py.

Once opened, press the green “run current script” button (Fig. 6). This initializes the main loop of the robot. Because the Sidekick is not currently attached to any hardware, nothing will happen. Just disconnect the Pico from the computer. Do not press the “Stop” button in Thonny. The main loop should be left running. The Pico is now flashed with the Sidekick’s code.

Step 7: Ordering the custom PCB.

**Fig. 7:**
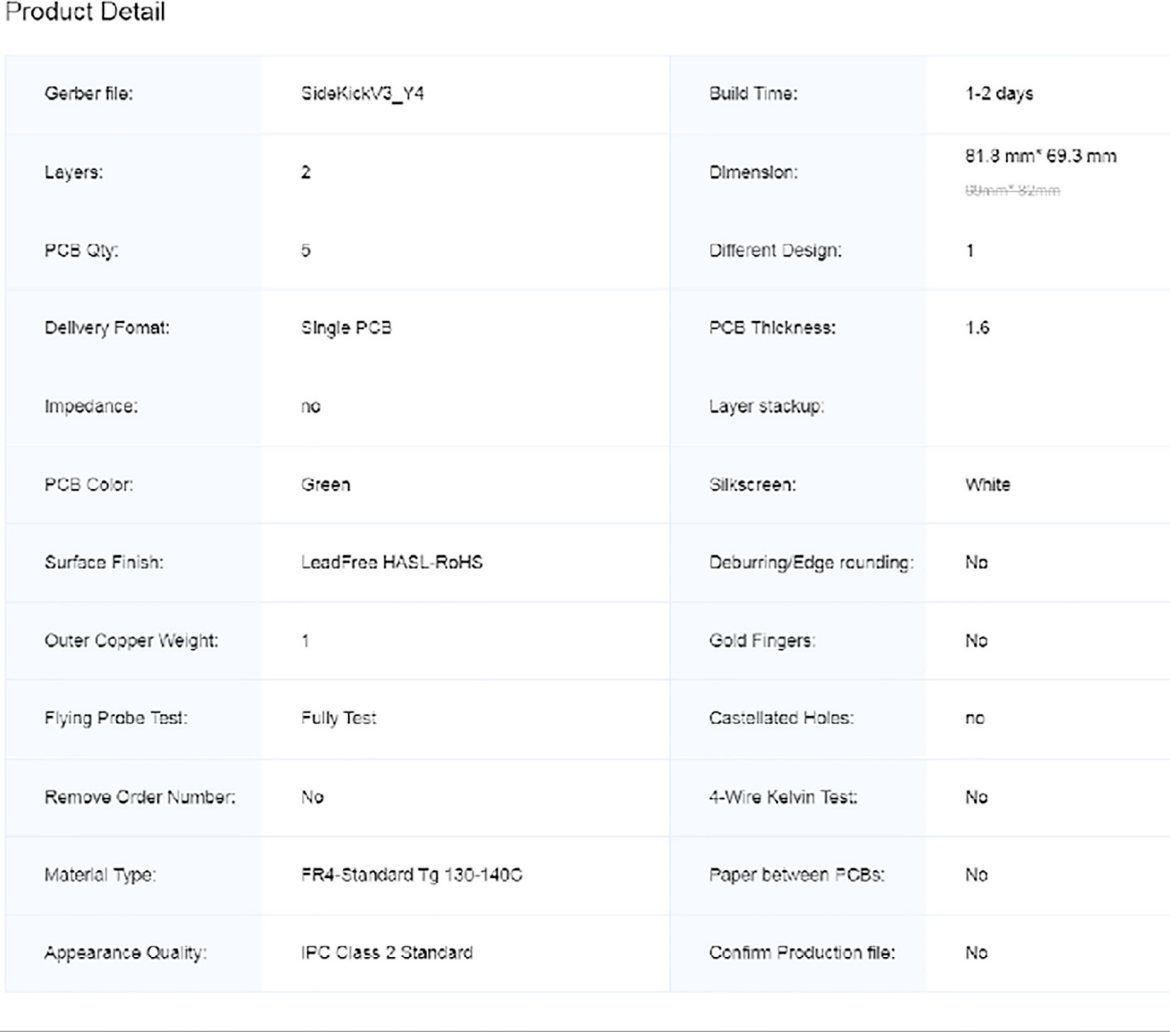
The JLCPCB order options for the Sidekick PCB.

The Sidekick has a custom PCB designed in Fritzing. The files for the PCB are in the “PCB” folder on the Sidekick’s GitHub page. We used JLCPCB to manufacture a set of 5 boards (https://jlcpcb.com/). Upload the SidekickV3.zip file to JLCPCB. The settings for the board are pictured in Fig. 7.

**Fig. 8:**
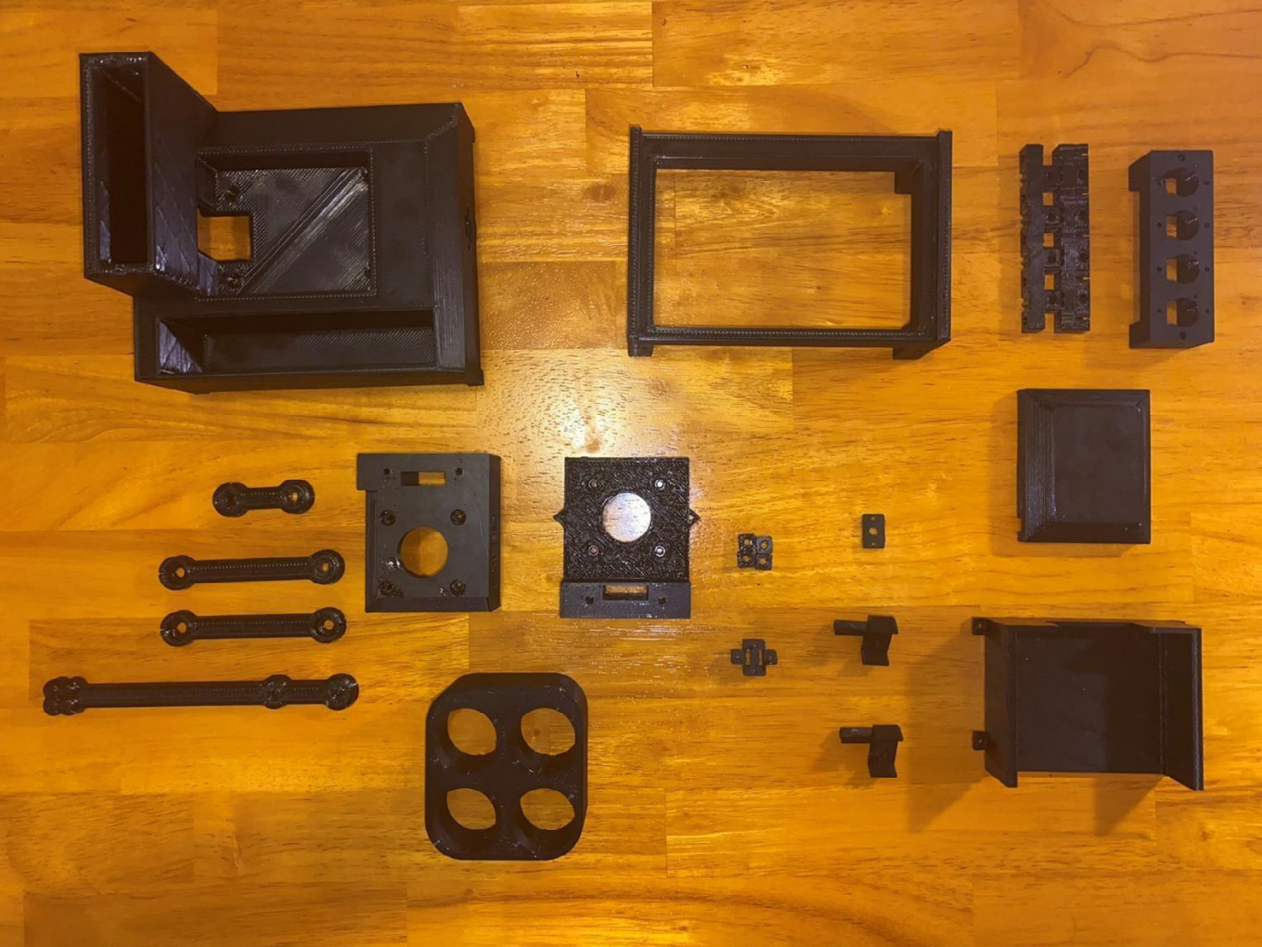
The 3D-printed parts Sidekick components.

Step 8: 3D Printing Sidekick components. (Fig. 8)

Go to the folder marked “3D Assets” and download all files. We recommend printing the models in PETG, as it has greater strength, higher temperature tolerance, and slightly better chemical resistance than the more commonly used PLA, while still being relatively easy to print. If you do not have access to PETG, you may print them in PLA, but monitor the armature for slippage against the shaft due to motor heat. Print 8 copies of the foot.stl file, and one copy of every other file.

Print with the following settings:

20% Infill.

Support Everywhere.

15% Support Density.

## Wall Perimeter, or equivalent 1.2 mm perimeter

One prototype (pictured throughout) was printed on a Creality CR10S Pro V2, using PETG. The layer heights were set to 0.42 mm on a 0.6 mm nozzle and printed with an 80 °C bed temperature and 240 °C nozzle temperature. A second prototype was printed on a Prusa MK3S using PLA with 0.35 mm layer height and 0.4 mm nozzle.

5B: Preliminary Wiring.

Step 1: Prepare wires.

**Fig. 9:**
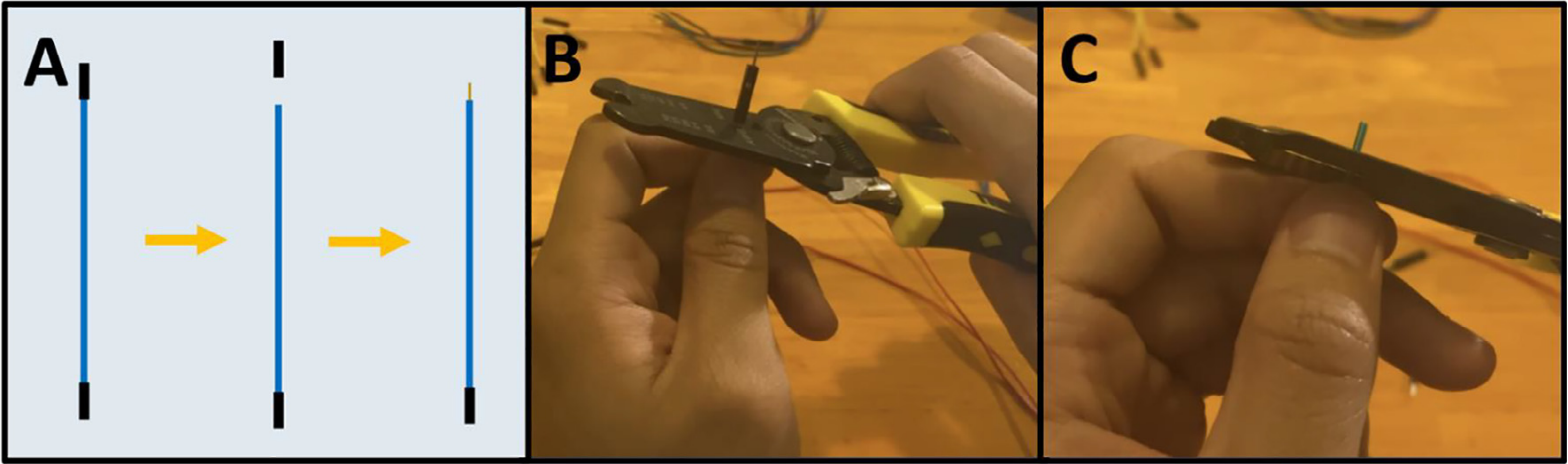
a-c: The procedure for preparing the Dupont connectors.

See Fig. 9a for an overview of the wire preparation, 12 male-to-female Dupont connectors are needed. Cut the male end from a male–female Dupont connector (Fig. 9b), then strip the insulation to expose the bare wire (Fig. 9c). Use these stripped wires for steps 2–3. **Be sure to match the wire colors in the diagram** which will allow you to easily follow the instructions for connecting the cables into the PCB.

Step 2: Splice Dupont Connectors to Stepper Motors.

**Fig. 10:**
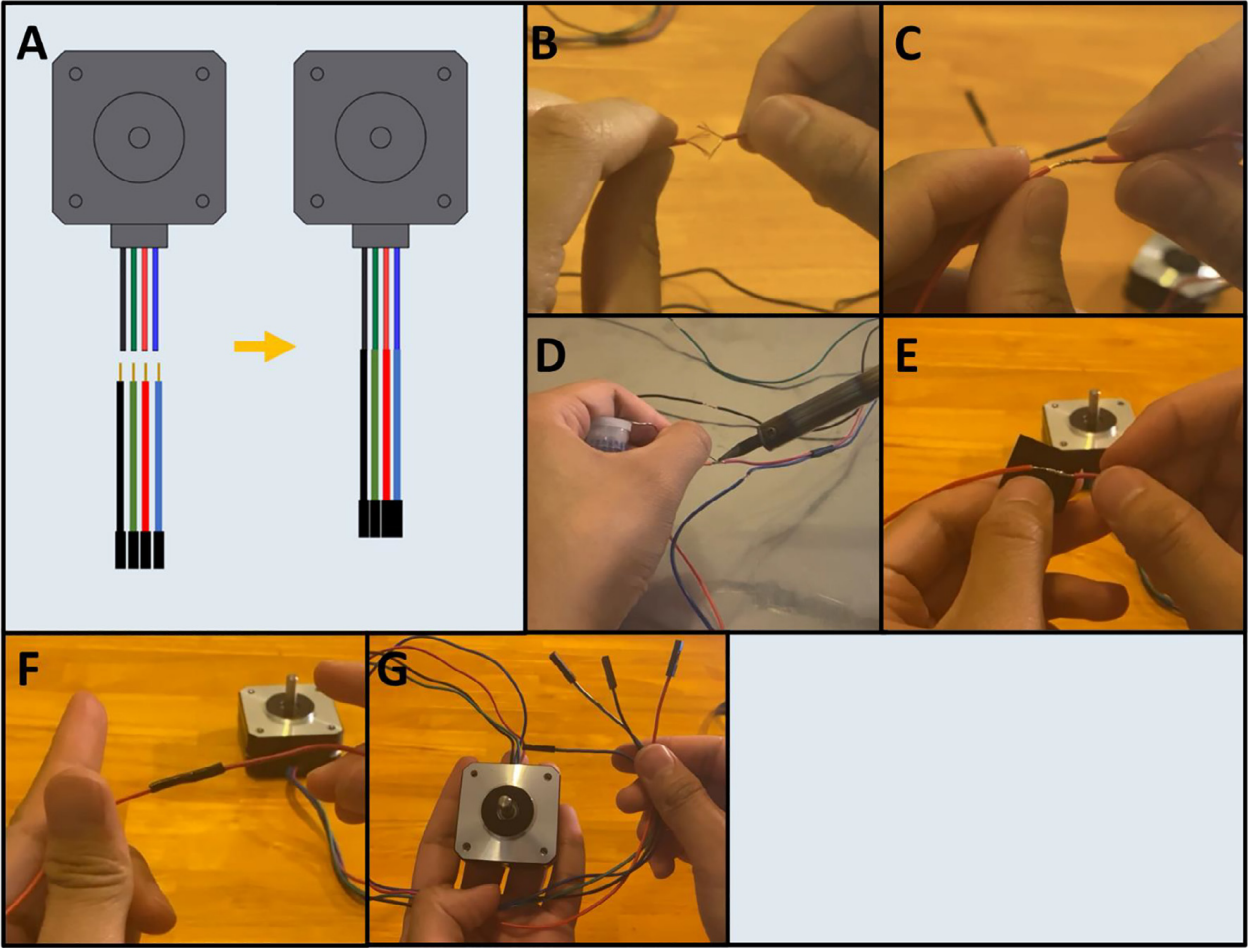
a-g: The procedure for splicing the prepared Dupont connectors to the stepper motors.

Fig. 10a gives an overview of the cable connection. Use wire strippers to strip away the insulation from the ends of the stepper motor wires. Then, splice matching colored Dupont cables prepared in Step 1 (Fig. 10b) to the stepper motor wires, the resulting connection should look like Fig. 10c. Then solder the connection (Fig. 10d) and wrap the exposed wire with electrical tape (Fig. 10e). The resulting join should look like Fig. 10f. Repeat for the remaining cables and stepper motors (Fig. 10g) This elongates the stepper motor cables and allows them to connect to the male header pins of the PCB.

Step 3: Connecting wires to the Limit Switches.

**Fig. 11:**
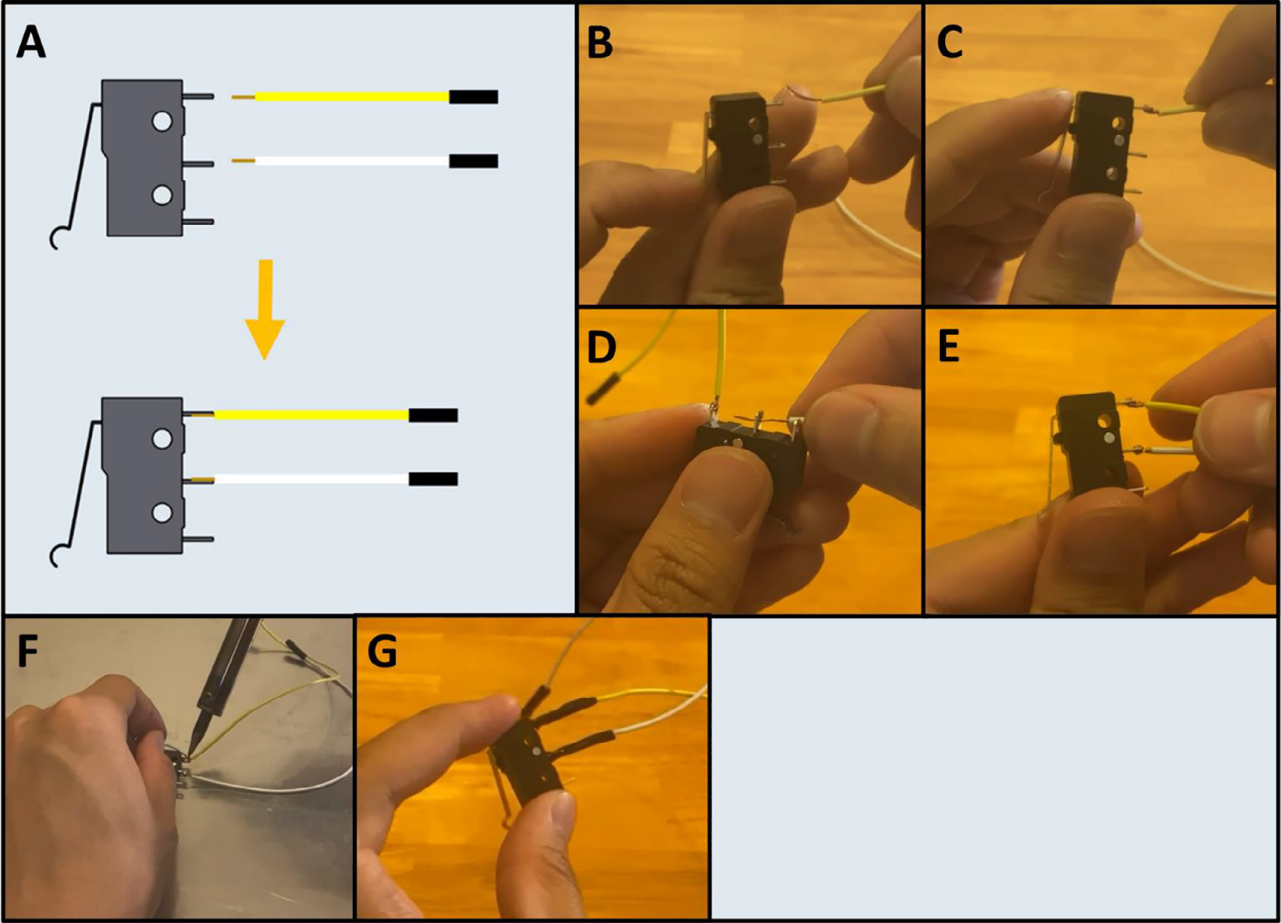
a-g: The procedure for connecting the prepared Dupont cables to the two limit switches.

Fig. 11a gives an overview of the limit switch preparation. Gather a limit switch and a prepared Dupont cable (Fig. 11b). Wrap the stripped end of the prepared cable around the exposed lead of the limit switch (Fig. 11c). Repeat with the middle lead (Fig. 11d-e). Solder both the joins (Fig. 11f) and wrap with electrical tape (Fig. 11g). Repeat for the second limit switch.

Step 4: Wiring the Purge Button.

**Fig. 12:**
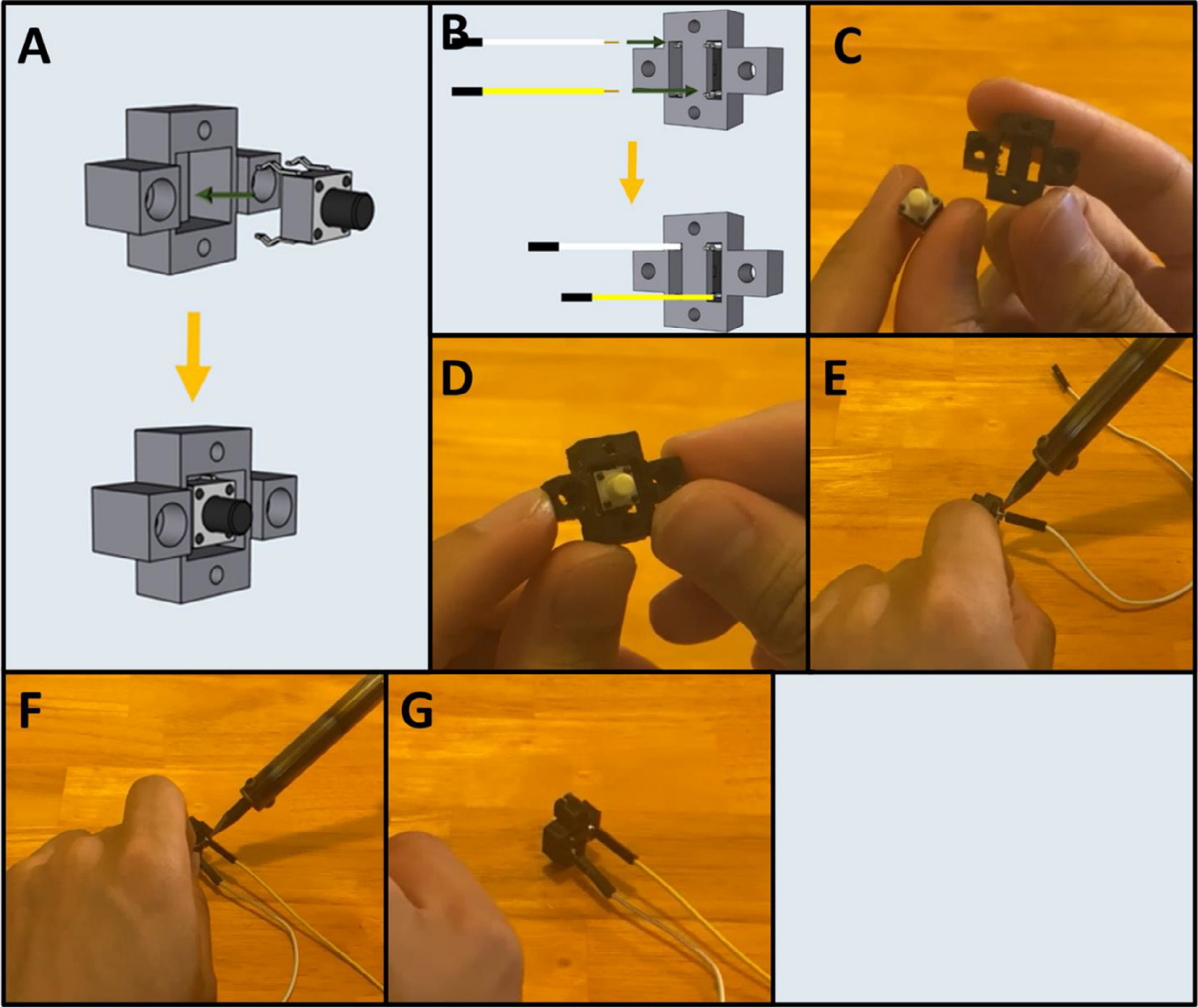
a-g: The procedure for preparing the purge button.

Fig. 12a-b gives an overview of the purge button preparation. Gather the button housing, button, and a yellow and white, male to female Dupont cable (Fig. 12c). Press the button into the button housing (Fig. 12d) and then solder the male leads of the Dupont cable to the button leads (Fig. 12e-f) in the configuration indicated on Fig. 12b. The finished button should look like Fig. 12g.

Step 5: Attaching Dupont Cables to LPL Pumps.

**Fig. 13:**
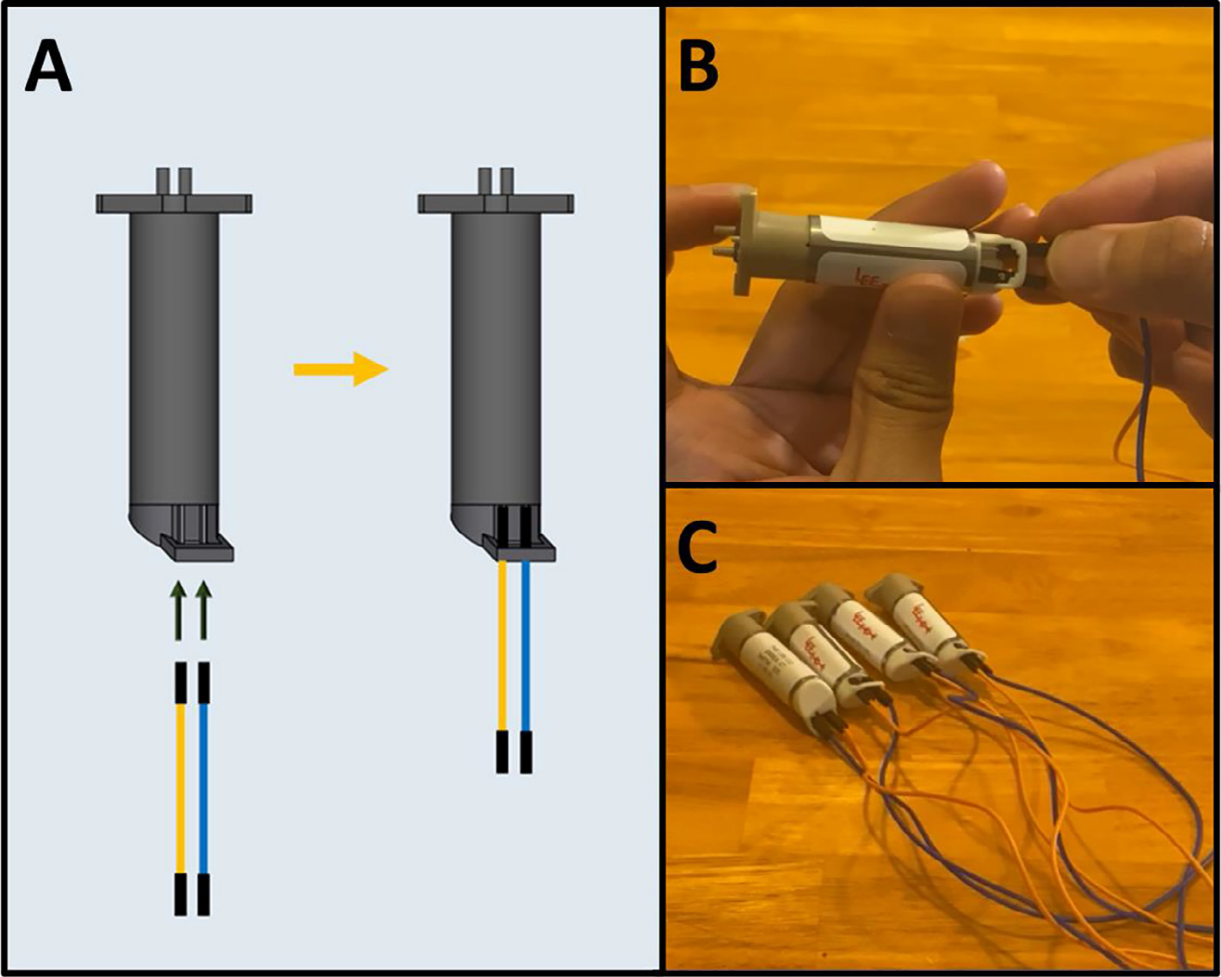
a-c: The procedure for preparing the LPM pumps.

Fig. 13a gives an overview of the pump procedure. Gather the four LPM micropumps, four blue female-to-female Dupont connectors, and four orange female-to-female Dupont connectors. Attach one blue and one orange female-female Dupont connectors onto the two contacts of the LPM pump (Fig. 13b). Follow the configuration indicated on Fig. 13a. Repeat three more times for the rest of the pumps (Fig. 13c).

**Fig. 14:**
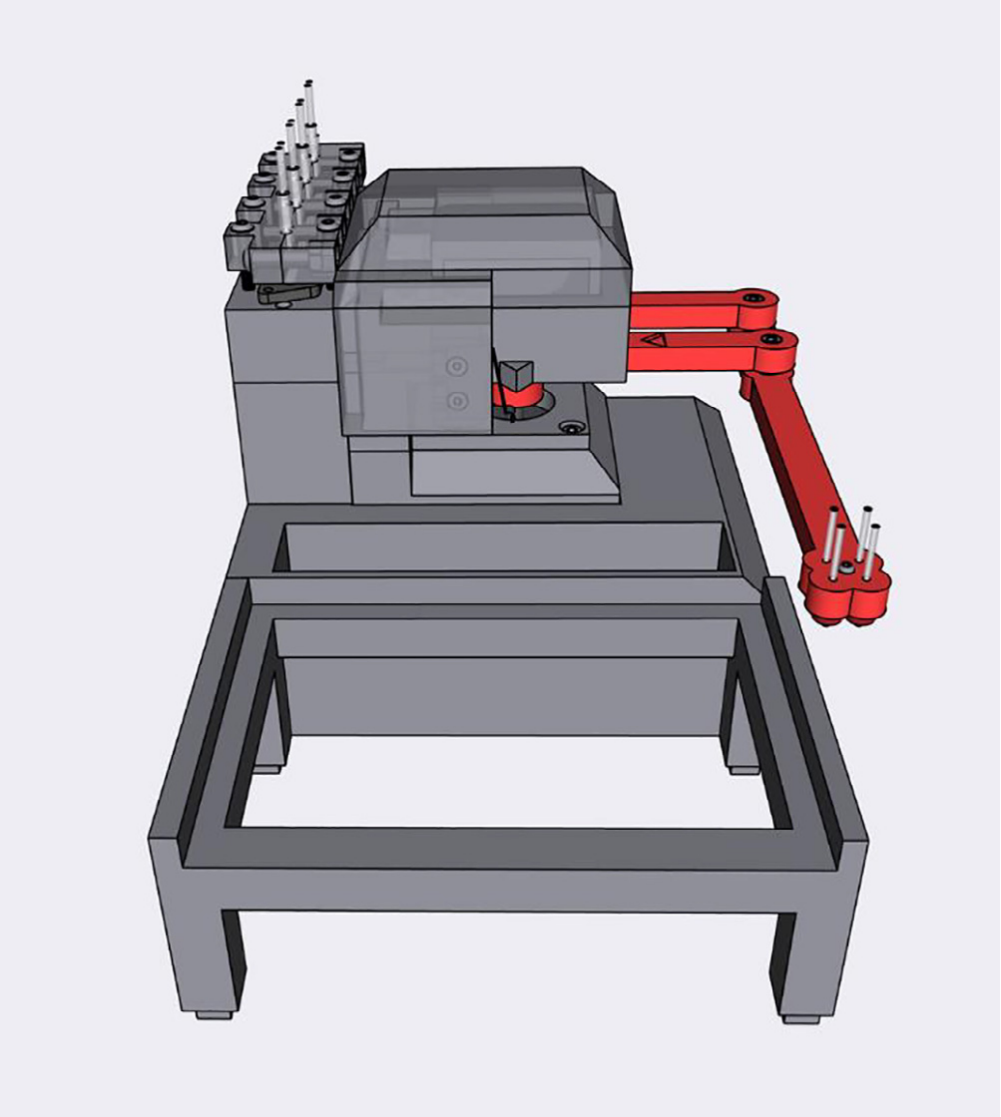
Assembling the Sidekick Armature.

5C: Armature. (Fig. 14)

Step 1: Assembling Arm One.

**Fig. 15:**
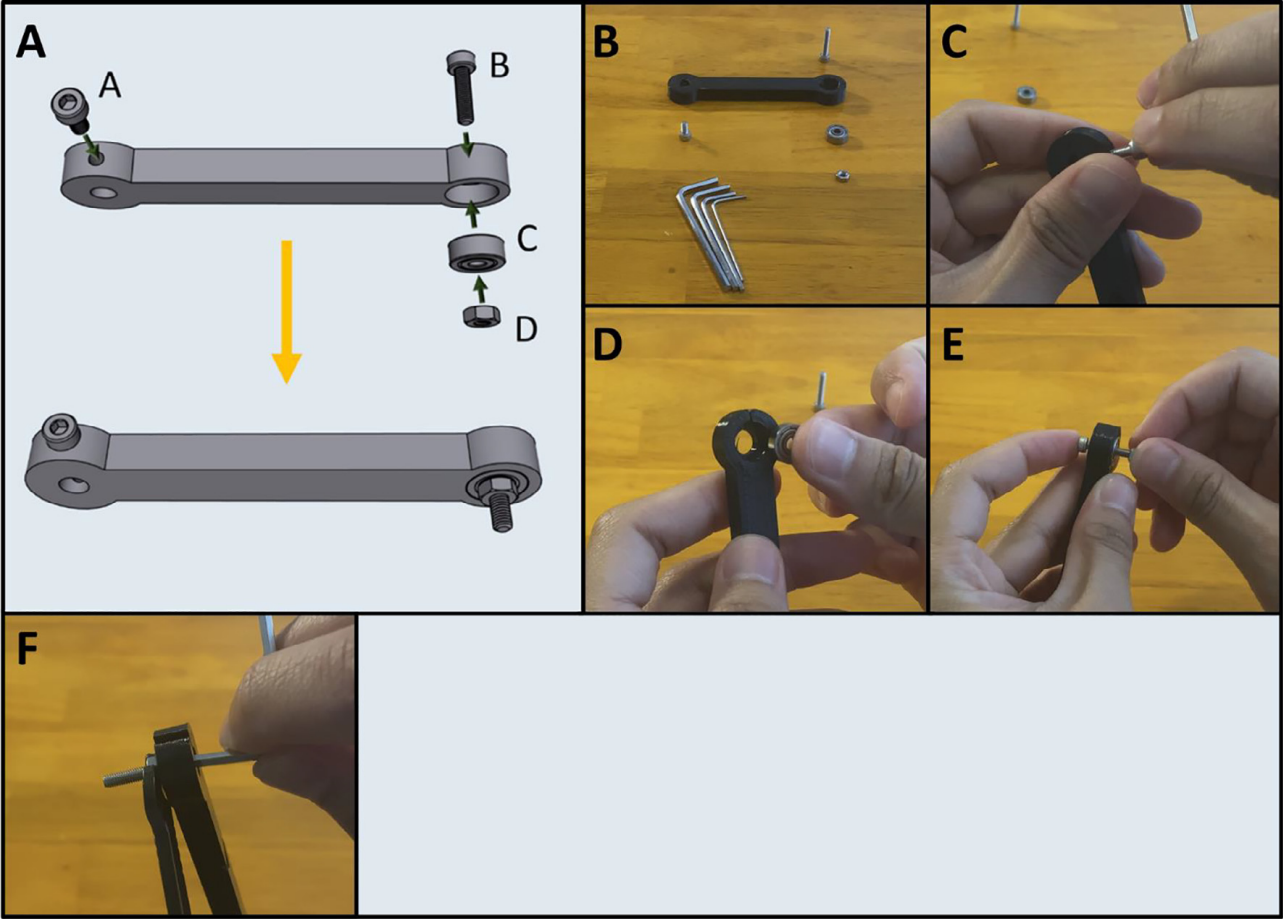
a-f: The procedure for assembling Arm One.

Fig. 15a gives an overview for the assembly of Arm One. Gather (A) an M3 × 6 screw, (B) an M3 × 12 screw, (C) a 623-2Z ball bearing and (D) an M3 hex nut. After gathering the necessary items (Fig. 15b) thread the M3 × 6 screw into the side of Arm One (Fig. 15c). Press fit the bearing into the other end of the arm (Fig. 15d) and pass the M3 × 12 screw through the bearing and thread the M3 hex nut onto the other end (Fig. 15e). Tighten the M3 × 12 screw by holding the M3 nut with a plier (Fig. 15f).

Step 2: Assembling Arm Two.

**Fig. 16:**
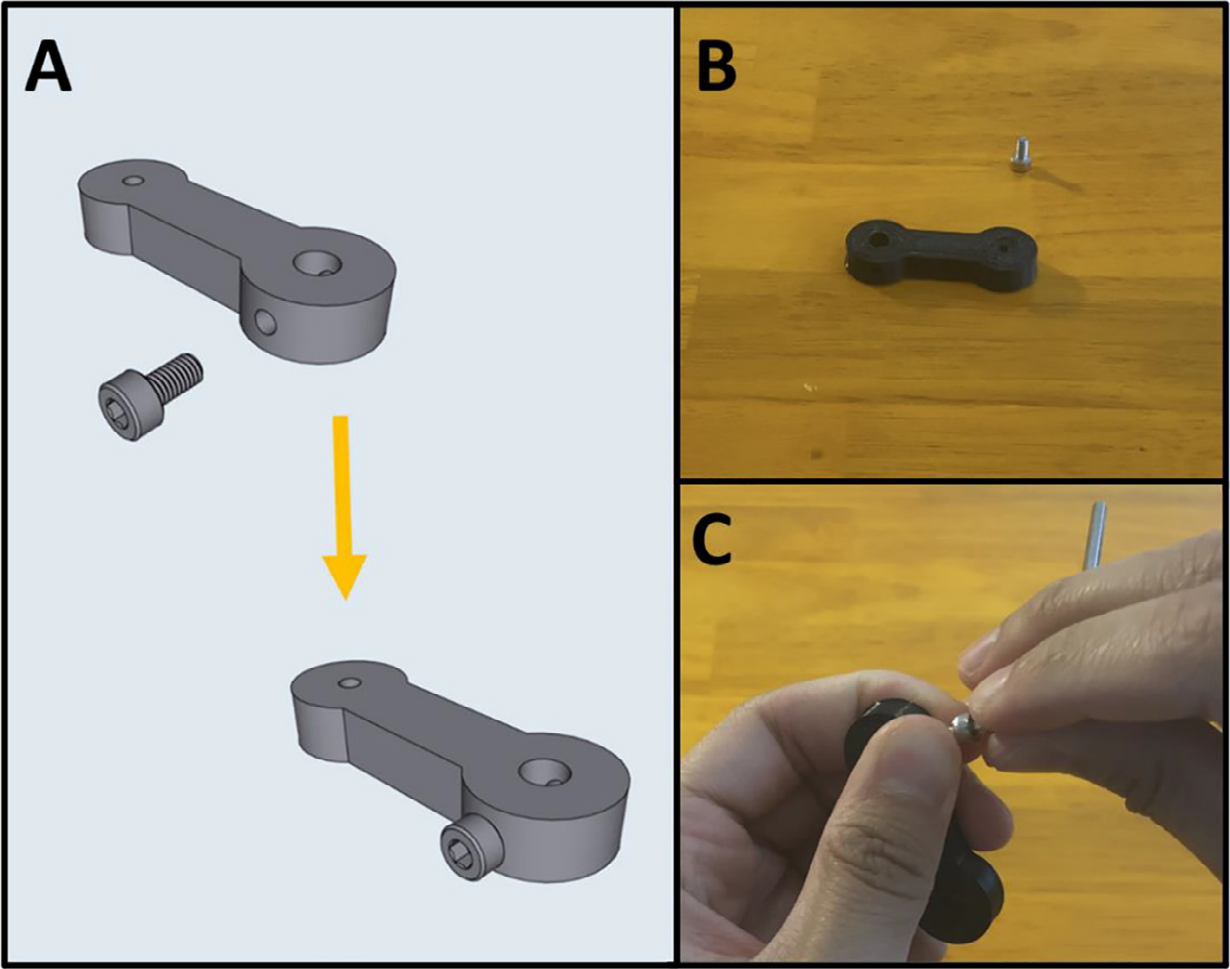
a-c: The procedure for assembling Arm Two.

Fig. 16a gives an overview for the assembly of Arm Two. Gather the 3D-printed Arm Two, and an M3 × 6 screw (Fig. 16b). Thread the M3 × 6 screw into the side of Arm Two (Fig. 16c).

Step 3: Assembling Arm Three.

**Fig. 17:**
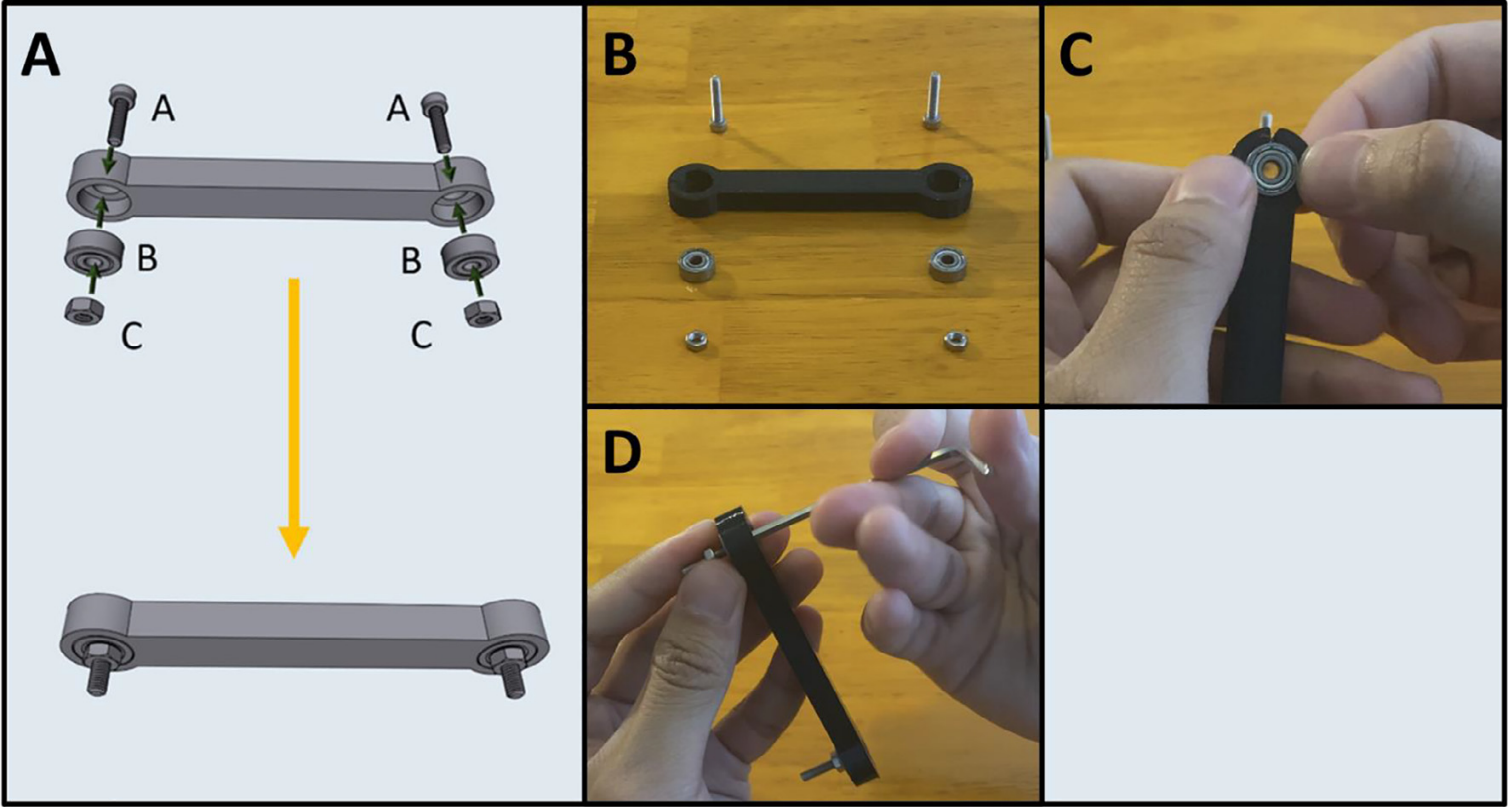
a-d: The procedure for assembling Arm Three.

Fig. 17a gives an overview for assembling Arm Three. This step requires (A) two M3x12 screws, (B) two 623-2Z ball bearings and (D) two M3 hex nuts. After gathering the required hardware and the 3D-printed Arm Three (Fig. 17b), press fit the ball bearing into Arm Three (Fig. 17c), then pass an M3x12 screw through the bearing and thread a hex nut onto the screw (Fig. 17d). Repeat for the other end.

Step 4: Connecting Arms Two and Three.

**Fig. 18:**
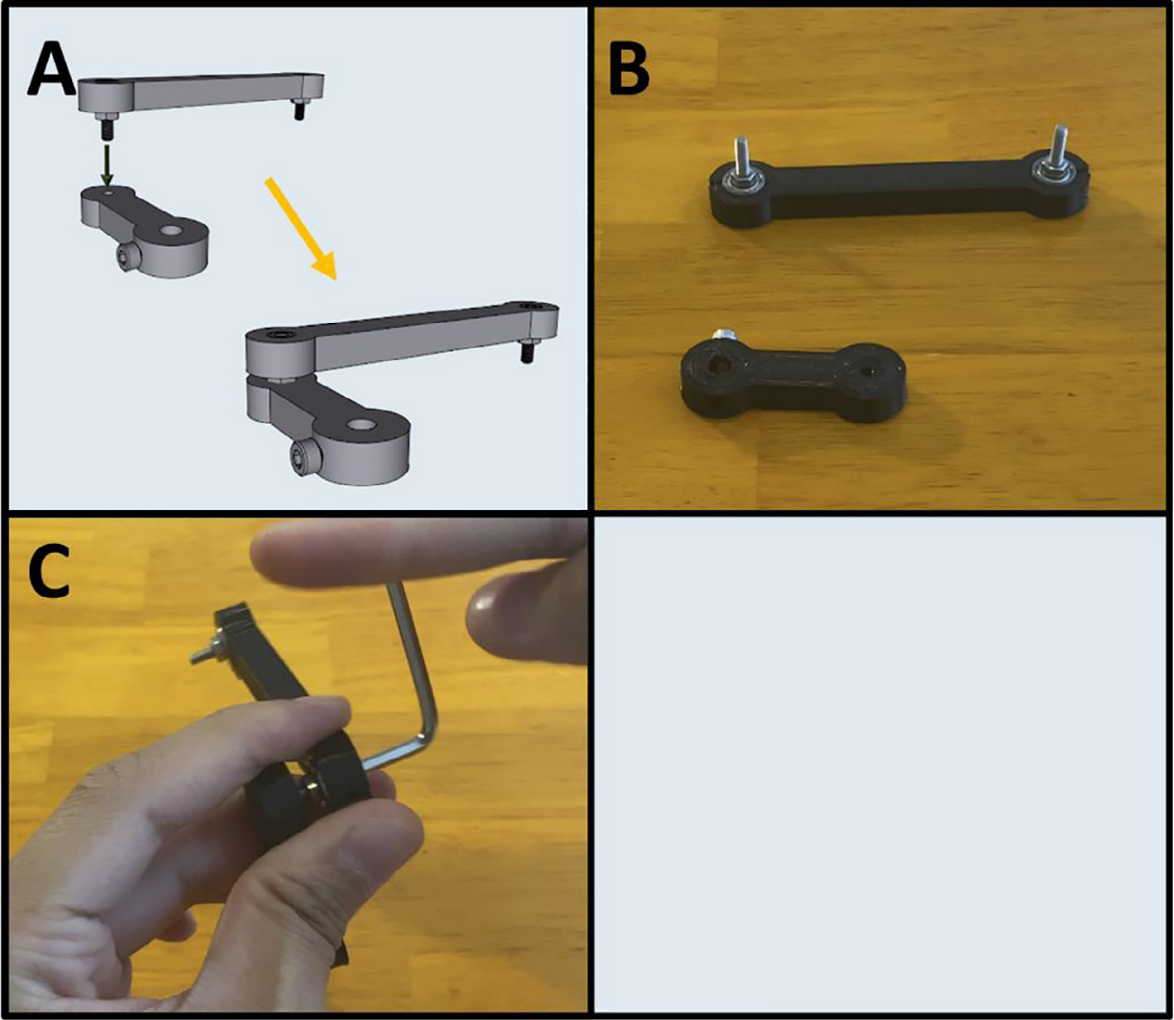
a-c: The procedure for connecting Arms Two and Three.

Fig. 18a gives an overview for the connecting of Arms Two and Three. Gather both arms (Fig. 18b). Thread the remaining length of the M3 × 12 screw of Arm Three into Arm Two (Fig. 18c). Both ends of Arm Three are identical, so it does not matter which side is attached to Arm Two. After attaching, rotate Arm Three to check for any binding. The arm should be able to rotate freely.

Step 5: Prepare the center point of Arm Four.

**Fig. 19:**
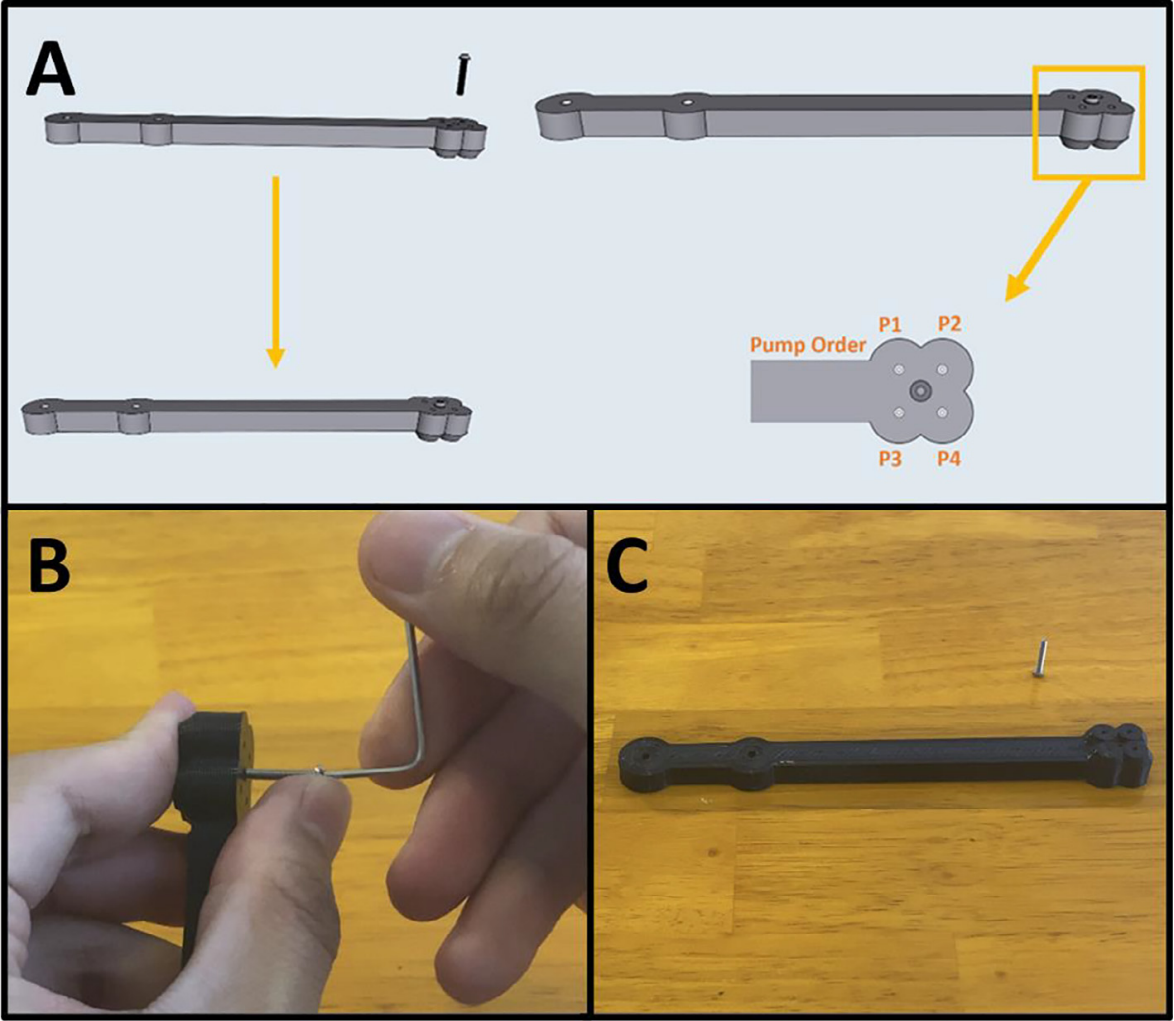
a-c: Prepping the center-point of Arm Four.

Fig. 19a gives an overview of the center-point preparation, and a diagram of the pump order on the end effector. Gather an M2x12 screw and the 3D-printed Arm Four (Fig. 19b). Thread in the M2x12 screw into the center point of Arm Four (Fig. 19c).

**Fig. 20:**
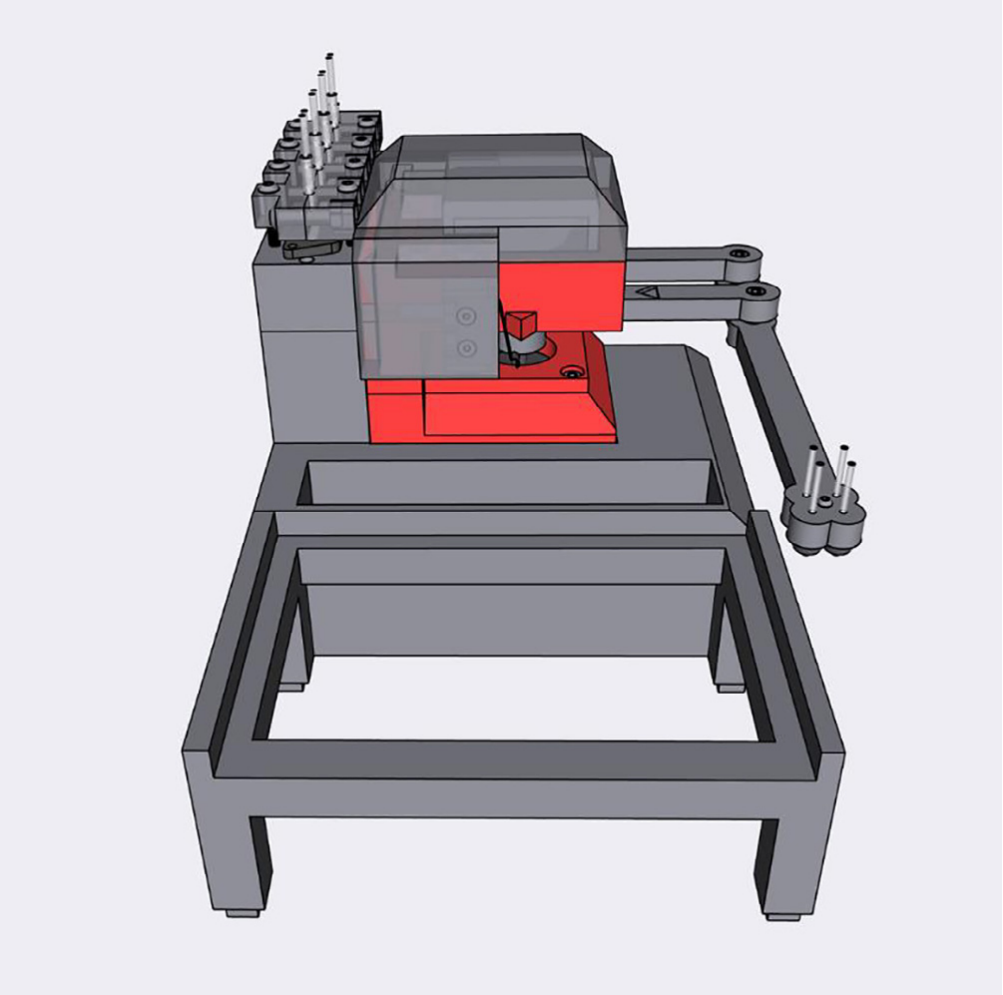
The Sidekick Motor Mount Assembly.

5D: Motor Mount Assembly. (Fig. 20)

Step 1: Mounting the Top Stepper Motor.

**Fig. 21:**
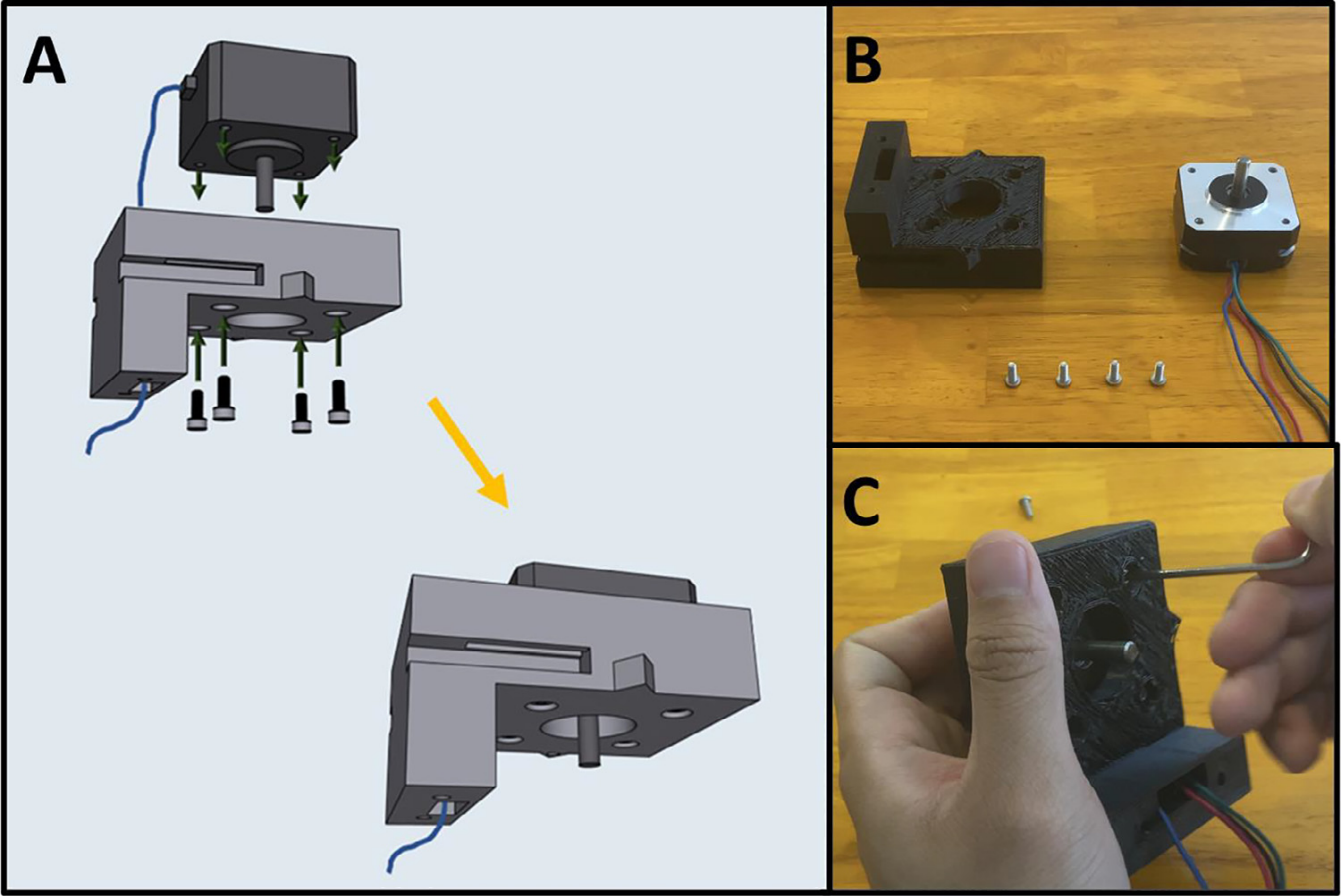
a-c: The procedure for mounting the upper stepper motor.

Fig. 21a gives an overview for mounting the upper stepper motor. Gather the 3D-printed upper motor mount, four M3x8 screws, and a prepared stepper motor (Fig. 21b). Place the stepper motor into the top mount, with the stepper motor wires facing the cable management channel. Then secure the motor in place with four M3 × 8 screws (Fig. 21c).

Step 2: Attaching Limit Switches to Switch Mounts.

**Fig. 22:**
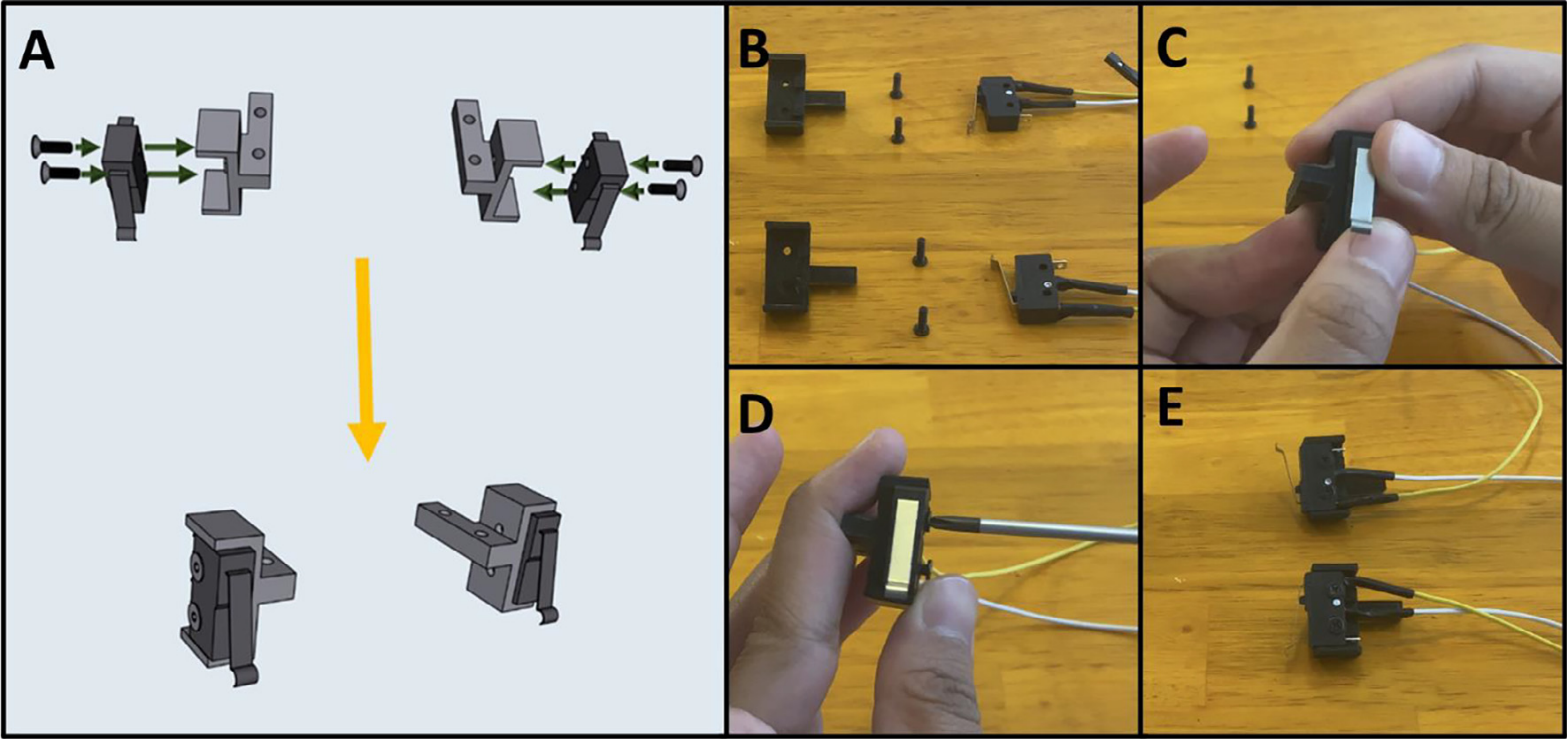
a-e: The procedure for attaching the limit switches to their mounts.

Fig. 22a gives an overview for attaching the limit switches to the Upper Motor Mount. Gather the two prepared limit switches, the two 3D-printed switch mounts, and four M2.5 × 8 screws (Fig. 22b). Press the Limit Switch onto the Limit Switch Mount (Fig. 22c) and secure them in place with two M2.5 × 8 screws (Fig. 22d). Repeat the procedure for the other limit switch (Fig. 22e).

Step 3: Attaching Limit Switch Mounts onto Top Motor Mount.

**Fig. 23:**
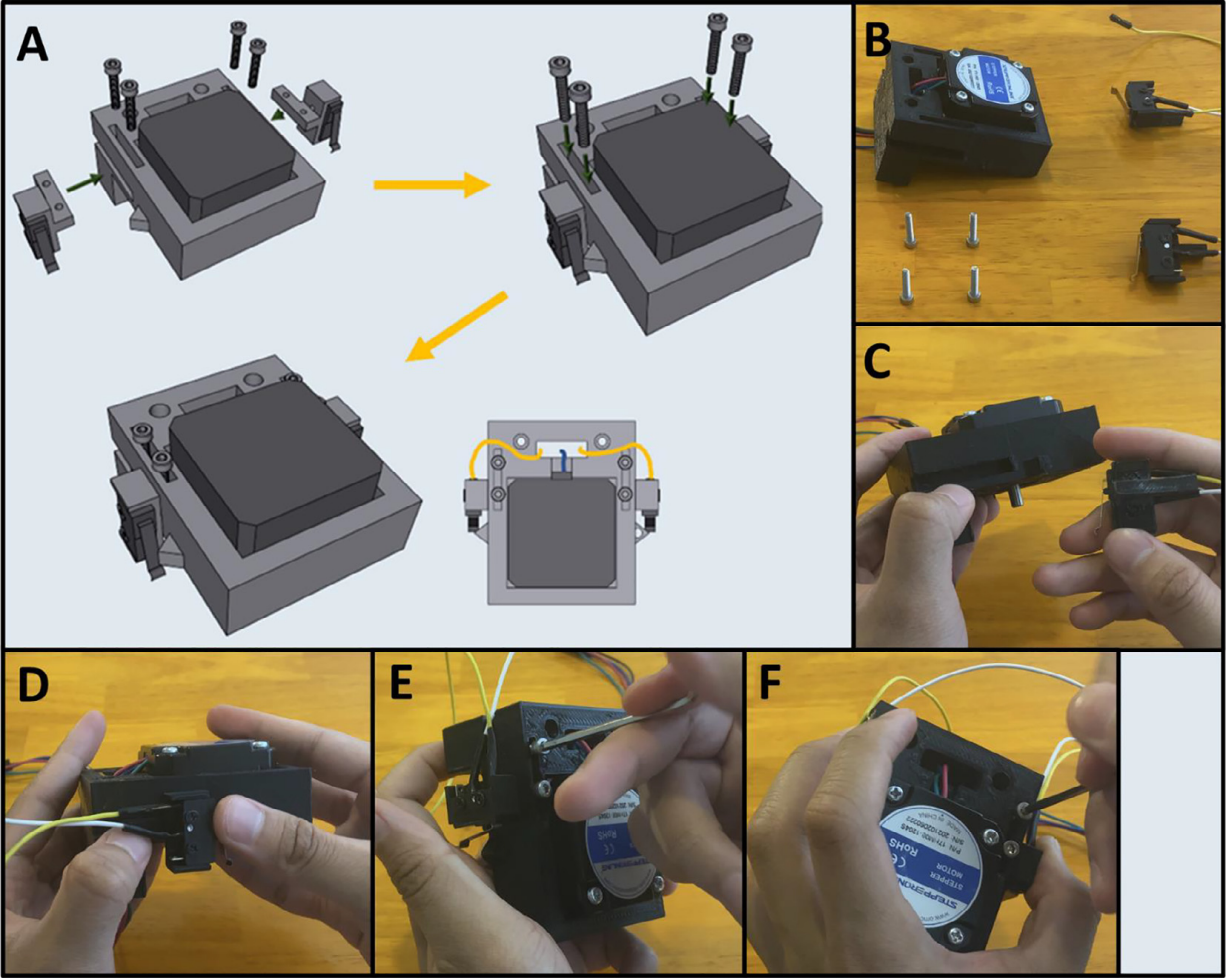
a-f: The procedure for mounting the limit switches to the Upper Motor Mount.

Fig. 23a gives an overview for attaching the limit switch assemblies to the Upper Motor Mount. Gather the Upper Mount Assembly, four M3 × 16 screws, and the two limit switch assemblies (Fig. 23b). Press the limit switch assemblies into the notches of the Upper Motor Mount (Fig. 23c-d). Then screw two M3 × 16 screws onto each of the limit switch mounts to secure them to the Upper Motor Mount (Fig. 23e-f). Pass the limit switch cables through the cable management channel indicated by the yellow-colored lines indicated in Fig. 23a.

Step 4: Attaching Arm One onto the Upper Motor Assembly.

**Fig. 24:**
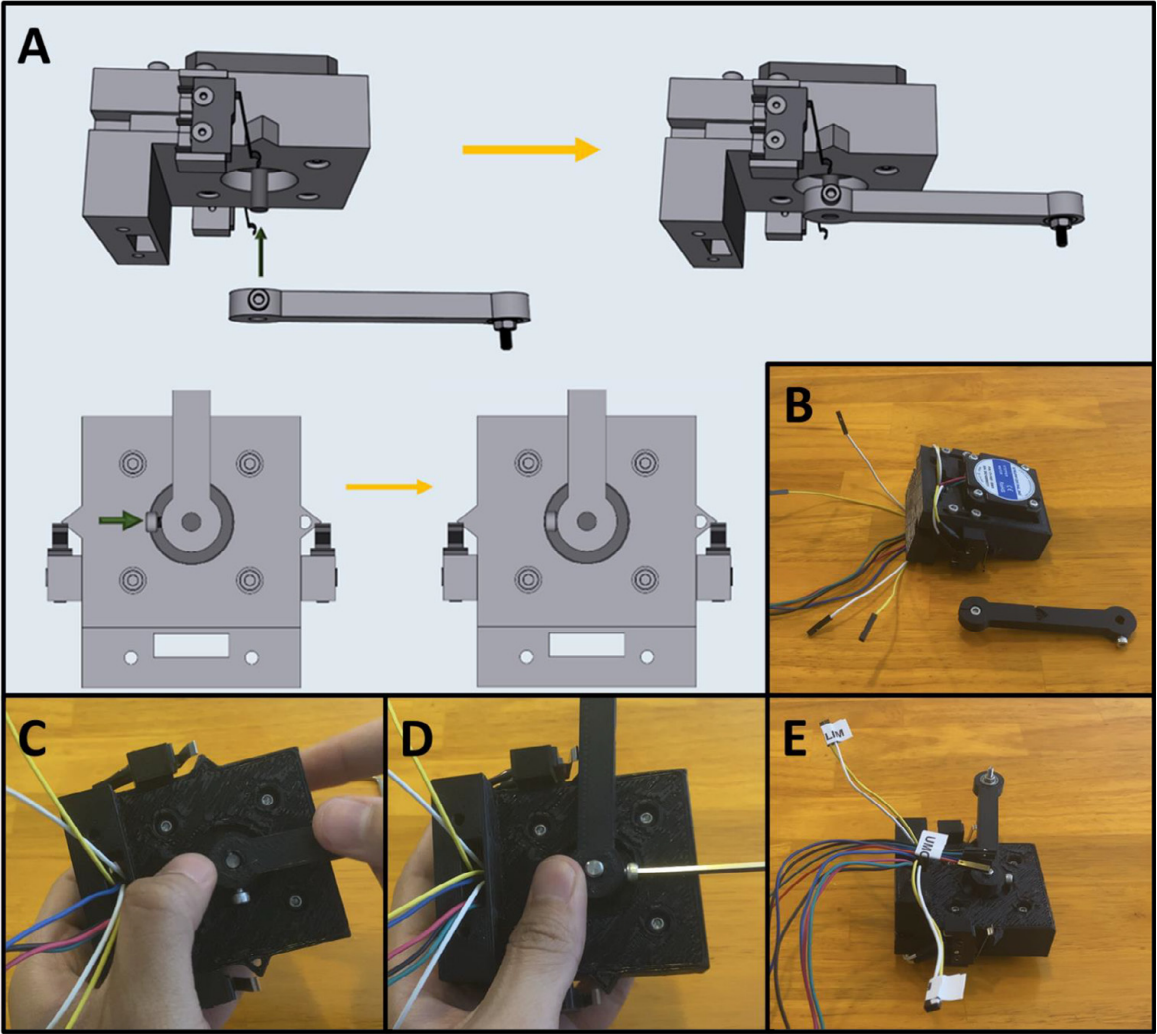
a-e: The procedure for attaching Arm One onto the Upper Motor Assembly.

Fig. 24a gives an overview of attaching Arm One onto the Upper Motor Assembly. Gather the Upper Motor Assembly and Arm One (Fig. 24b). Press fit Arm One onto the shaft of the Upper Stepper motor as indicated (Fig. 24c). The arm should be able to rotate and engage the limit switches, without scraping against the top of the motor mount. Once satisfied with the position of Arm One, tighten the M3 × 6 screw on Arm One to secure it to the motor shaft (Fig. 24d). Label the end of the cables of the electrical components with a unique tag to assist in wiring (Fig. 24e).

Step 5: Setting Home Position for Arm One.

**Fig. 25:**
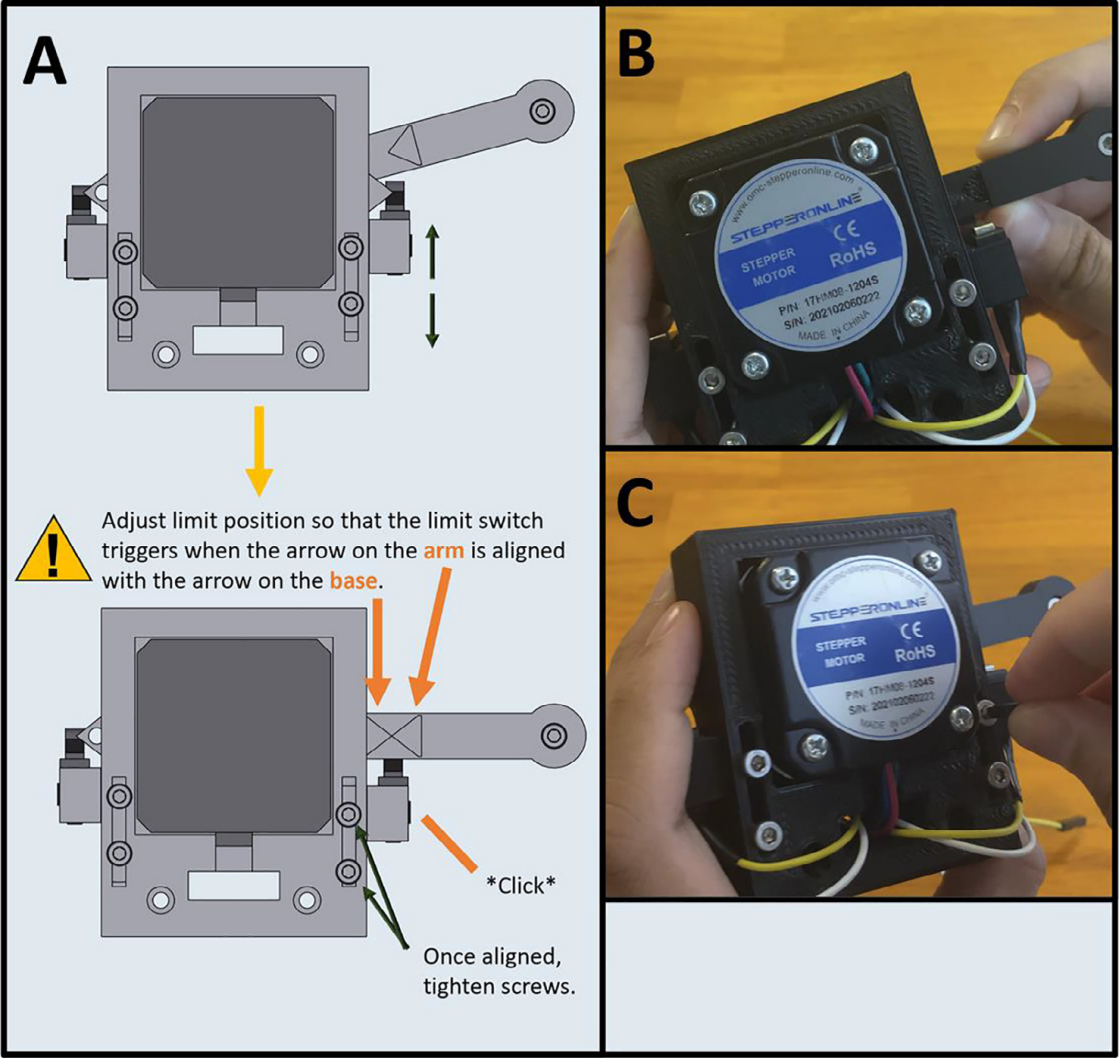
a-c: The procedure for setting the home position for the front limit switch.

Fig. 25a gives an overview for setting the limit switch position. Adjust the Front Limit switch mount, so that the limit switch is engaged once Arm One is at the correct zero position, indicated by the alignment of the triangle on the Upper Motor Mount (Fig. 25b). Once the Front Limit Switch Mount is in the correct position, tighten the two M3x16 screws (Fig. 25c).

Step 6: Mounting the Lower Stepper Motor.

**Fig. 26:**
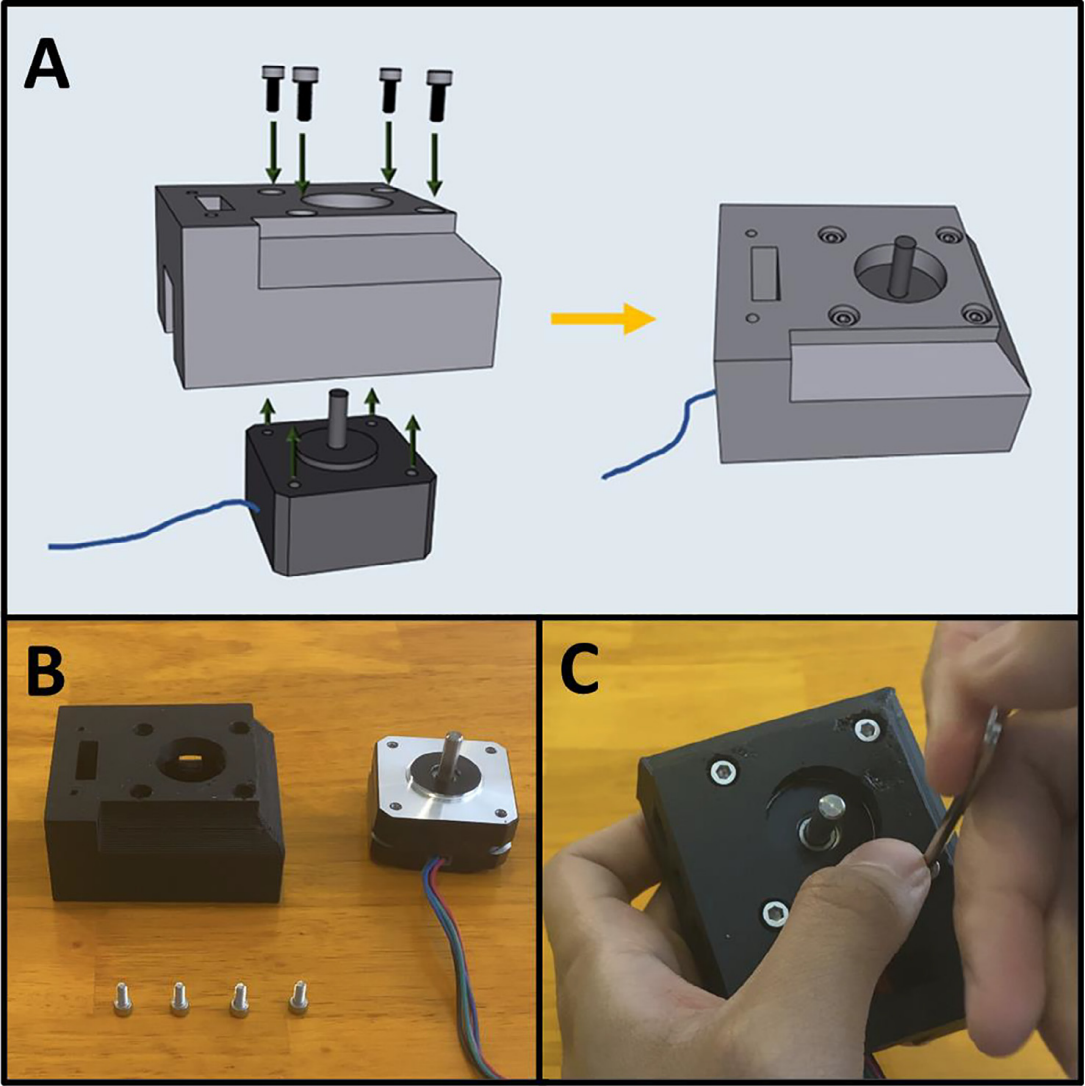
a-c: The procedure for mounting the lower stepper motor.

Fig. 26a gives an overview of the mounting procedure. Gather the remaining stepper motor, four M3 × 8 screws, and the 3D-printed Lower Motor Mount (Fig. 26b). Place the stepper motor into the lower mount, with the stepper motor wires facing the cable management channel, then secure the motor in place with four M3 × 8 screws (Fig. 26c).

Step 7: Attaching Arm Two and Three to the Lower Motor Assembly.

**Fig. 27:**
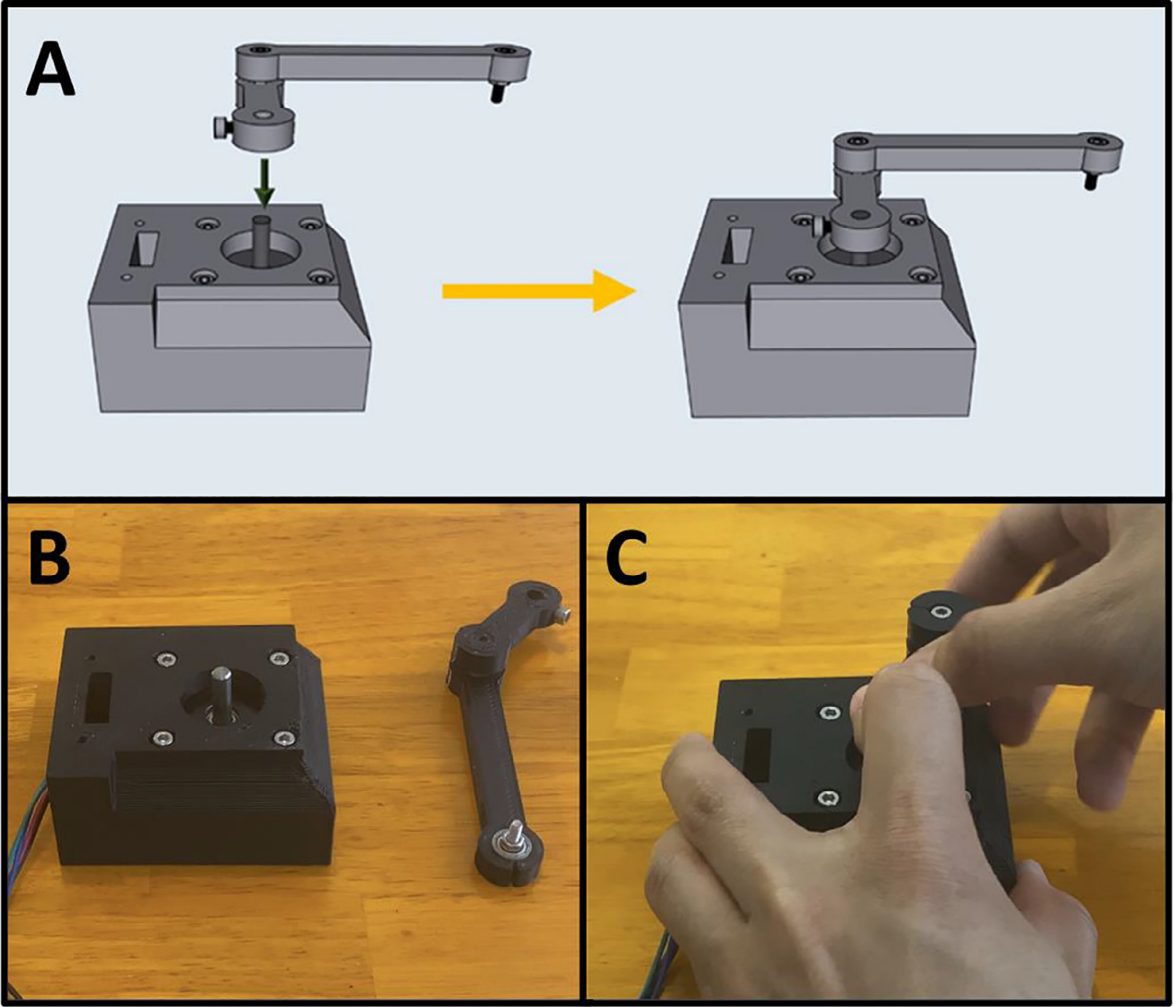
a-c: The procedure for attaching Arm Two and Three to the lower motor mount.

Fig. 27a gives an overview of the mounting process. Gather the lower motor mount, and the Arm Two and Three assembly (Fig. 27b). Press fit Arm Two onto the stepper motor shaft with Arm Three facing upwards (Fig. 27c). The arms should be able to rotate freely without catching on anything.

Step 8: Tightening Arm Two on the Lower Stepper Motor.

**Fig. 28:**
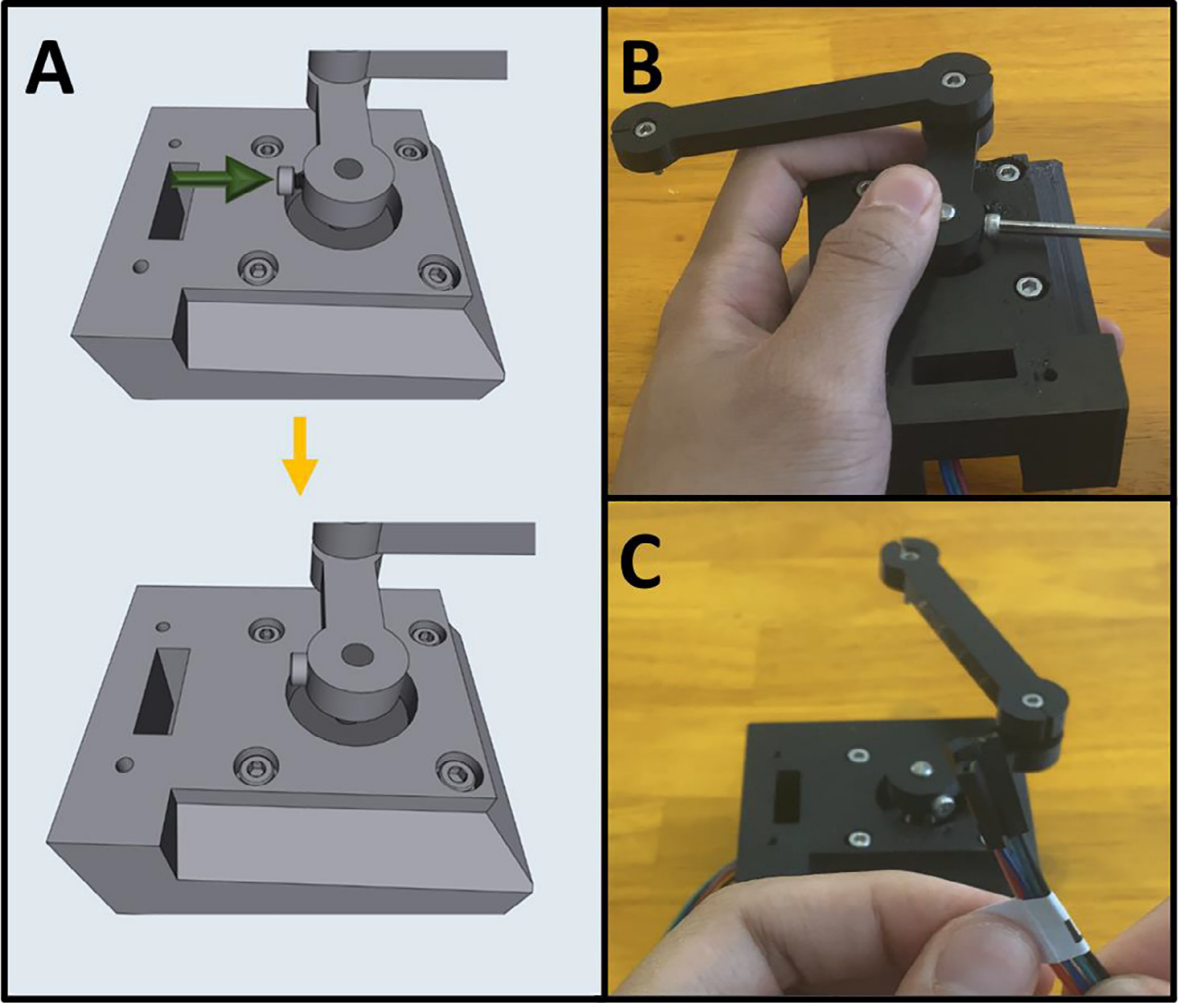
a-c: The procedure for tightening the arm assembly onto the lower stepper motor.

Fig. 28a gives an overview of the tightening procedure. Once satisfied with the position of Arm Two, tighten the M3 × 6 screw on Arm Two to secure it to the motor shaft (Fig. 28b). Note that there are two screw holes on Arm Two. Only one M3 × 6 screw is required to tighten the leg onto the motor shaft, use whichever one is easier. If your motor shaft has a flat face, secure the M3 × 6 screw onto it. Label the lower stepper motor cables (Fig. 28c).

Step 9: Attaching the Upper Motor Assembly to the Lower Motor Assembly.

**Fig. 29:**
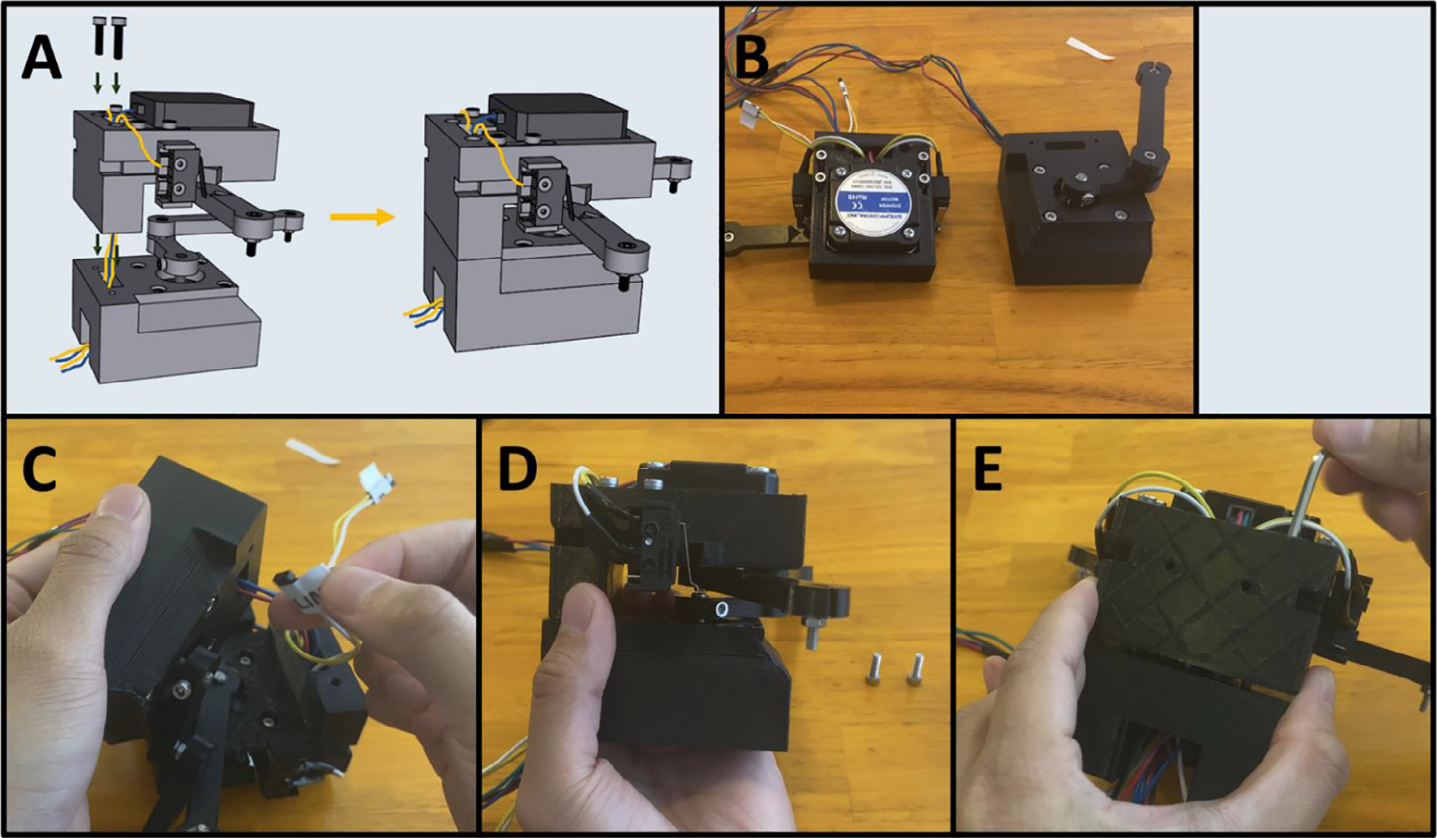
a-e: The procedure for joining the Upper and Lower Motor Assemblies.

Fig. 29a gives an overview for joining the Upper and Lower Motor Assemblies. Gather the two mount assemblies (Fig. 29b). Thread the cables from electrical components on the Upper Motor Assembly through the cable management tunnel of the Lower Motor Assembly (Fig. 29c-d). Then secure the two together with two M3 × 12 screws (Fig. 29e).

Step 10: Setting Home Position for Arm Two.

**Fig. 30:**
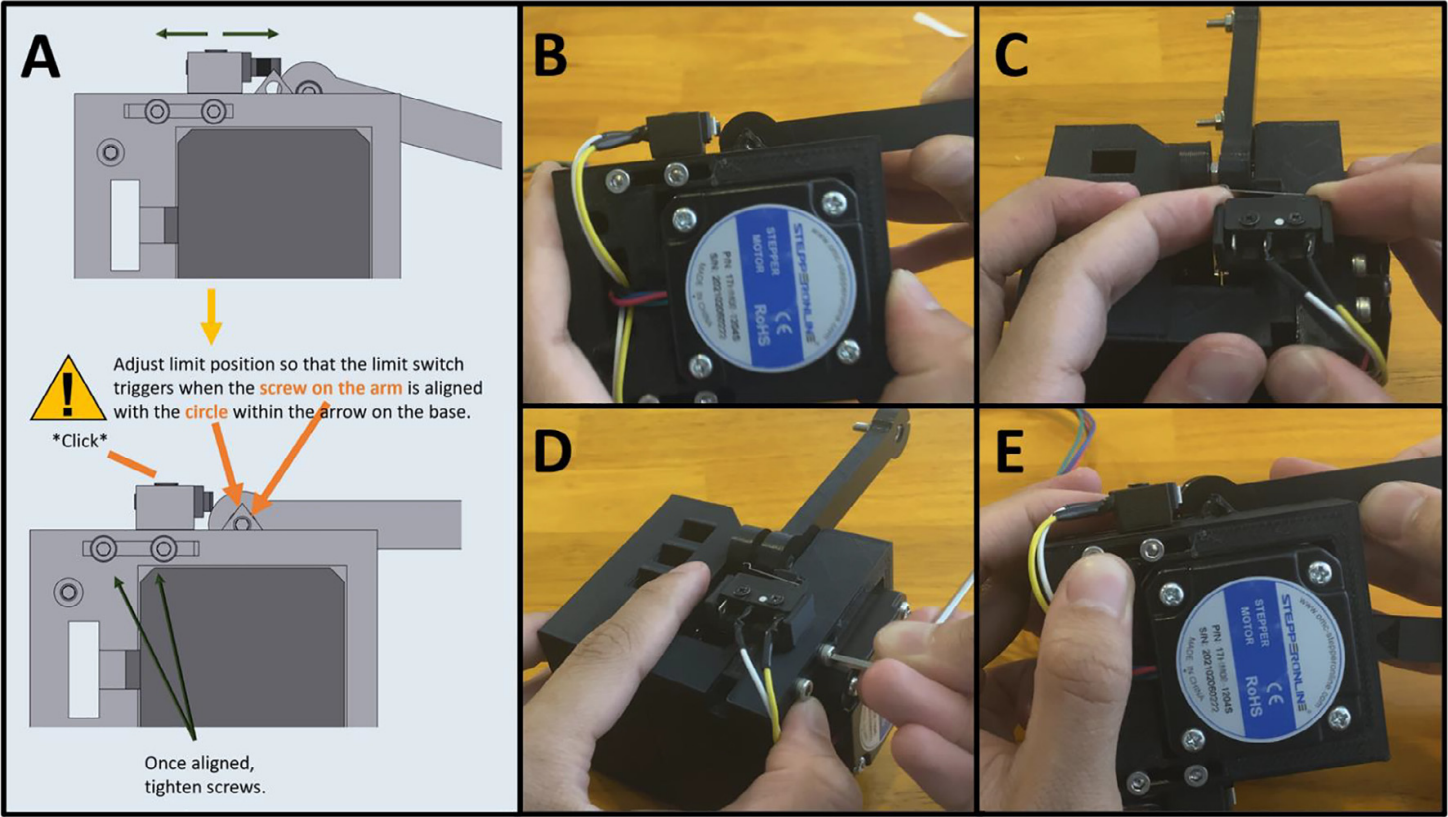
a-e: The procedure for setting the home position for Arm Two.

Fig. 30a gives an overview for setting the home position for Arm Two. Adjust the Rear Limit Switch Mount, so that the limit switch is engaged once Arm Two is at the correct zero position, indicated by the alignment of the screw on the arm aligning with the inscribed circle on the Upper Motor Mount (Fig. 30b). If the limit switch is not in the correct position, loosen the M3 screws, and then push the limit switch into the proper position (Fig. 30c). Once the Rear Limit Switch Mount is in the correct position, tighten the two M3 × 16 screws (Fig. 30d). Double check to ensure that the limit switch is triggered when the screw on the arm is aligned with the arrow on Upper Mount Assembly (Fig. 30e).

Step 11: Setting the gap between Arm One and Arm Two.

**Fig. 31:**
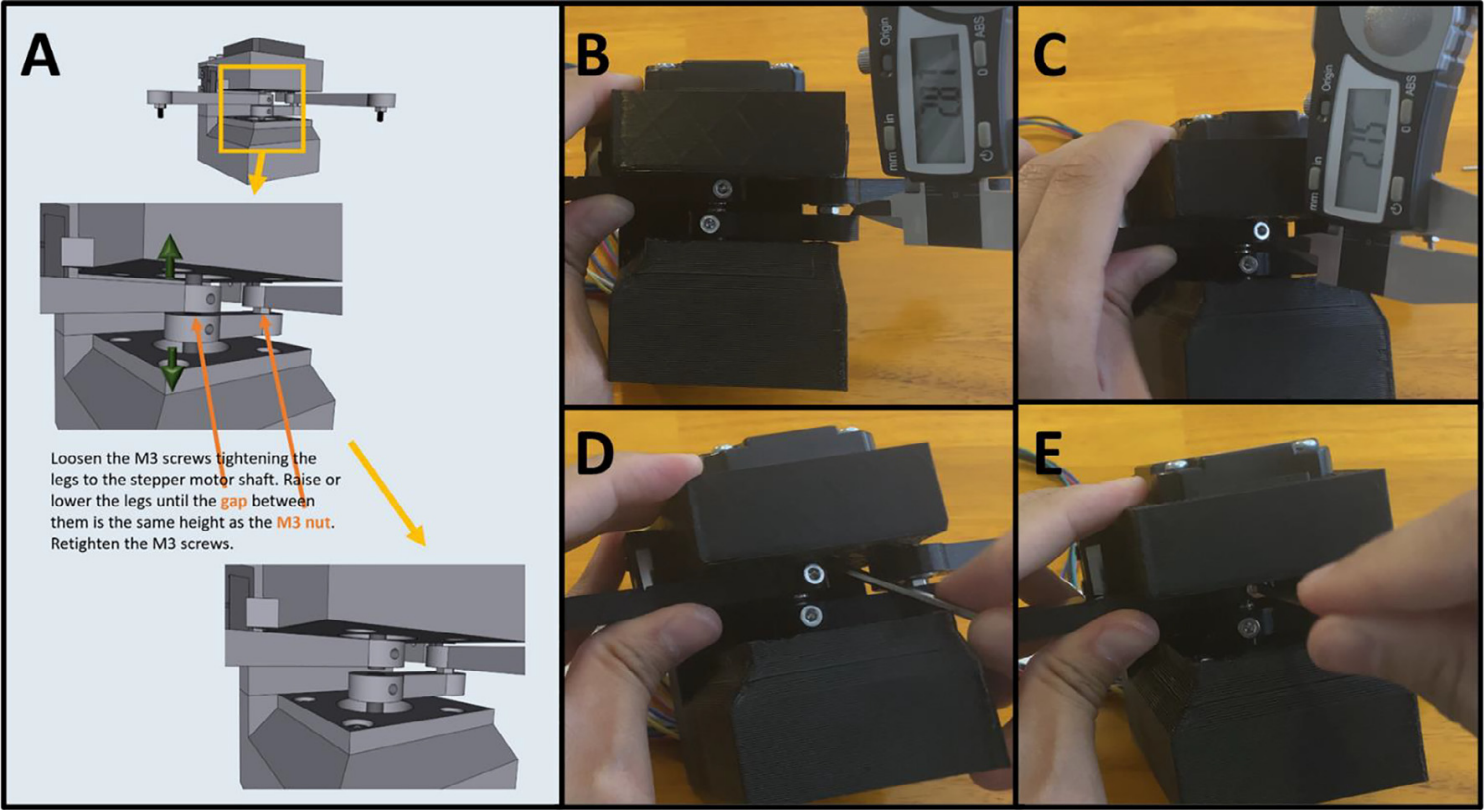
a-e: The procedure for setting the gap between Arm One and Two.

Fig. 31a gives an overview for adjusting the gap between Arm One and Arm Two, so that the distance between the two is roughly equal to the height of the M3 nut between Arm Two and Arm Three. The distance between Arm One and Arm Two should be about the width of an M3 nut (∼3 mm). A caliper is pictured (Fig. 31b-c) but any measuring tool that lets you estimate the distance will work. To adjust the arms, loosen the M3 screws holding them against motor shafts and slide a flat head screwdriver or hex key into the gap (Fig. 31d). When gapping the two arms, be sure that neither arm brushes against the upper or lower motor mount. Once satisfied with the gap, retighten the M3 screws to secure the arms (Fig. 31e).

Step 12: Attaching Arm Four.

**Fig. 32:**
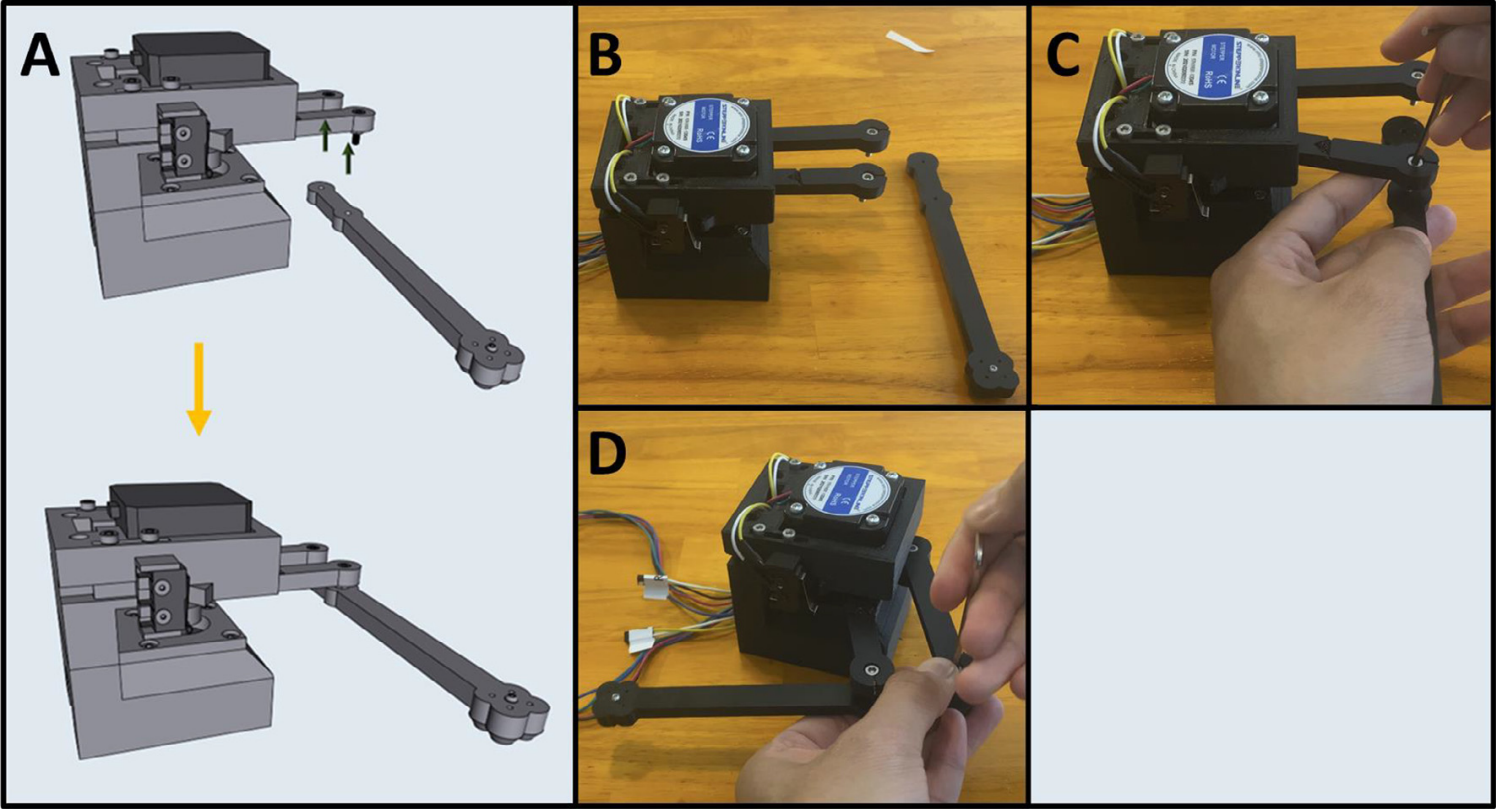
a-d: The procedure for attaching Arm Four to the motor assembly.

Fig. 32a shows an overview of attaching Arm Four to Arms One and Three to complete the armature assembly. Gather the motor mount assembly and Arm Four (Fig. 32b). Position Arm Four with the nozzle cones facing down and thread the M3 screws from Arms One and Three into Arm Four (Fig. 32c-d).

**Fig. 33:**
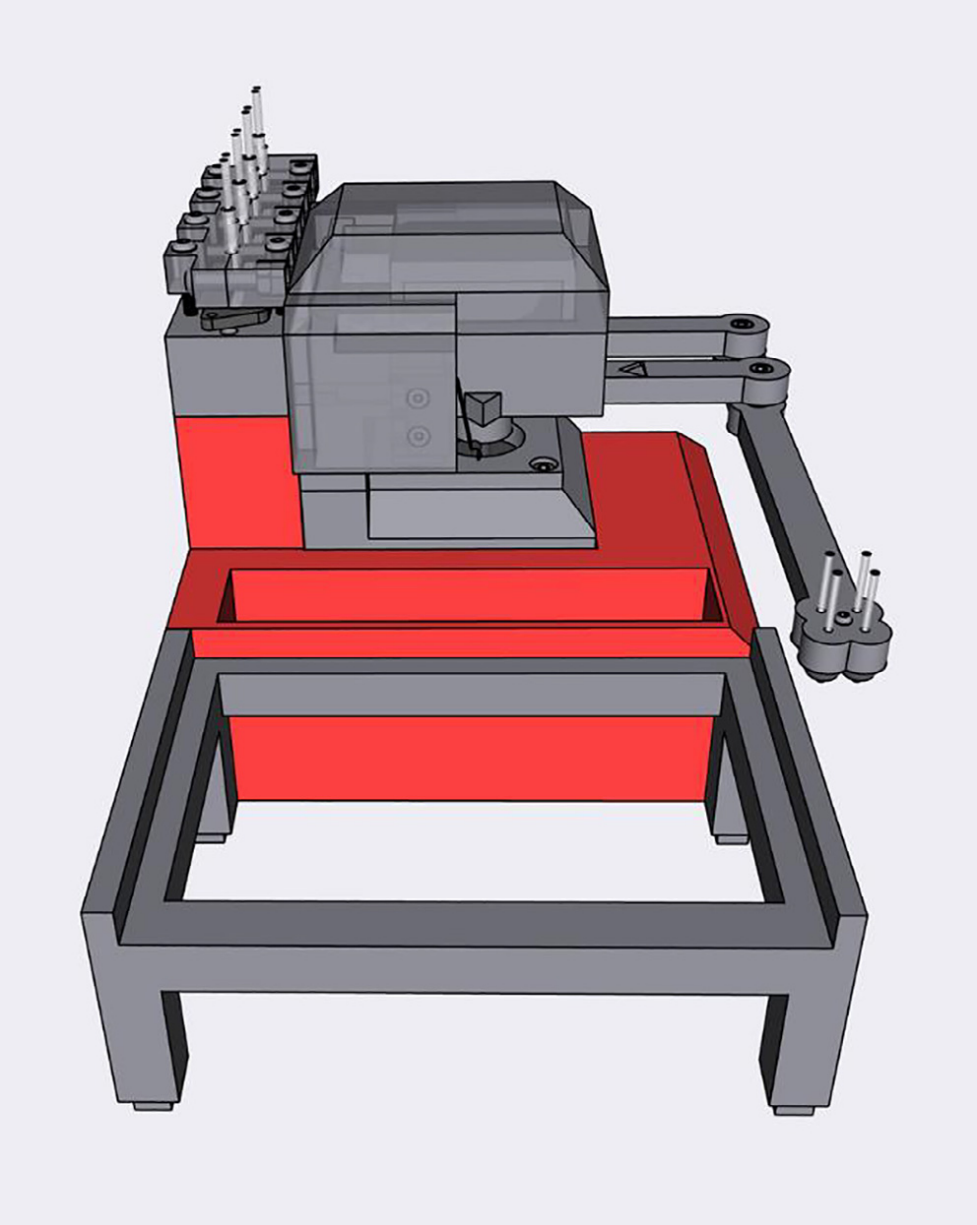
The Sidekick Base Assembly.

5E: Base Assembly. (Fig. 33)

Step 1: Purge Button Assembly

**Fig. 34:**
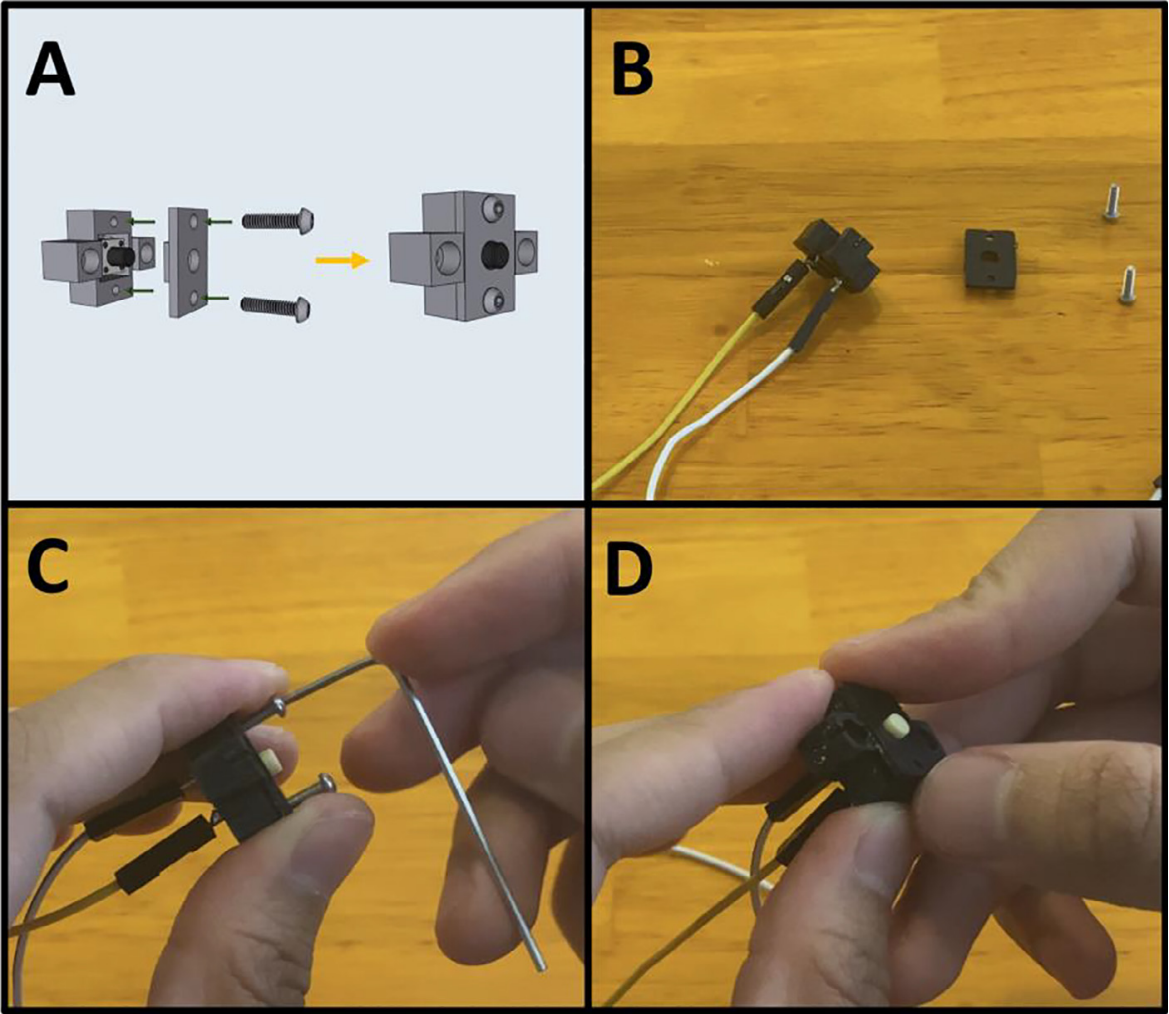
a-d: The procedure for finishing the Purge Button Assembly.

Fig. 34a gives an overview of the Purge Button Assembly. Gather the button and housing wired in 5B, Step 4, as well as the Button Housing front, and two M2 × 8 screws (Fig. 34b). Place the Button Housing front over the housing and secure it with two M2 × 8 screws (Fig. 34c). The finished assembly is shown in Fig. 34d.

Step 2: Joining the Button Assembly to the Base.

**Fig. 35:**
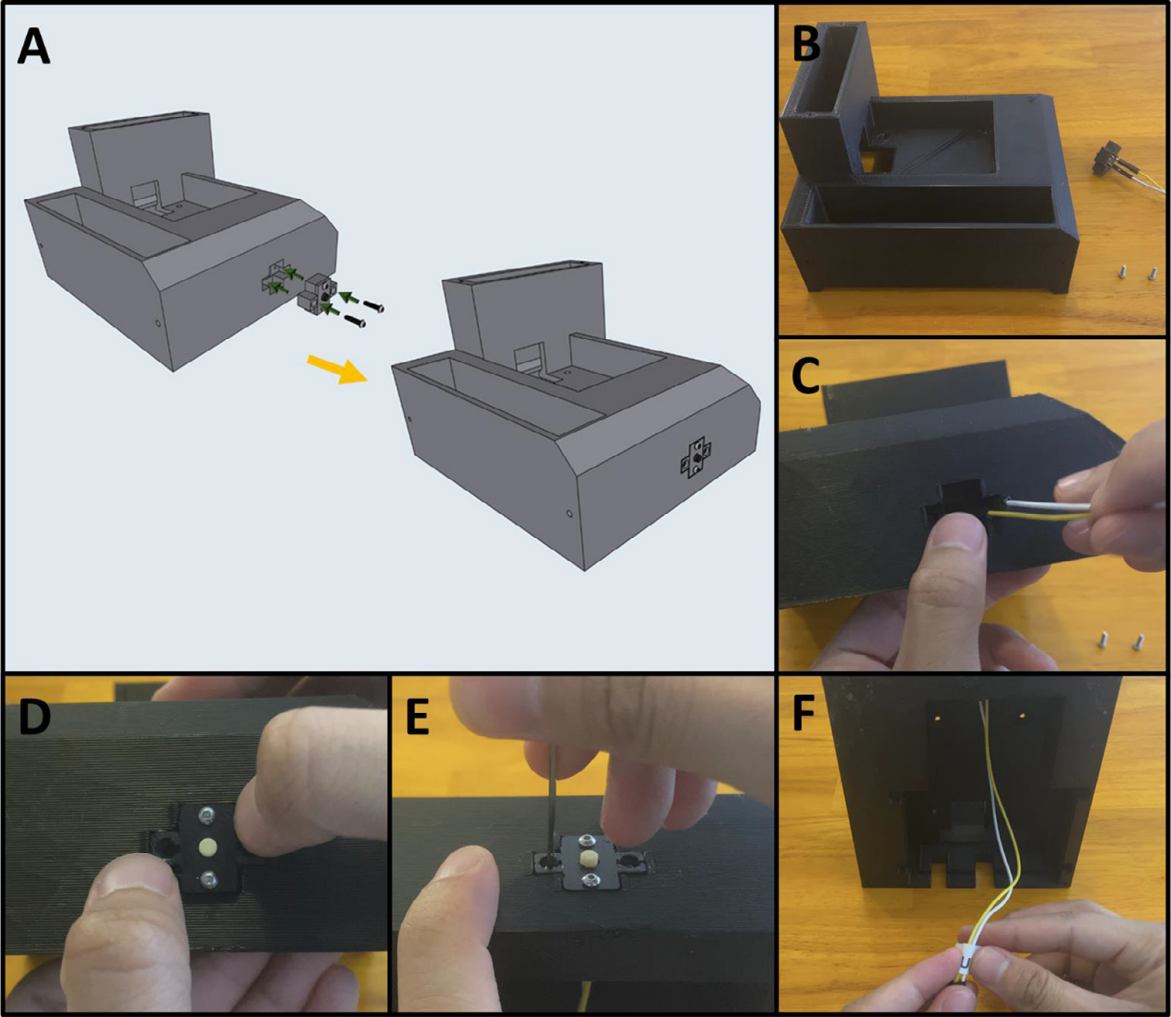
a-f: The procedure for attaching the Purge Button Assembly to the base.

Fig. 35a gives an overview for joining the Purge Button Assembly to the base. Gather the 3D-printed base, the Purge Button Assembly, and two M2 × 8 screws (Fig. 35b). Thread the button wires in the cable management slot in the base (Fig. 35c). Then press fit the Button Assembly into the base (Fig. 35d) and secure the assembly with two M2 × 8 screws (Fig. 35e). Label the purge button wires (Fig. 35f).

**Fig. 36:**
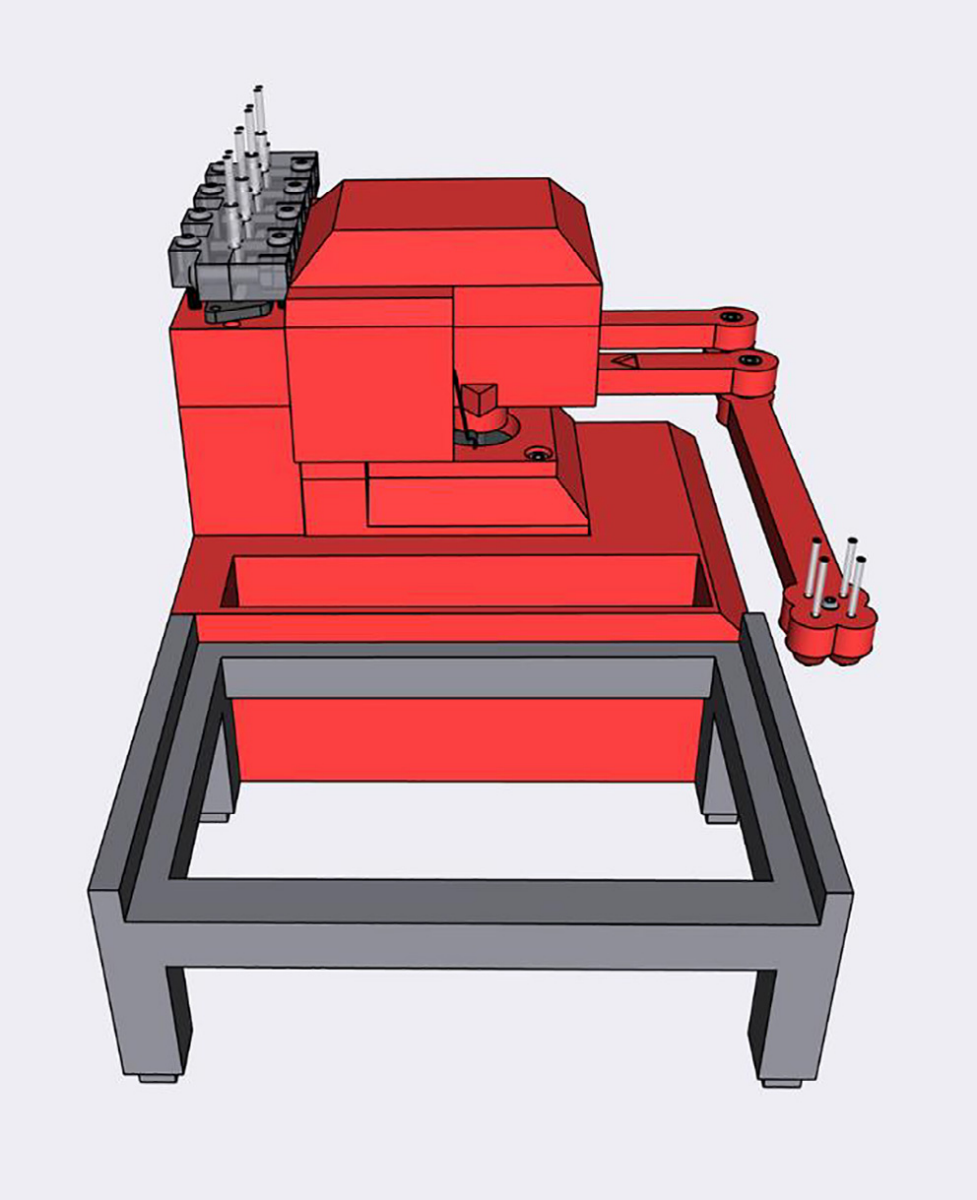
Final Assembly of the Sidekick base.

5F: Base, Final Assembly. (Fig. 36)

Step 3: Securing Pumps to Pump Mounts.

**Fig. 37:**
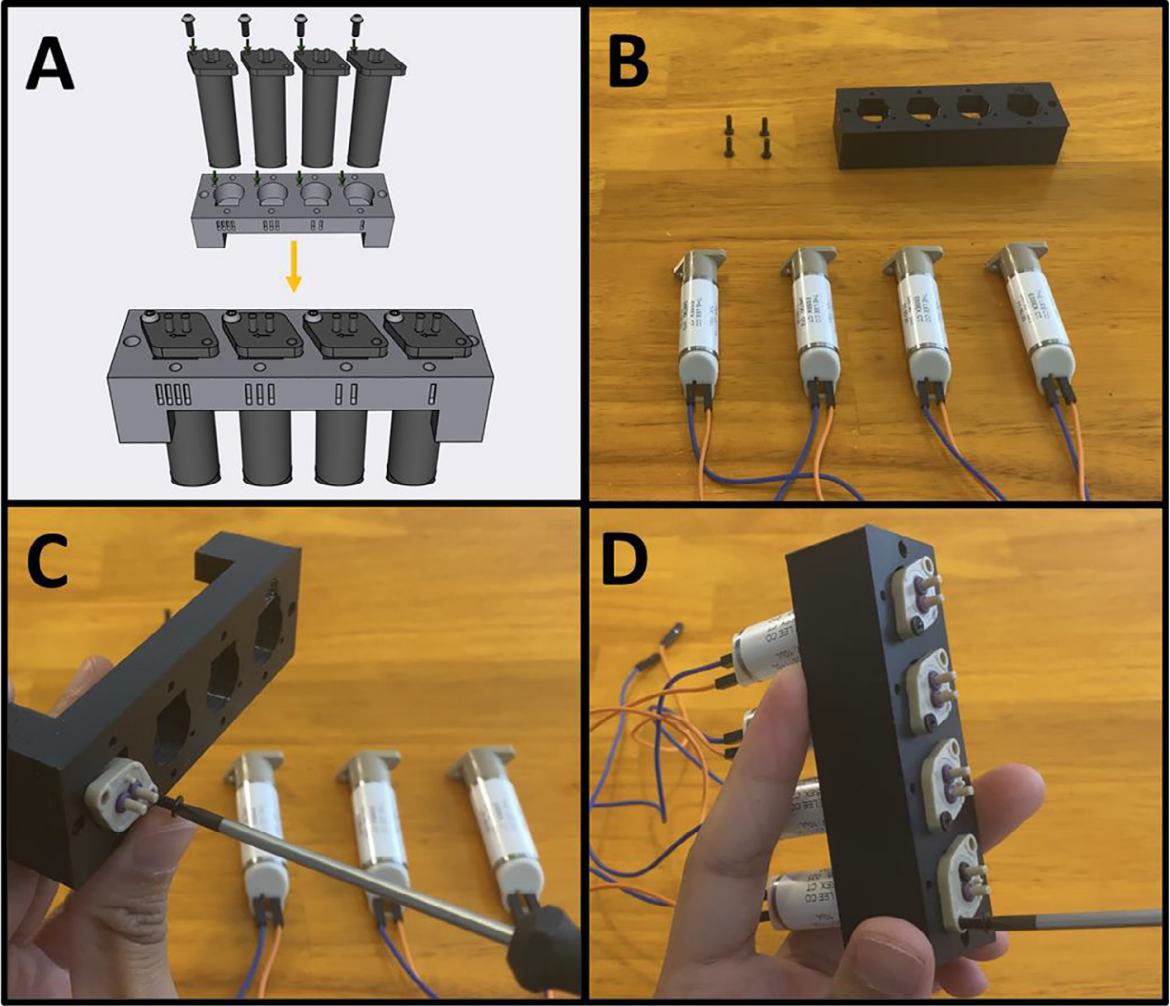
a-d: The procedure for mounting the Pumps to the Pump Mount.

Fig. 37a gives an overview for mounting the pumps. Gather four M2.5 × 6 screws, the Pump Mount, and the four pumps (Fig. 37b). Mount and secure the pumps to the Pump Mount with M2.5 × 6 screws (Fig. 37c-d). Note that only one screw is needed per pump. This allows the pumps to pivot into alignment when the adapter clamp is installed.

Step 4: Attaching the Pump Assembly to the Base.

**Fig. 38:**
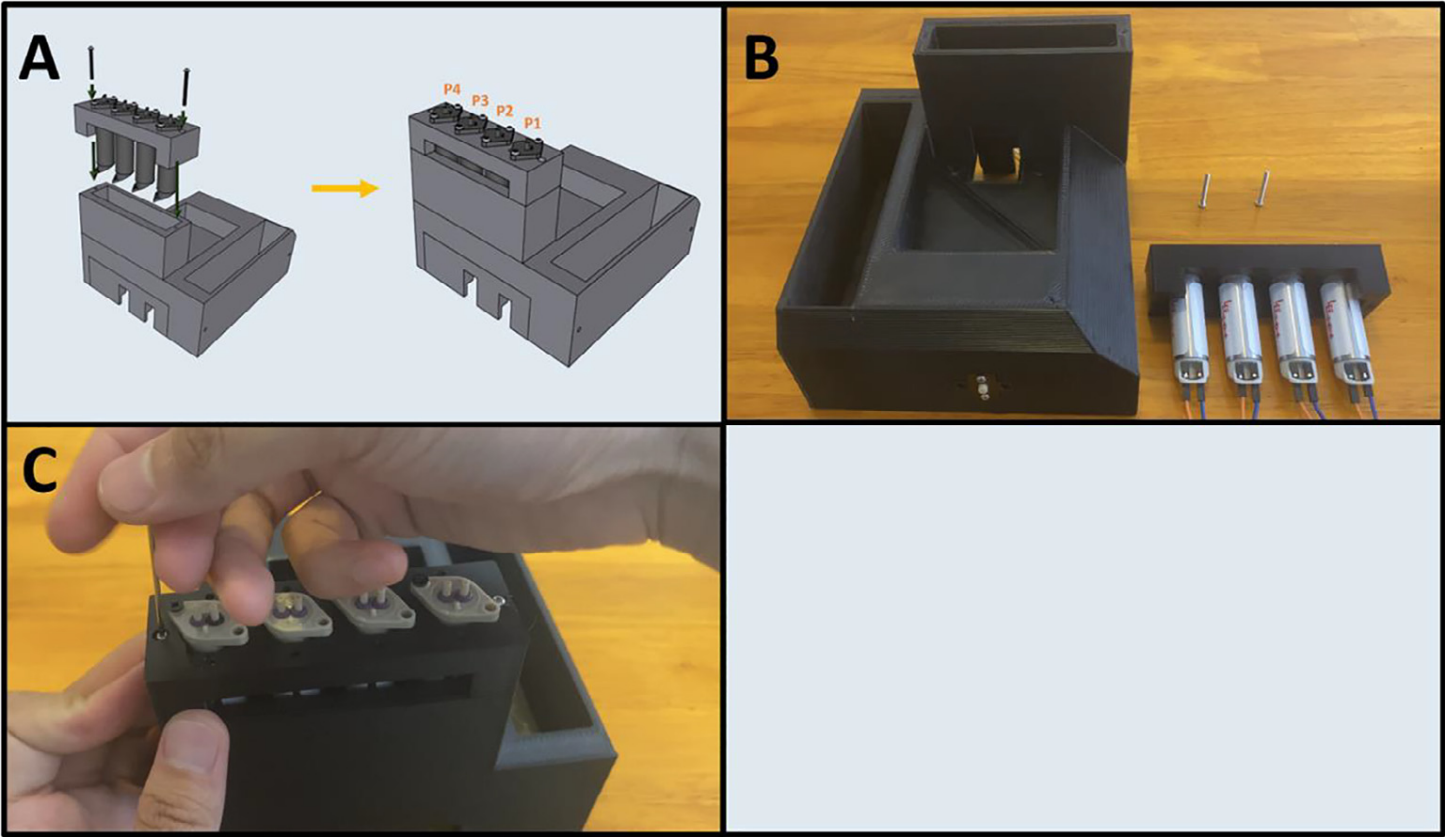
a-c: The procedure for mounting the Pump Assembly to the Base.

Fig. 38a gives an overview of the mounting process. Gather two M2 × 20 screws, the Pump Assembly, and the Base (Fig. 38b). Mount the Pump Assembly to the Base using two M2 × 20 screws (Fig. 38c).

Step 5: Attach the Motor/Armature Assembly to the Base.

**Fig. 39:**
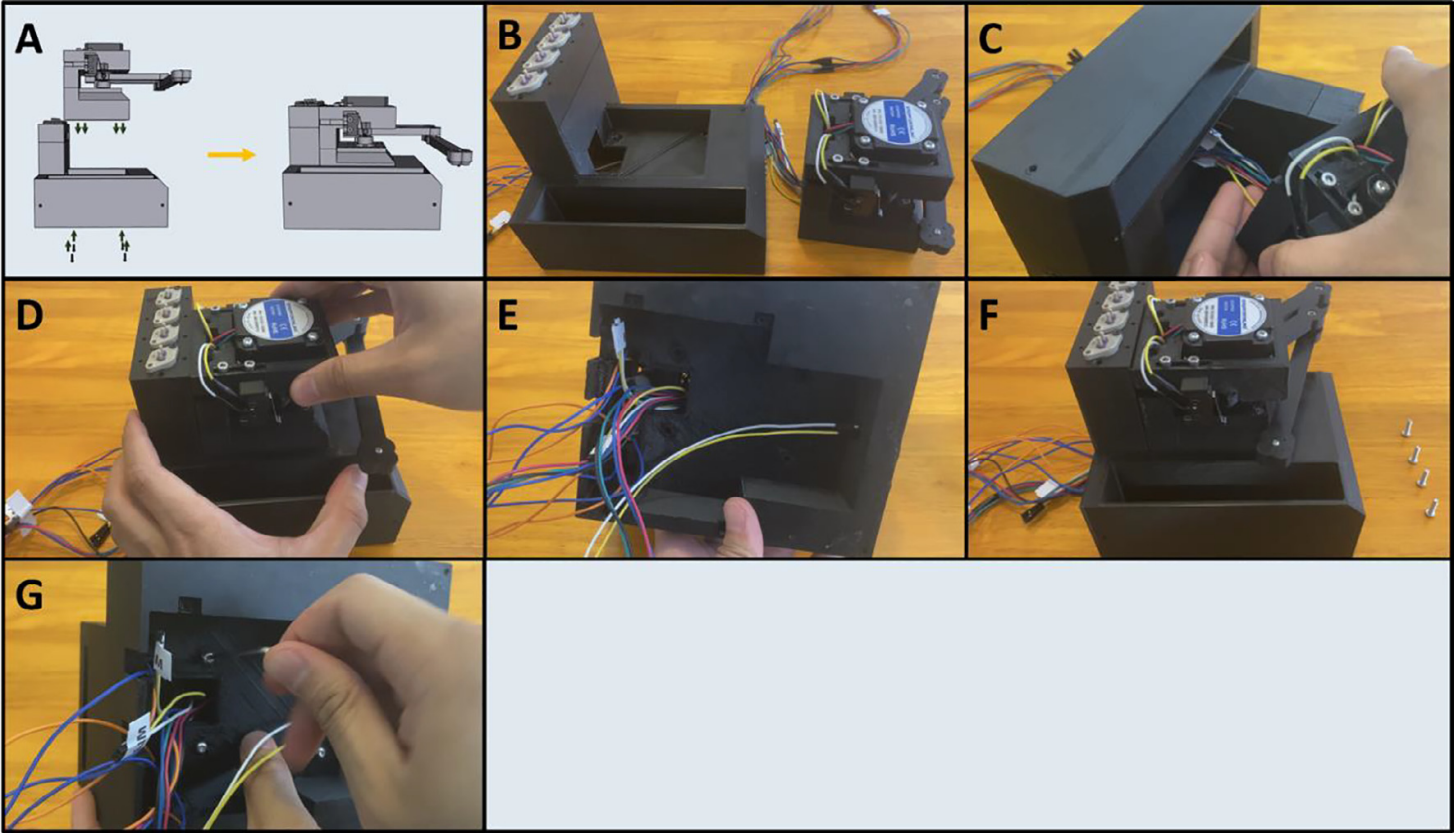
a-g: The procedure for mounting the Motor Assembly to the base.

Fig. 39a gives an overview of the mounting procedure. Gather the Base and the Motor Assembly (Fig. 39b). Thread the cables from the Motor Assembly into the gap in the Base (Fig. 39c), then seat the entire assembly into the Base (Fig. 39d). The cables should all be routed to the underside of the Base (Fig. 39e). Gather four M3 × 8 screws (Fig. 39f) and thread them through the four holes on the underside of the Base and into the Motor Assembly (Fig. 39g).

**Fig. 40:**
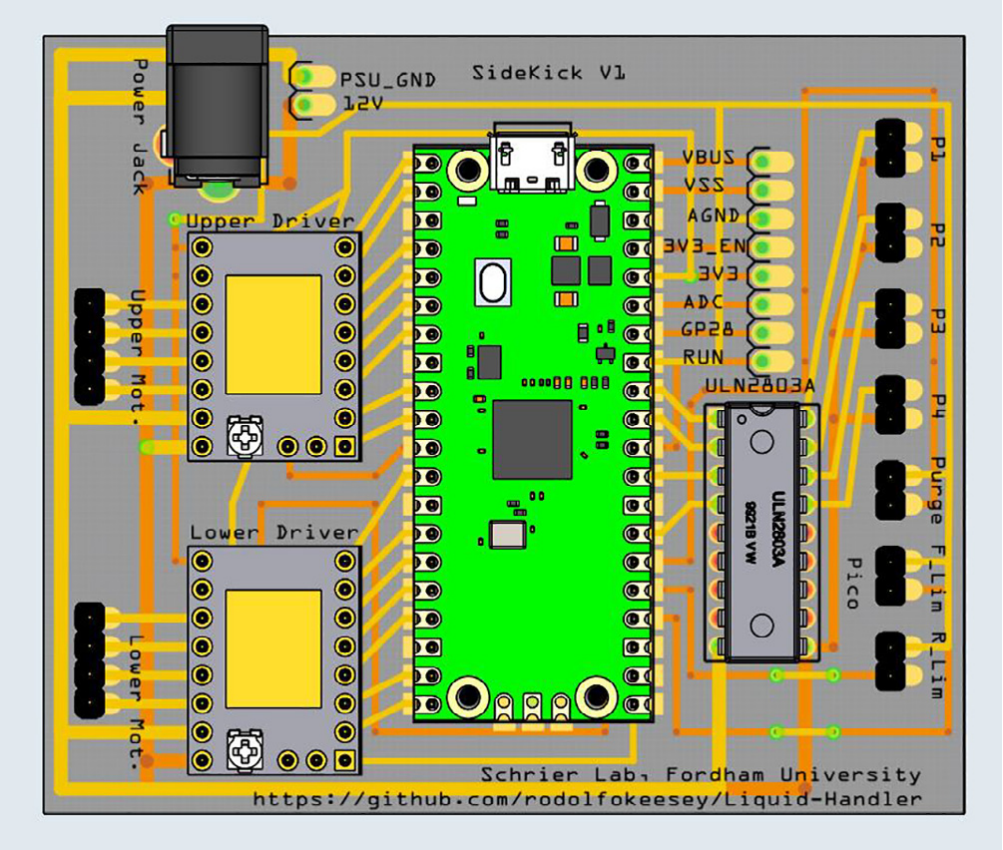
The Sidekick PCB and component layout. The corresponding electrical schematic is shown in Fig. 5.

5G: PCB Wiring/Assembly. (Fig. 40)

Step 1: Soldering in the Electrical Components.

**Fig. 41:**
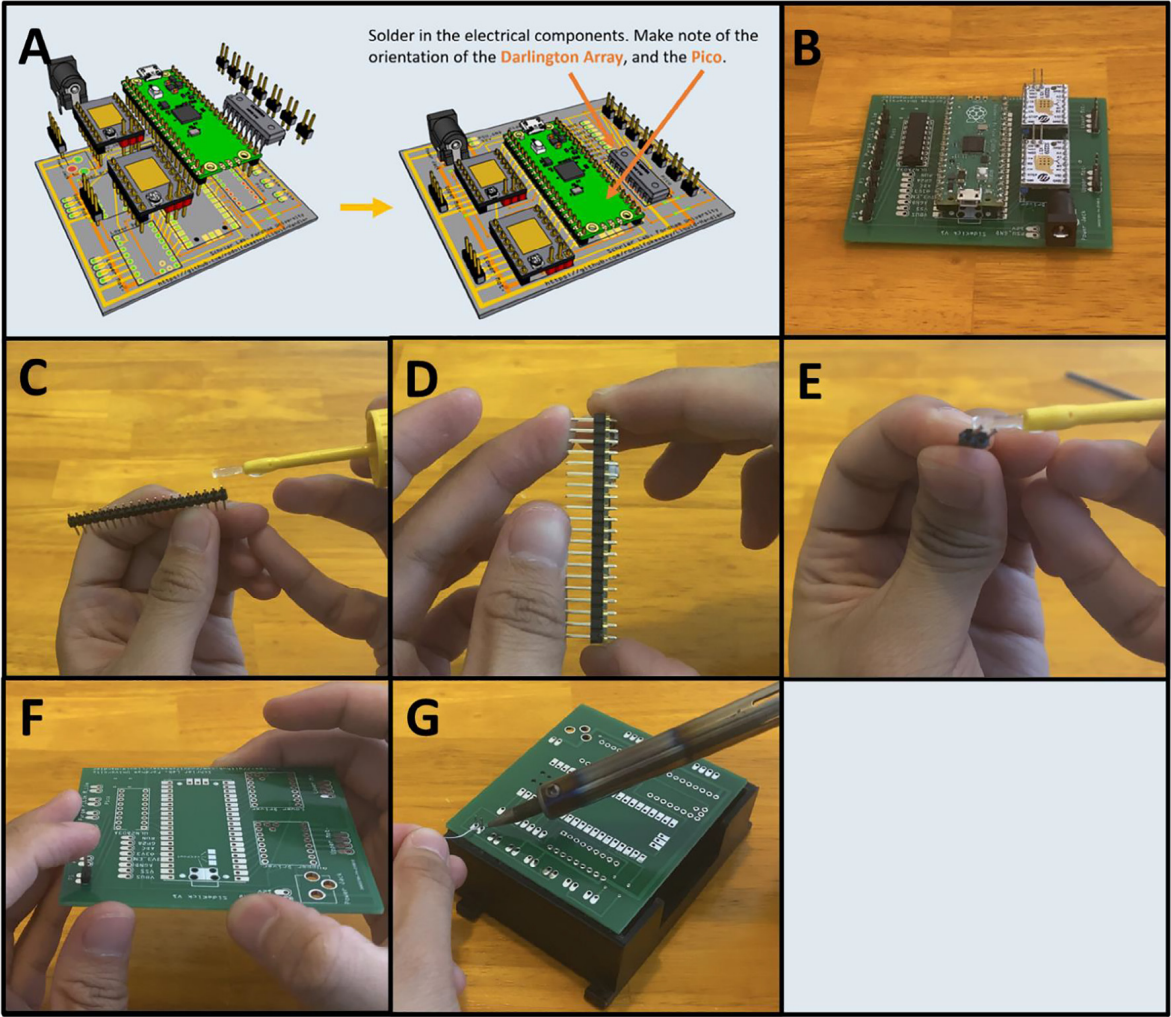
a-g: The procedure for preparing the Sidekick PCB.

Fig. 41a gives an overview of the PCB assembly. Gather the necessary electrical components: the two stepper motor drivers, the power barrel, the Darlington array, the Raspberry Pi Pico and its associated header pins, and 24 header pins (Fig. 41b). If the Pico did not come pre-soldered with its header pins, then solder them on. This is best done by placing a dab of cyanoacrylate glue (Super Glue®) on each end of the header pins where they would touch the Pico board (Fig. 41c). Then press the header pins against the Pico so that the glue forms a light bond to the board (Fig. 41d). This will keep the header pins in place so that they can be more easily soldered. After the Pico has been soldered with the two rows of header pins, prepare the PCB board with header pins. Break off seven sets of 2-long header pins. Dab glue on the end of the 2-long header pins (Fig. 41e) and press it against the PCB into the two pin slots until the glue dries, holding the pins in place (Fig. 41f). It helps to use the PCB tray as a platform to solder the pins on (Fig. 41g). Repeat this process for the rest of the header pin holes. In total, there are seven 2-long header pins, and two 4-long header pins. Then, using the same method, solder the Pico, the stepper motor drivers, power barrel, and the Darlington array onto the board.

Step 2: Trim the electrical component pins.

**Fig. 42:**
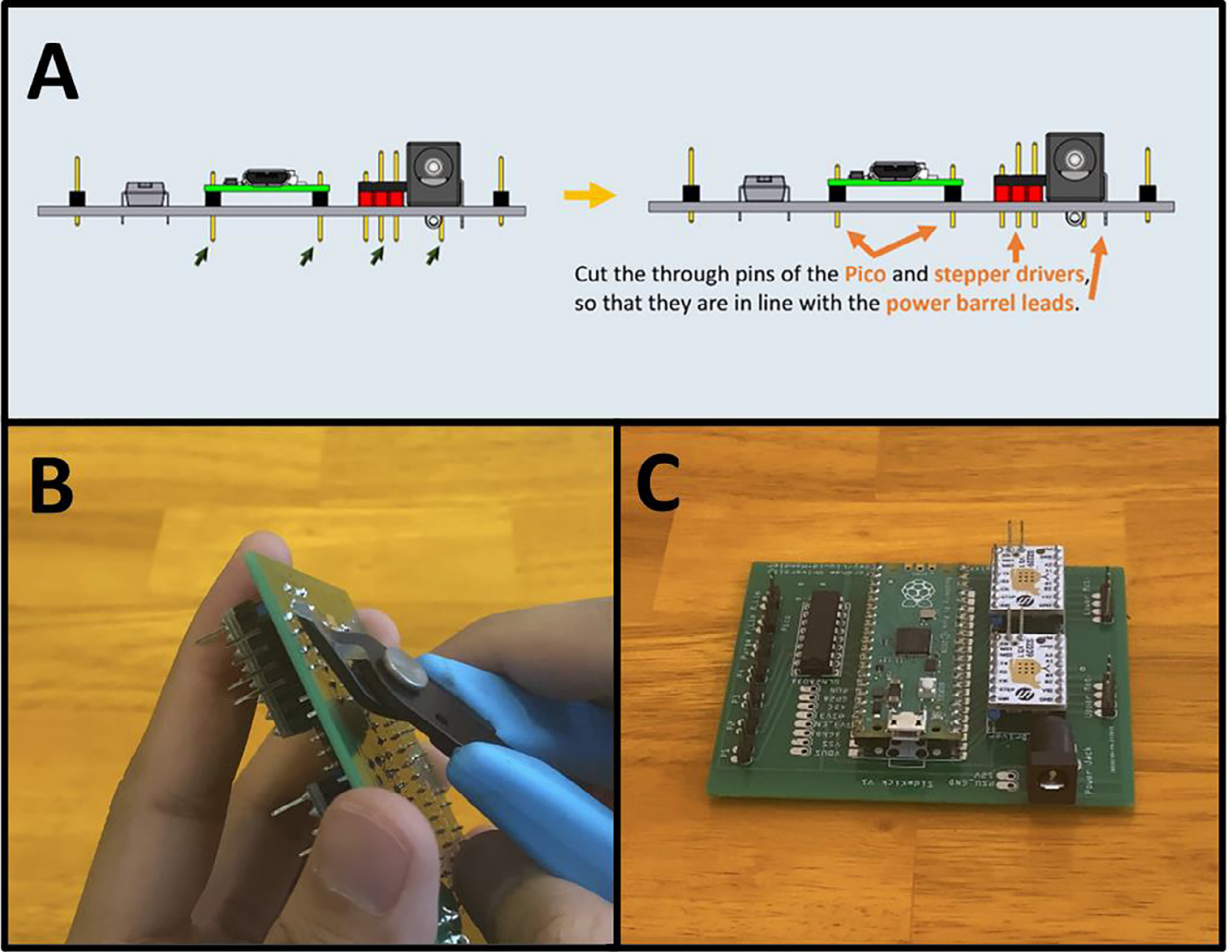
a-c: The procedure for trimming the header pins.

Fig. 42a gives an overview for the pin trimming process. Cut the excess pin length from the Raspberry Pi Pico, and the two stepper drivers (Fig. 42b). They should be the length of the power barrel leads. This allows for the PCB to slide into the PCB Tray. The resulting prepared PCB should look like Fig. 42c.

Step 3: Wiring the Hardware to the PCB.

**Fig. 43:**
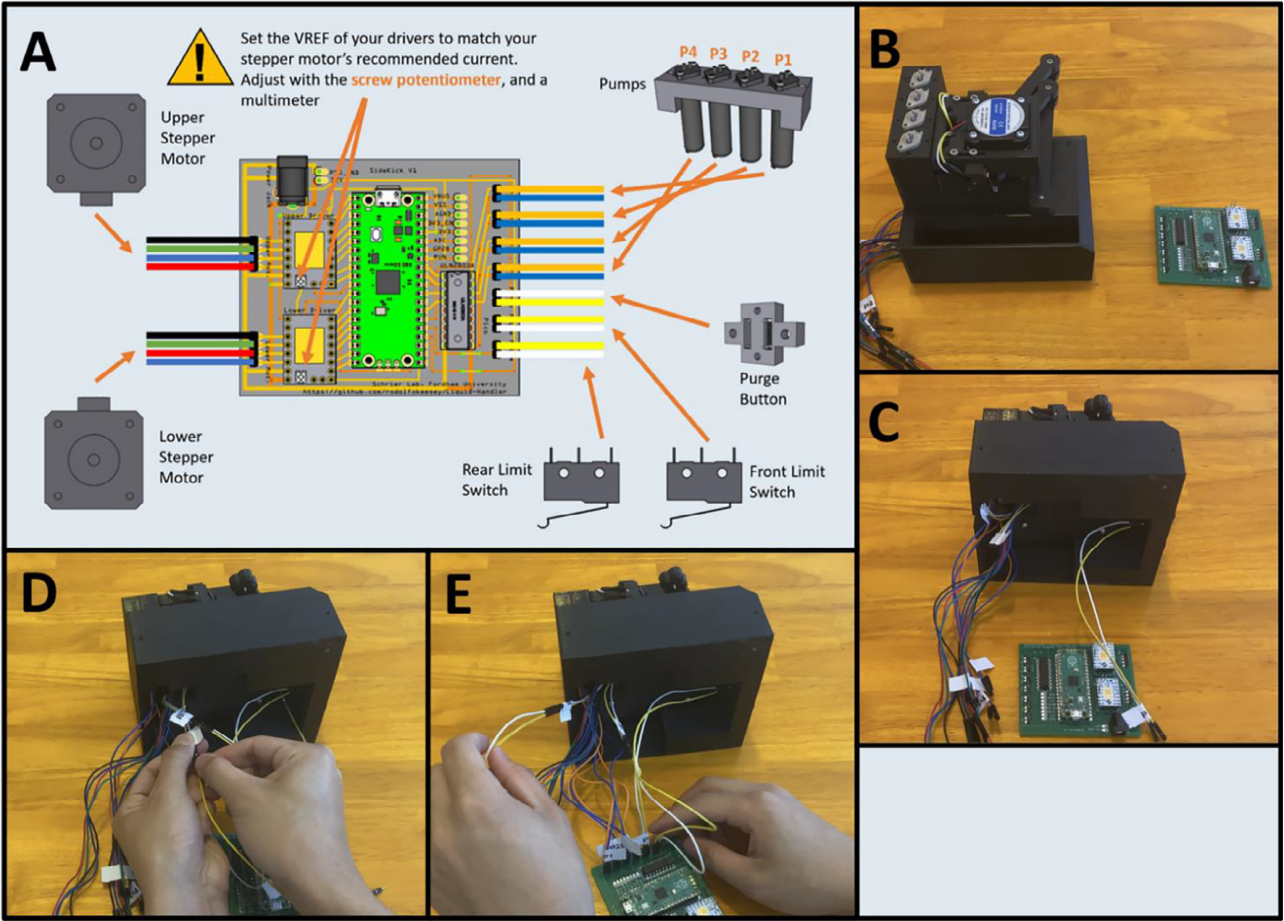
a-e: The procedure for connecting the wiring to the PCB.

Fig. 43a gives an overview of the connection procedure, and the wiring diagram for each of the pins. Gather the Sidekick and the completed PCB (Fig. 43b). Route all wires from underneath the base of the Sidekick (Fig. 43c). If the Dupont connectors for the limit switches are too short, use male-to-female connectors of the same color to extend them out further (Fig. 43d). Plug the female ends of the Dupont connectors into the PCB (Fig. 43e). Be sure to match the wiring colors to the pins correctly as diagrammed in Fig. 43a.

Step 4: Setting Stepper Driver V-Ref.

**Fig. 44:**
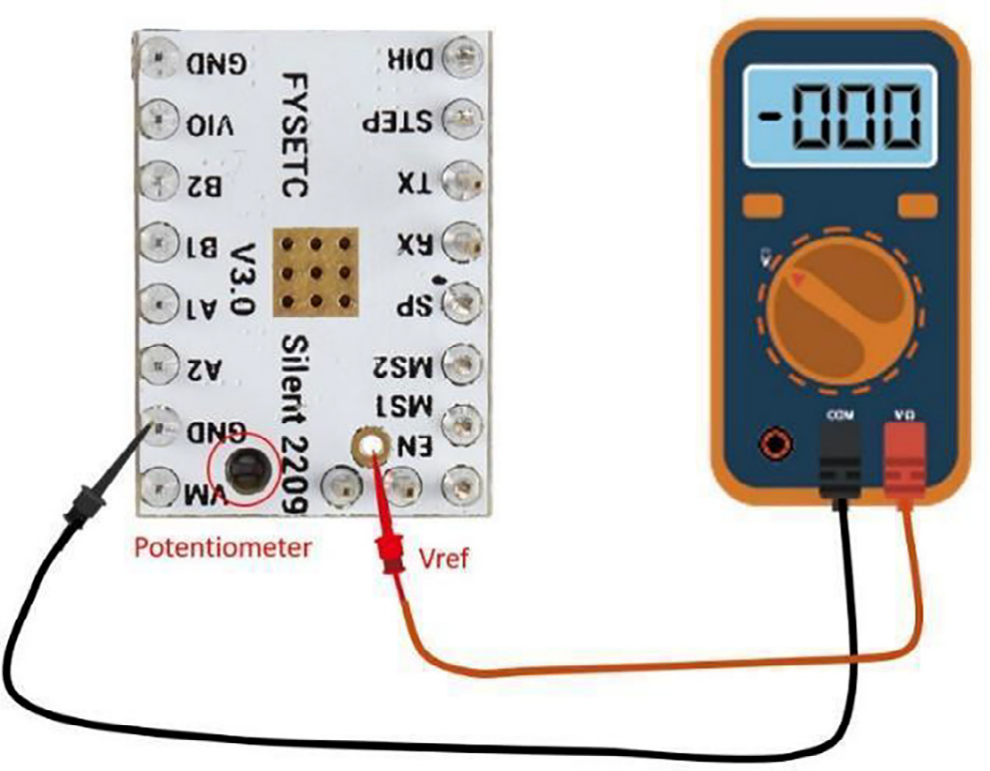
A schematic for how to adjust the VRef of the stepper drive. This figure is from Ref. [38].

After wiring all the hardware, set the V-Ref on the stepper motor drivers to 0.85 V (Fig. 44). The reference voltage, V-Ref, sets the current output of the stepper driver. Using a multimeter with the power jack of the PCB plugged in, attach the negative probe to the GND pin, and the positive probe to the V-Ref pin noted in Fig. 44. Adjust the voltage using the potentiometer, a multimeter, and a small flat head screwdriver. The location of the potentiometer is circled in Fig. 44. If you are using a different stepper motor from the one in the bill of materials, you will need to use the information sheet to determine the correct current. The formula relating current to V-Ref for the TMC 2209 stepper drivers is:

Current (root mean square) = V-Ref * 0.71.

A more in-depth explanation for setting the V-Ref can be found in the Fysetc Silent 2209 data sheet [38].

Step 4: Slide the Wired PCB into the PCB Tray.

**Fig. 45:**
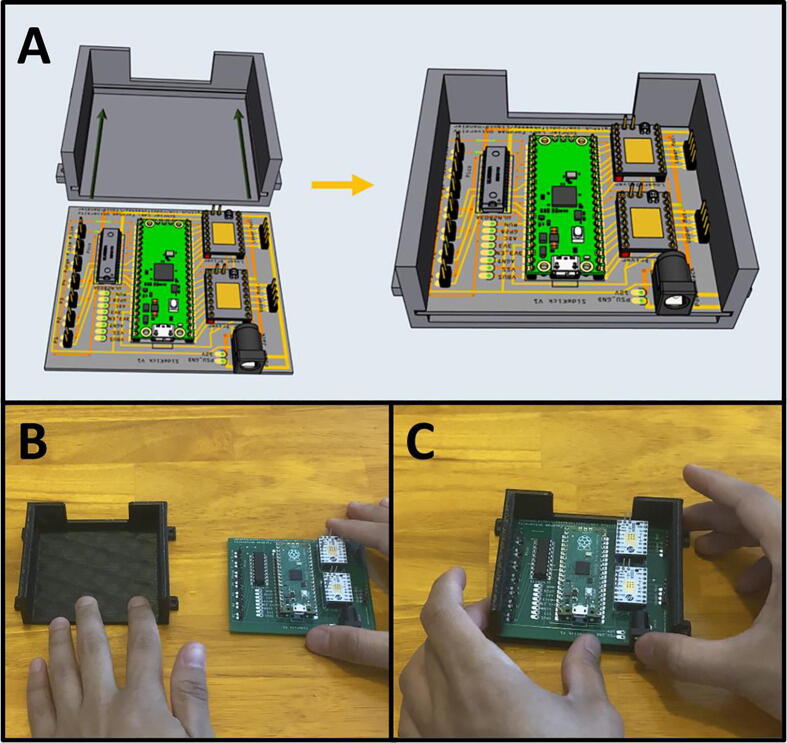
a-c: The procedure for sliding the PCB into the tray.

Fig. 45a shows an overview of the procedure. Slide the PCB into the PCB Tray (Fig. 45b-c). Route the wires into the gap at the rear of the tray.

Step 5: Ensuring Correct Wiring.

**Fig. 46:**
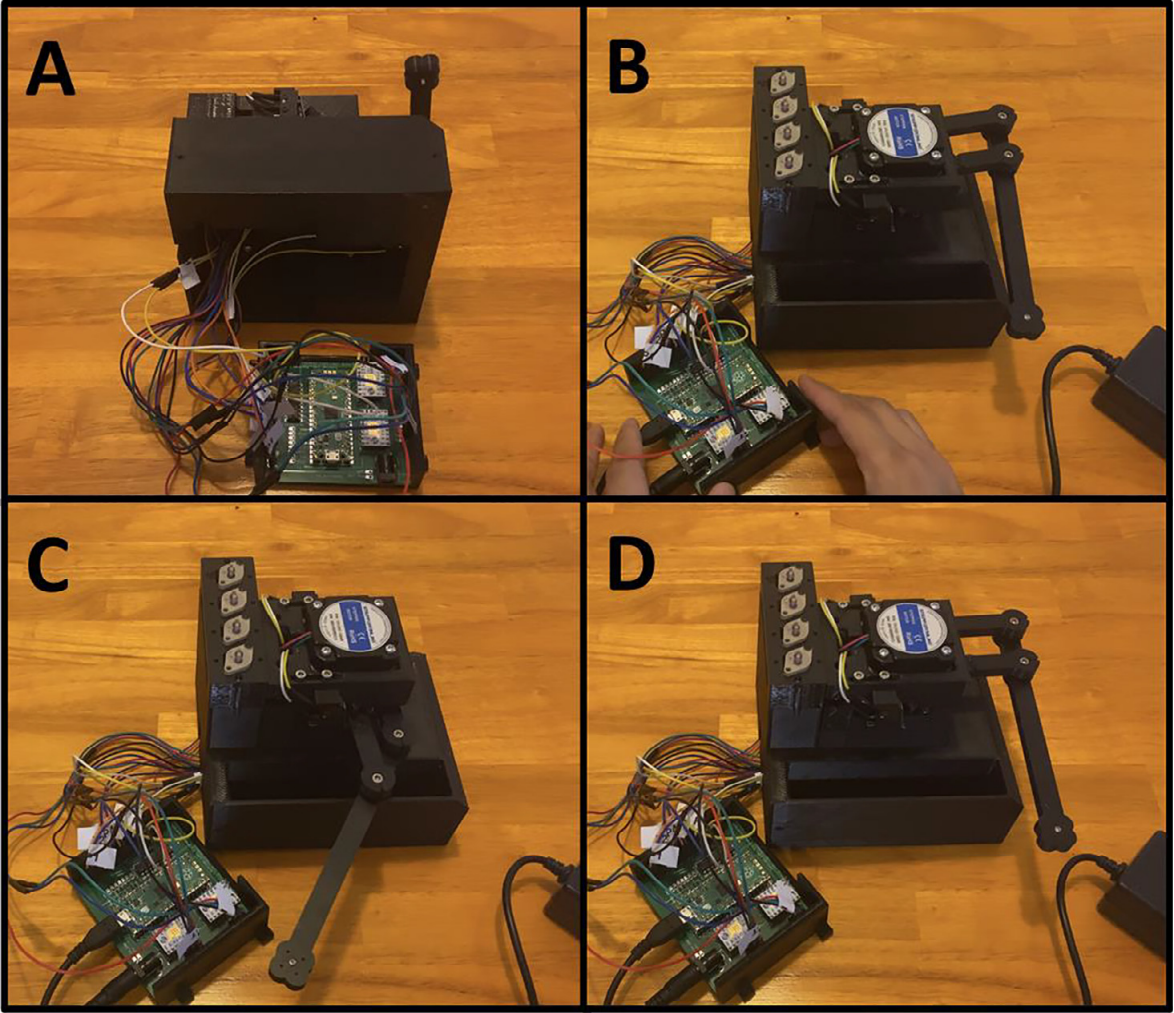
a-d: The homing process for the Sidekick.

Before continuing, make sure that all hardware is wired correctly. Once all the wires are correctly plugged in (Fig. 46a) Plug in the PCB power barrel and connect the Pico’s USB to a computer (Fig. 46b). Once the Sidekick is plugged in, it should immediately start to home against the limit switches. It will first home against the front limit switch (Fig. 46c), and then the rear (Fig. 46d). If this does not occur, first check the wiring for the limit switches and motors, as the Sidekick will not continue if it cannot home properly.

Next, open Thonny and type into the command line, “hardware check” followed by a return. The Sidekick should home against each limit switch, park the nozzle at a 90° angle, and then cycle through energizing each of the pumps. When a pump is energized, it will make an audible clicking sound. The Sidekick will then ask you to press the purge button. Once pressed, it will output “pressed” and when let go, it will output “released”. Once the correct wiring is validated, continue to the next step. If any of the components did not behave as described, inspect their associated wiring.

Step 6: Mounting PCB Assembly to Base.

**Fig. 47:**
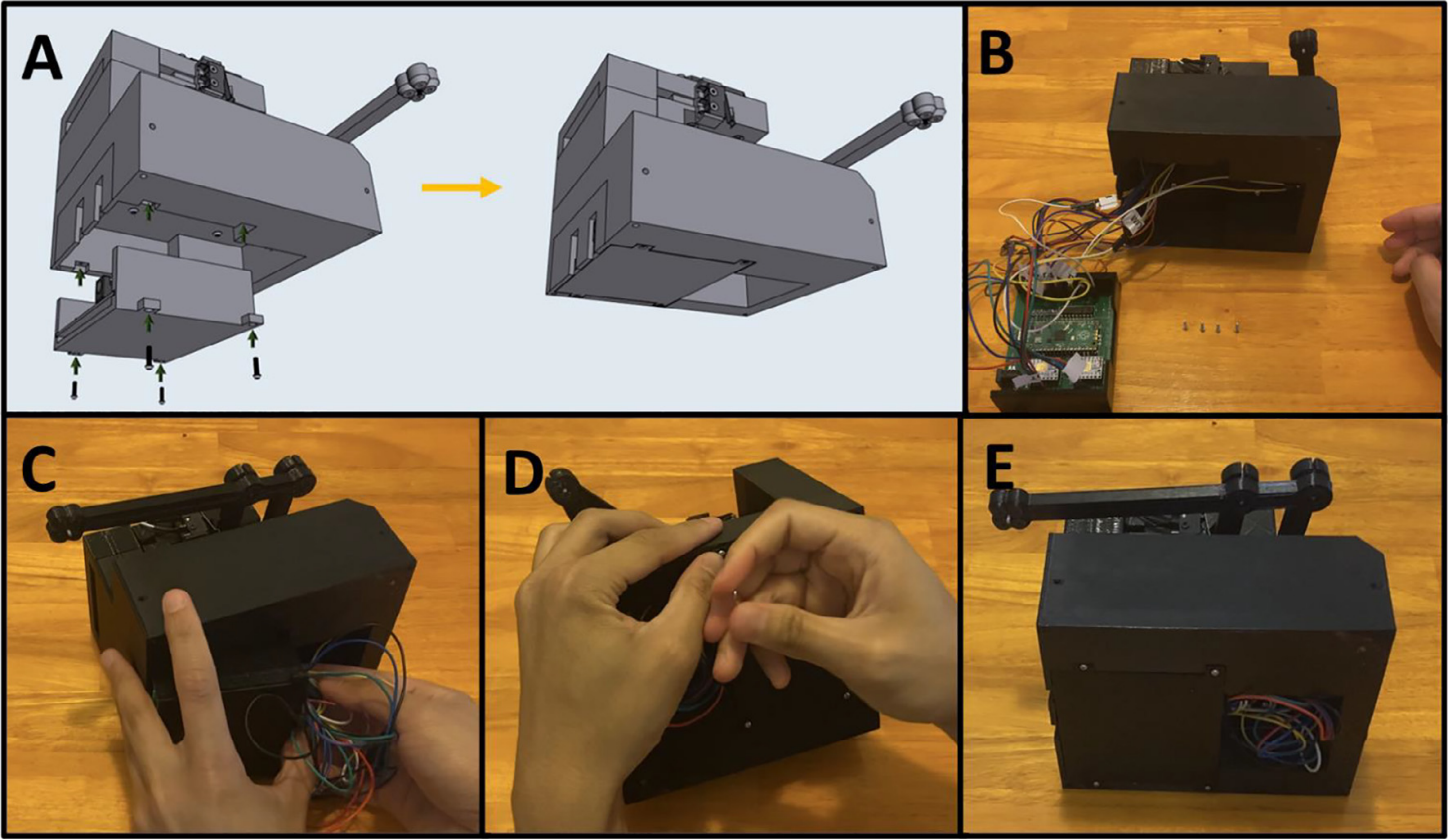
a-e: The procedure for mounting the PCB tray to the Base.

Fig. 47a gives an overview for the mounting process. Gather the Sidekick and four M2 × 8 screws (Fig. 47b). Route all the wires to the gap in the PCB Tray, and the associated gap in the Base (Fig. 47c). Then, fit the tray into the Base slowly, making sure to keep all the wires routed into the cable management gap. Then thread four M2 × 8 screws to secure the tray in place (Fig. 47d) The cables should all be tucked into the gap in the Base (Fig. 47e).

**Fig. 48:**
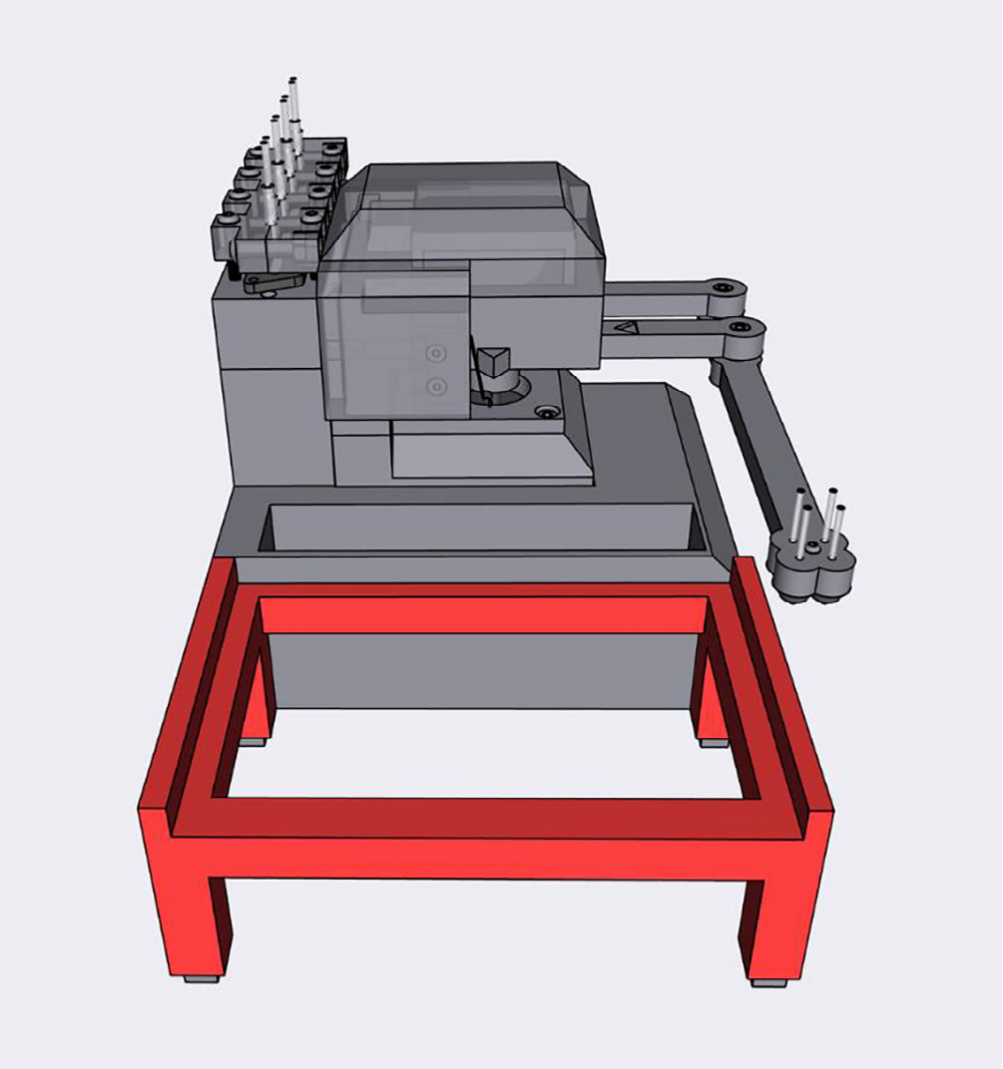
The Sidekick Plate Holder.

5H: Plate Holder and Feet. (Fig. 48)

Step 1: Attach Feet.

**Fig. 49:**
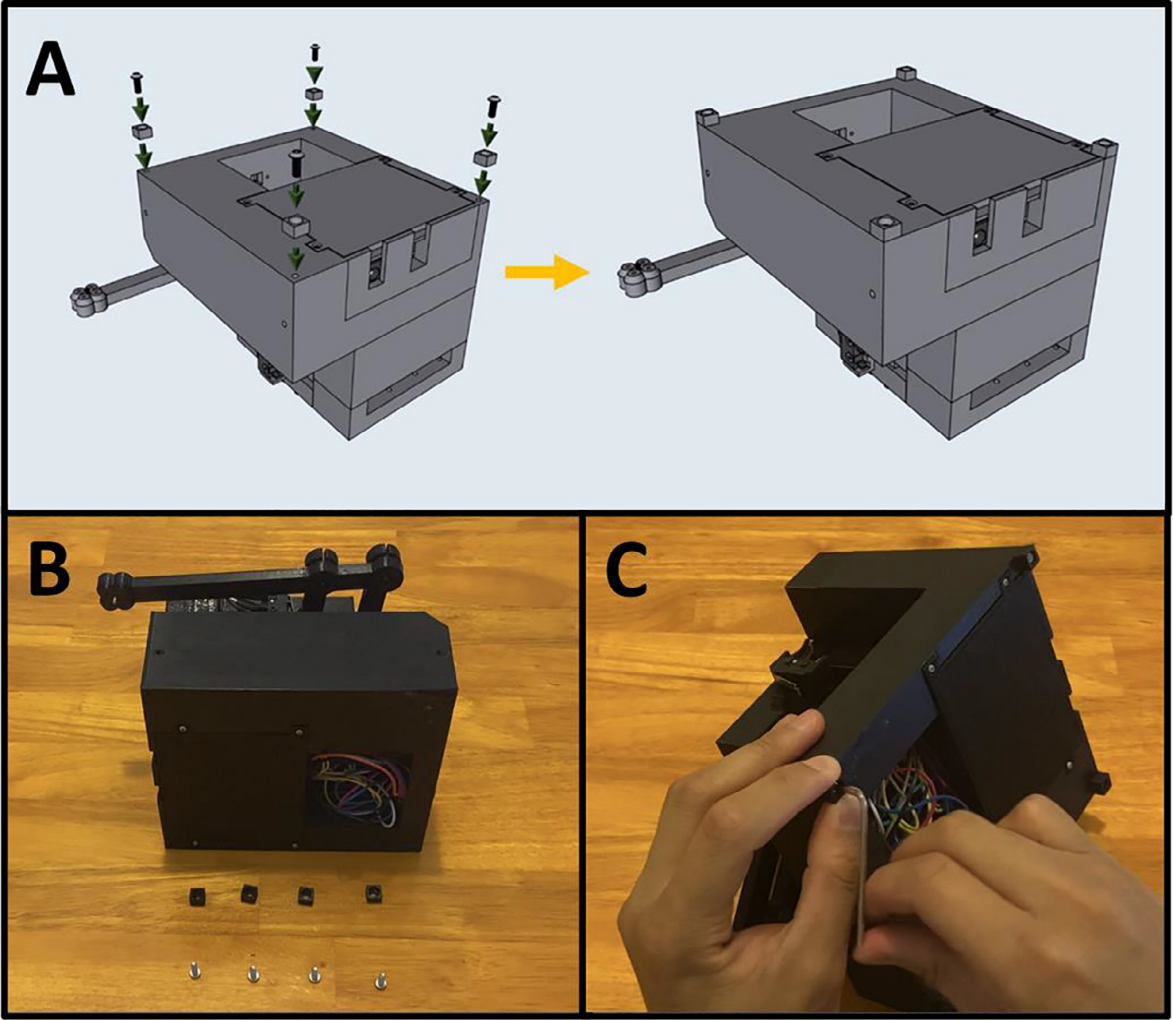
a-c: The procedure for attaching the feet to the Sidekick.

Fig. 49a gives an overview for attaching the feet. Gather the Sidekick, four 3D-printed feet, and four M3 × 8 screws (Fig. 49b). Attach the feet to the corners of the Sidekick’s base (Fig. 49c).

Step 2: Attach Feet to the Plate Holder.

**Fig. 50:**
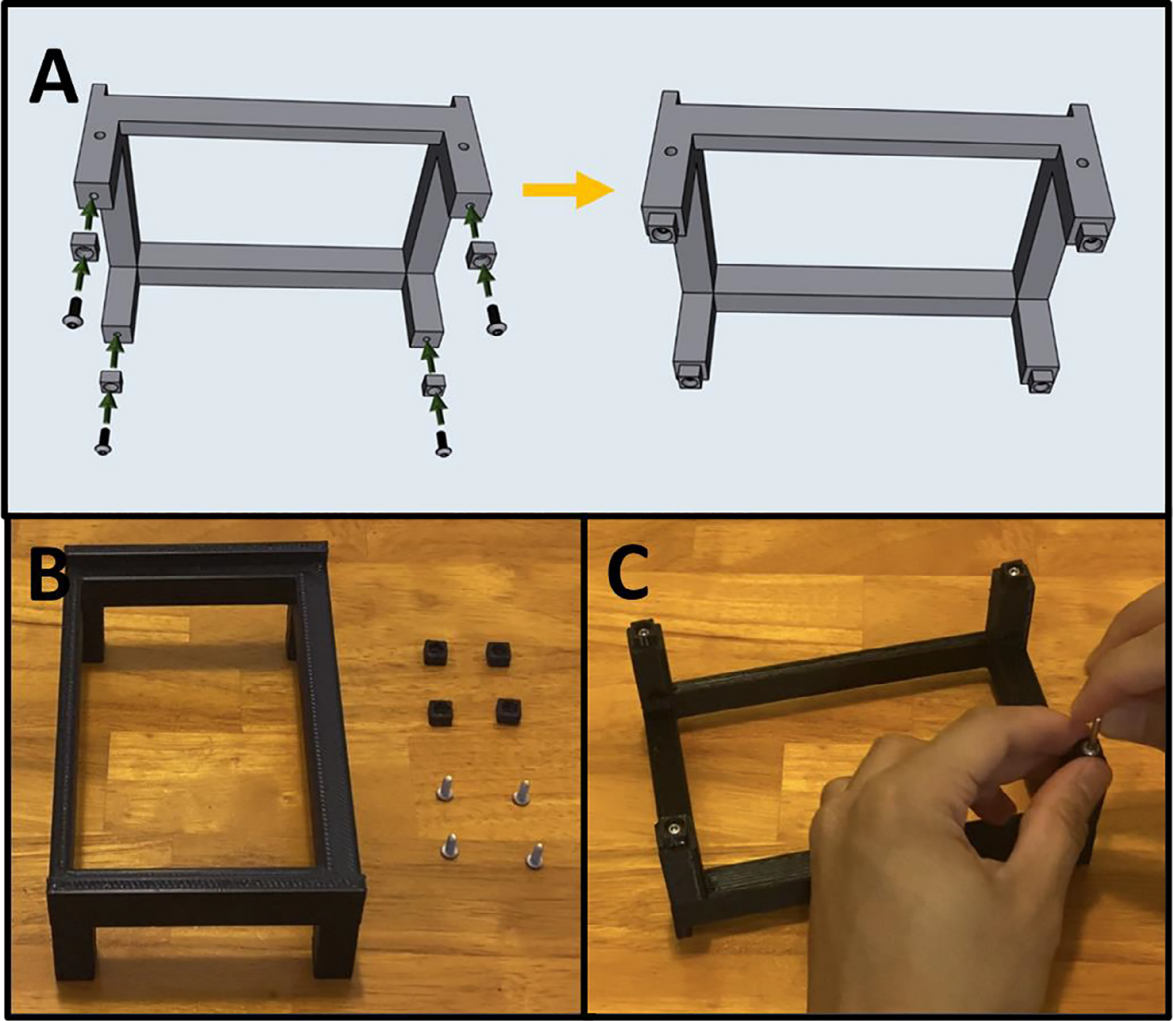
a-c: The procedure for attaching the feet to the plate holder.

Fig. 50a gives the procedure for attaching the feet. Gather the 96 Well Plate Holder, the remaining four feet, and four M3 × 8 screws (Fig. 50b). Attach the four feet to the 96 Well Plate Holder with the four screws (Fig. 50c).

Step 3: Attach Plate Holder to Base Assembly.

**Fig. 51:**
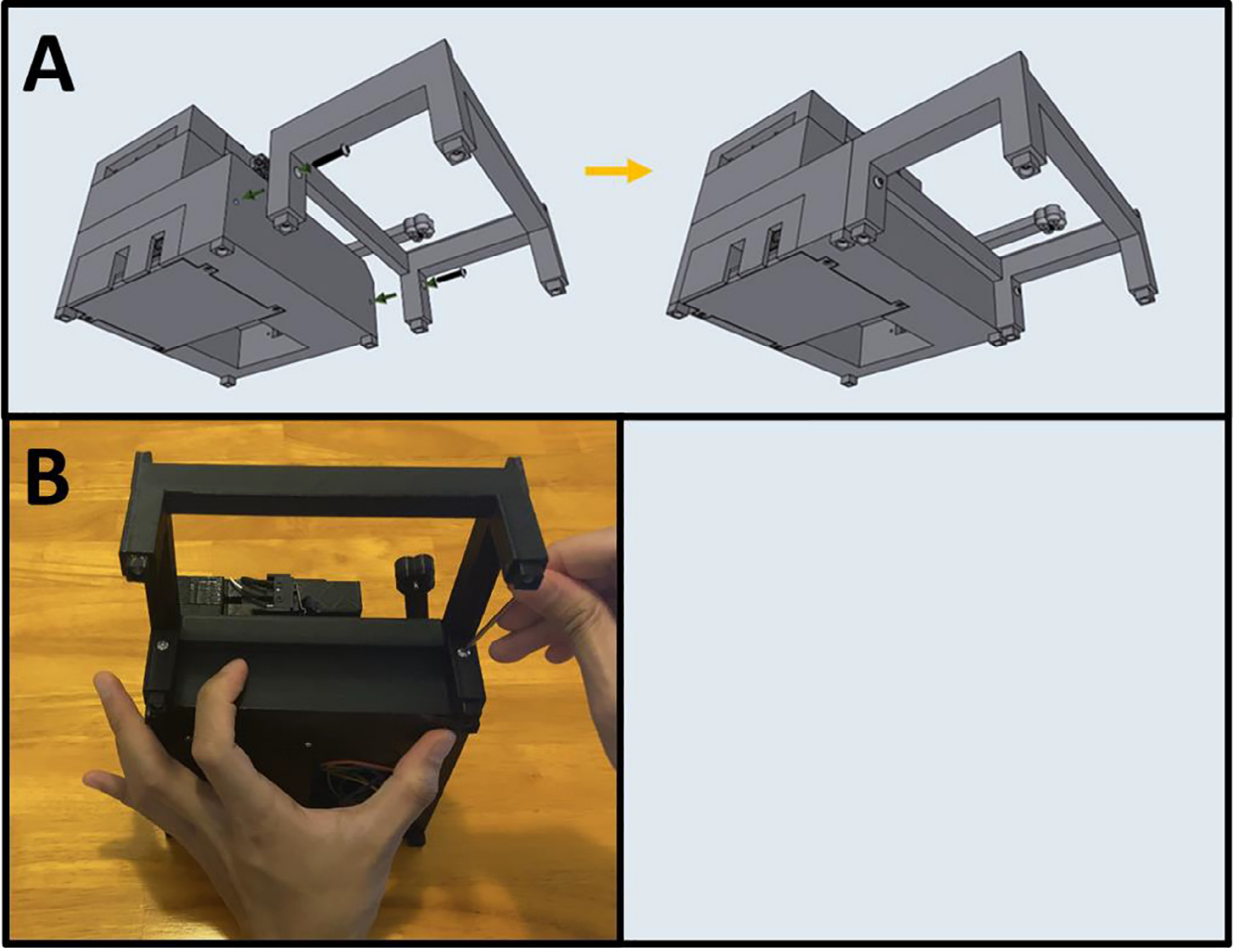
a-b: The procedure for attaching the plate holder.

Fig. 51a gives an overview for attaching the plate holder. Fasten the 96 Well Plate Holder to the Base assembly using two M3 × 16 screws (Fig. 51b).

**Fig. 52:**
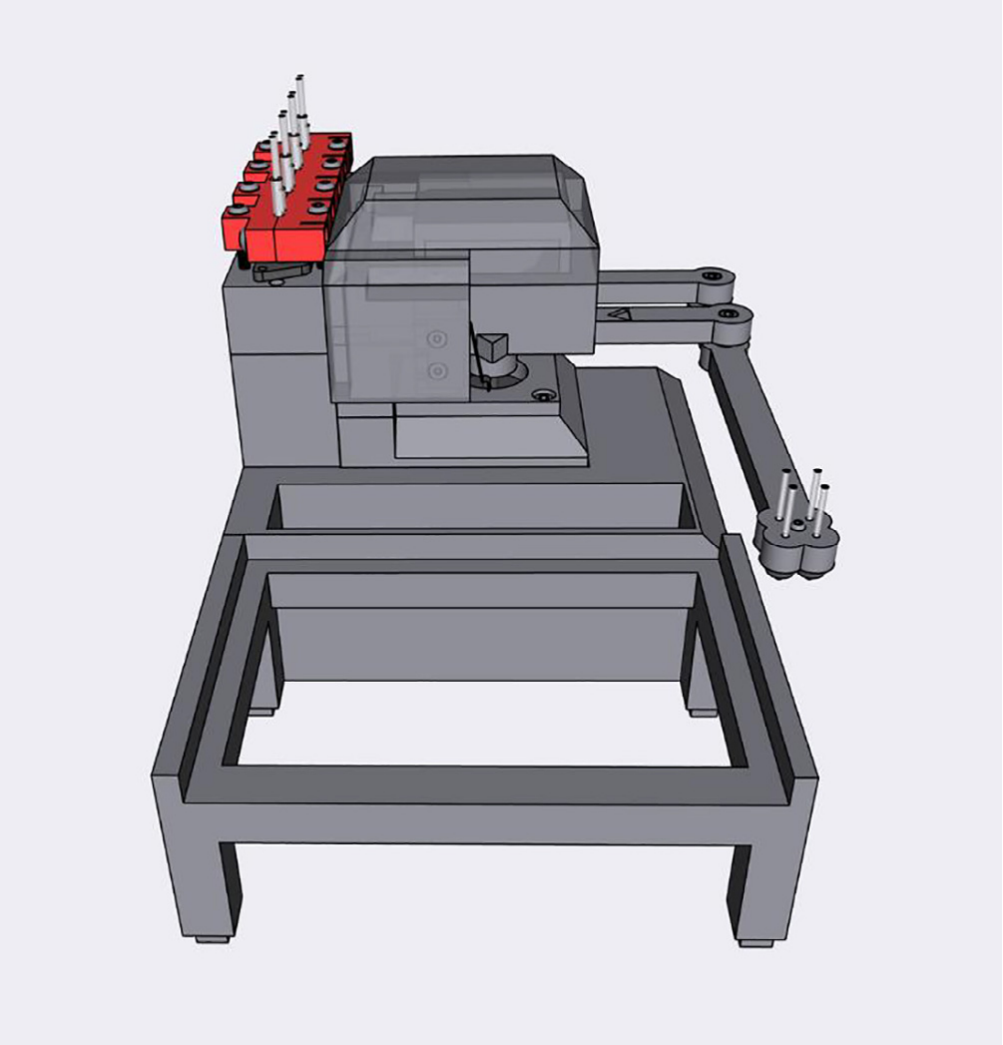
The Sidekick Tubing.

5I: Assembling the Adapter Clamp and Tubing. (Fig. 52)

Step 1: Assemble the Tubing.

**Fig. 53:**
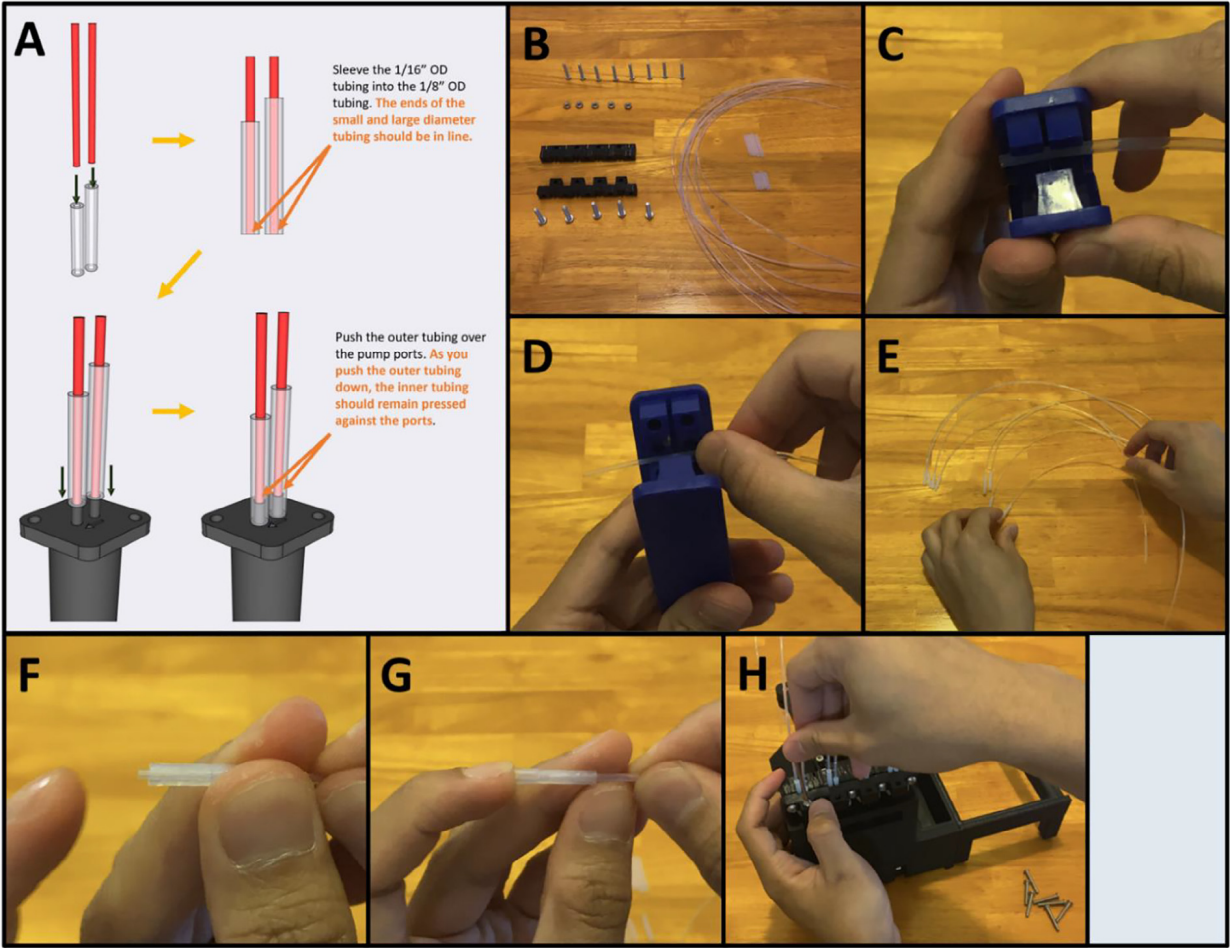
a-h: The procedure for preparing the tubing and adapter.

Fig. 53a gives an overview of the tubing preparation. Gather the 3D-printed Front Adapter Clamp, the Rear Adapter Clamp, five M4 nuts, five M4 × 20 screws, five M3 × 20 screws, 1/8″ OD tubing, and 1/16″ OD tubing (Fig. 53b). With tubing cutters, cut four sections of 1/8″ tubing to 1.7 cm and four sections to 2.0 cm (Fig. 53c). The 1.7 cm tubing is for the outlet port, and the 2.0 cm is for the inlet port. Cut eight sections of the 1/16″ tubing to 30 cm (Fig. 53d). Insert the 1/16″ tubing into the 1/8″ tubing (Fig. 53e). Align the inserted tubing so that the ends are aligned (Fig. 53f-g). Then, press the outer diameter tubing over the ports of the pumps (Fig. 53h). As you push the outer tubing down, the inner tubing should remain pressed against the ports, as shown in Fig. 53a. The arrow on the port face of the pump indicates the direction of liquid movement. Repeat eight times, for each of the pump inlet and outlet ports. **Please note, the pump port distends the outer tubing. Repeatedly removing and** **reattaching the tubing will affect the seal.** It may be necessary to cut fresh tubing if it becomes stretched to the extent that it no longer makes a tight seal.

Step 2: Starting the Adapter Clamp.

**Fig. 54:**
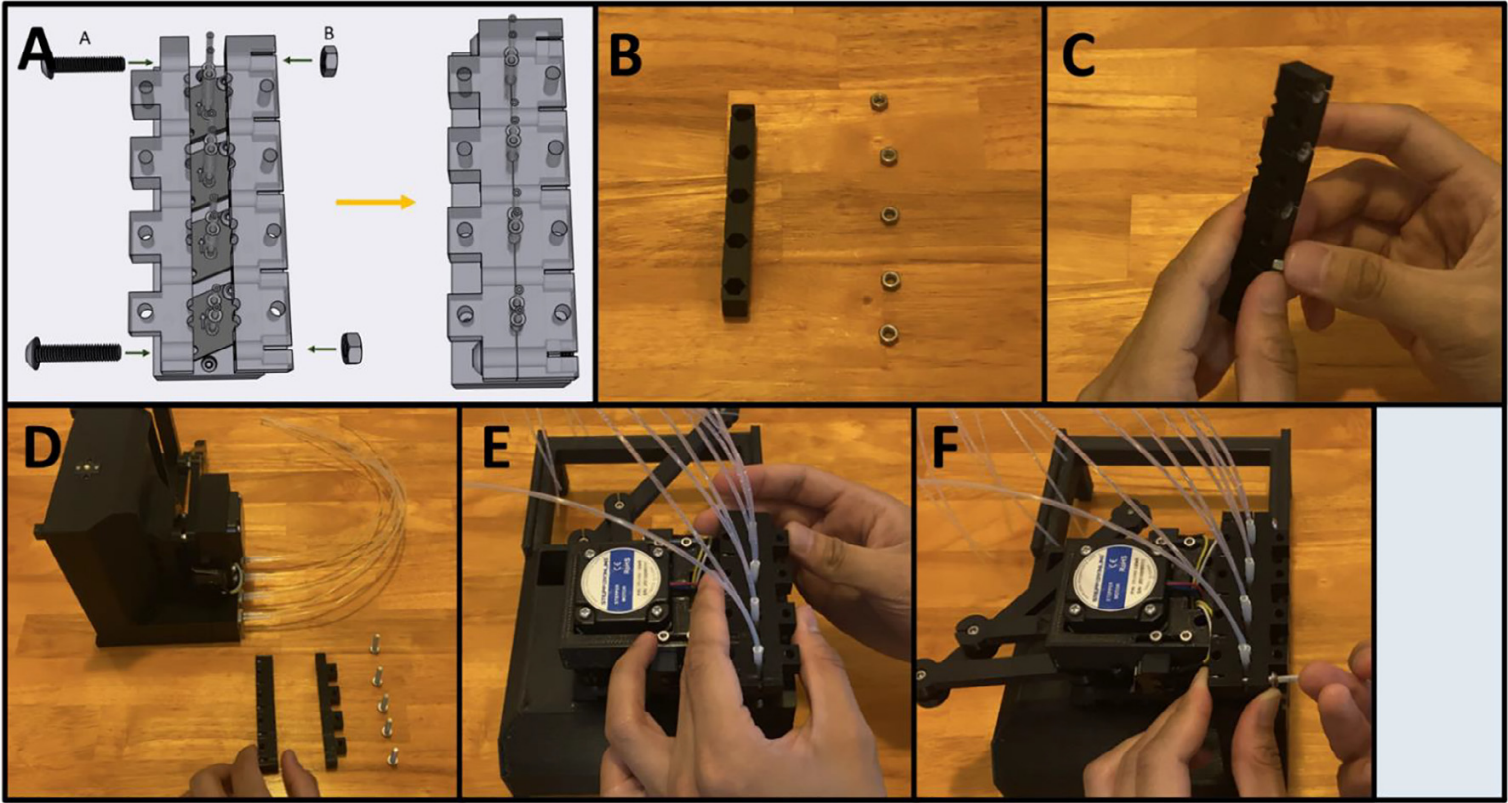
a-f: The procedure for assembling the adapter clamp.

Fig. 54a gives the procedure for assembling the adapter clamp. Gather the Front Adapter Clamp, and the Rear Adapter Clamp, as well as five M4 nuts (Fig. 54b). Press the nuts into the back of the Rear Adapter Clamp (Fig. 54c). Gather the Sidekick and five M4 × 20 screws (Fig. 54d). Sandwich the pump tubing in between the Front and Rear Clamp (Fig. 54e). The Rear Clamp (The one with the captive nuts) should be facing inwards, towards the Sidekick. With two M4 × 20 screws, loosely tighten the outer M4 nuts to hold the clamp in place (Fig. 54f).

Step 3: Position and Finish Tightening the Adapter Clamp.

**Fig. 55:**
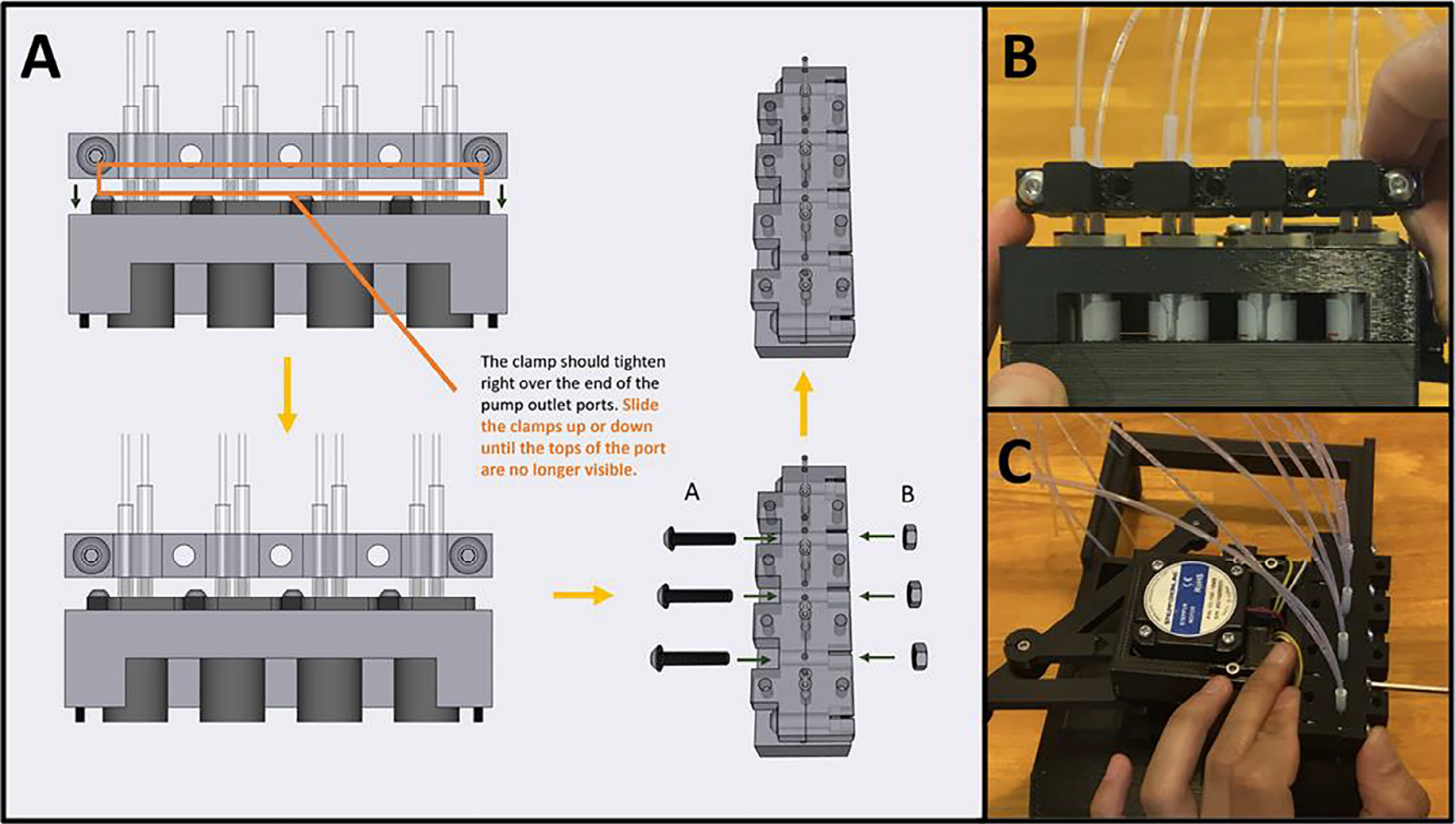
a-c: The procedure for finalizing the adapter clamp position.

Fig. 55a gives an overview of setting the final position of the adapter clamp. The adapter clamp squeezes the outer tubing onto the inner tubing, and then presses the inner tubing into the pump outlet to create an airtight seal. With the clamp loosely tightened, position it just above the port of the pumps. (Fig. 55b). Then screw in the remaining three M4 × 20 screws from the outside in (Fig. 55c). Be sure to tighten all these M4 screws evenly until the clamps are flush together, as they are responsible for squeezing the outer tubing onto the inner tubing. **It will take a significant amount of pressure to mate the clamps together, do not be afraid to thread the M4 screws aggressively**. If a pump is leaking, further tighten the flanking screws. If they cannot be tightened anymore, lightly tighten the screws mentioned in the following step.

Step 4: Threading in the Vertical Compression Screws.

**Fig. 56:**
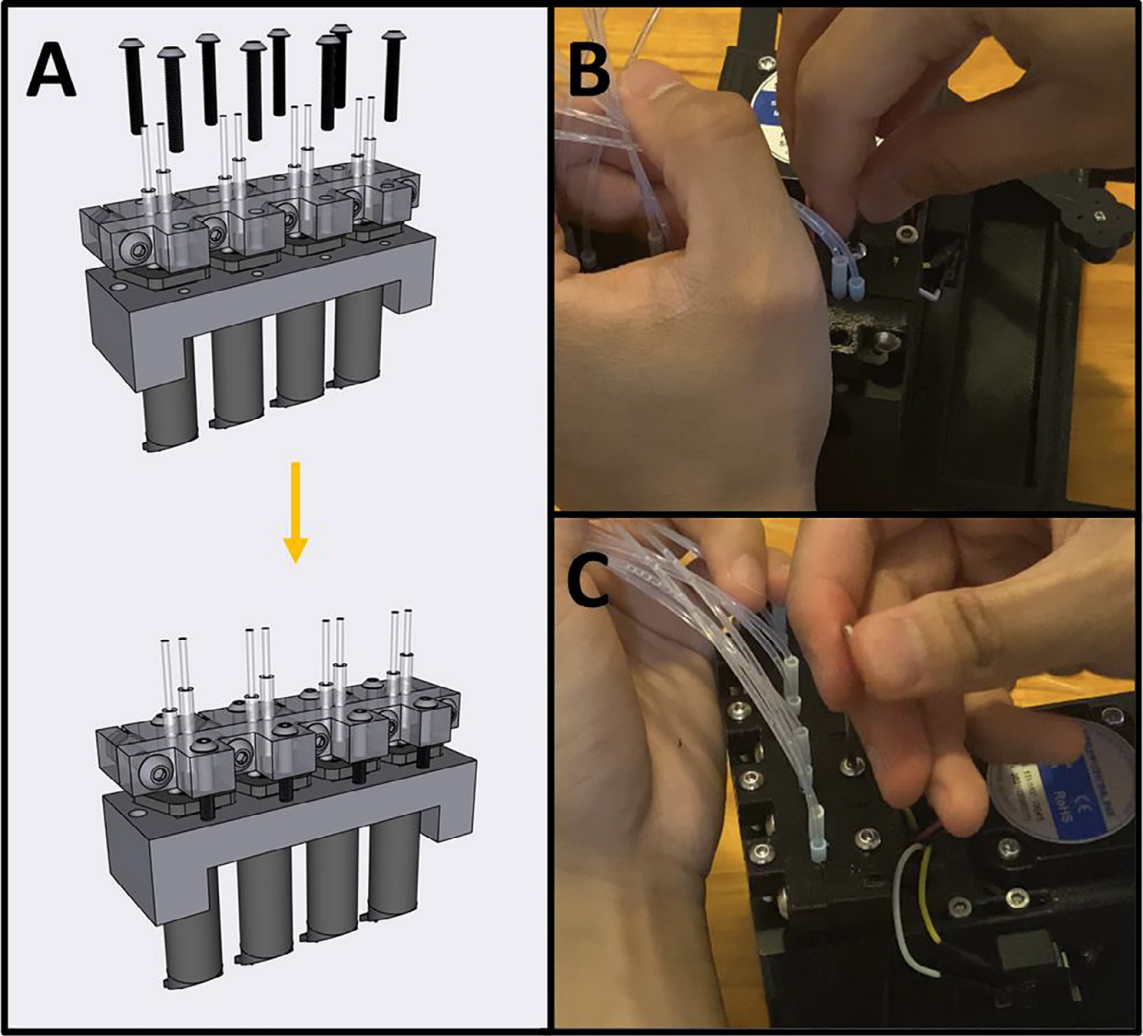
a-c: The procedure for threading in the vertical compression screws.

Fig. 56a gives an overview for threading in the vertical compression screws. Gather eight M3 × 20 screws and tighten them evenly from the outside in (Fig. 56b-c). **Do not overtighten these threads**, you only want to generate enough pressure to press the inner tubing onto the pump ports and but not strip the plastic. If you are still experiencing leaks after tightening both the vertical and horizontal compression screws, you can reprint the clamps, or glue the tubing as a final resort. We printed four different sets of clamps with four different printers: a Creality CR10s Pro V2, a Creality Ender 3, an Ultimaker s5, and a Prusa MK3s. The clamps from the two Creality machines were printed in PETG, and the clamps from the Ultimaker and Prusa machines were printed in PLA. The Creality and Ultimaker clamps were able to achieve leak free operation, but we resorted to glue for the set printed in PLA by the Prusa. **We observed that the rigidity of PLA makes it more difficult to tighten the clamps around the tubing, so we recommend using PETG filament to print the adapter clamps.**

Step 5: Attaching the tubing to the nozzle.

**Fig. 57:**
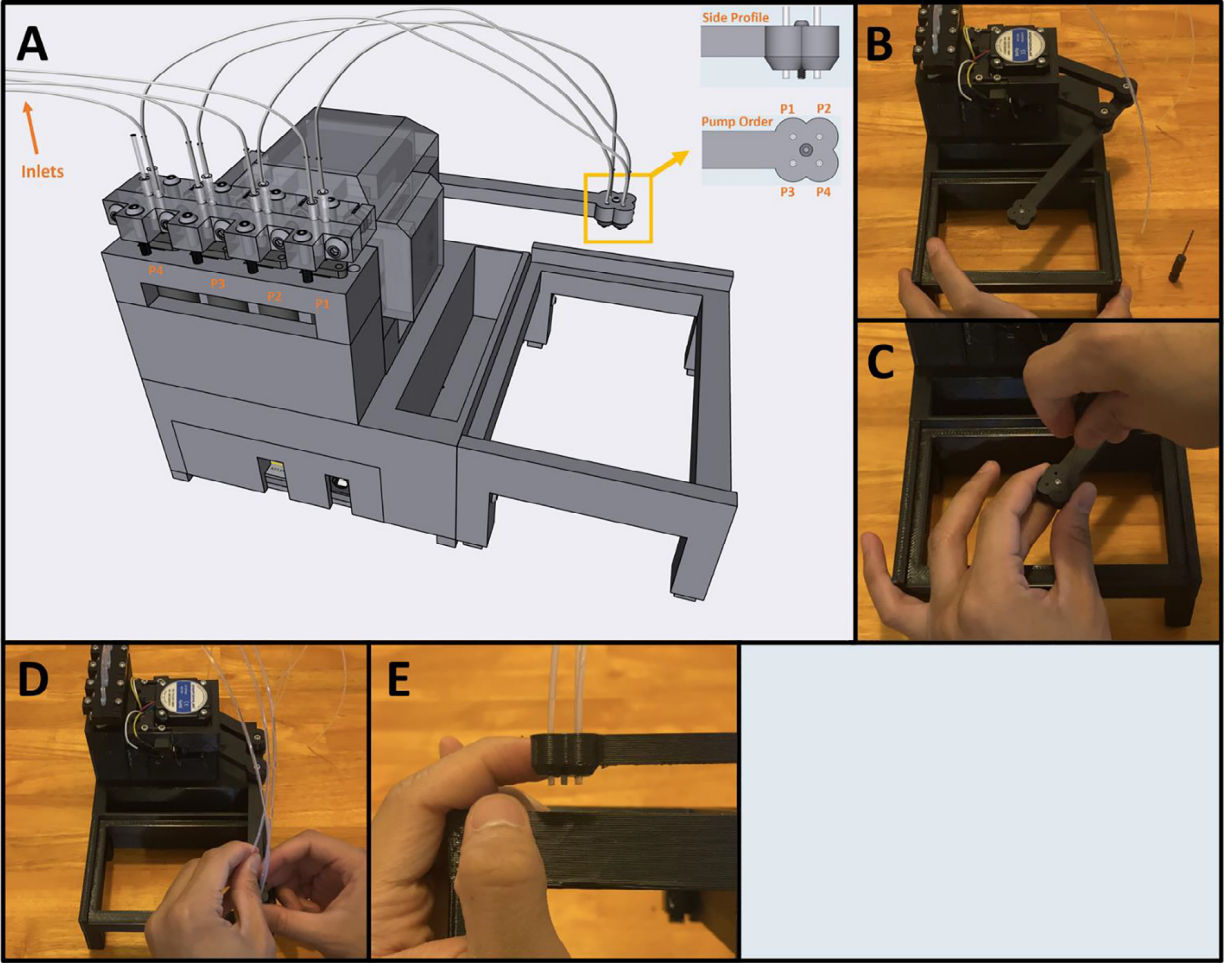
a-e: The procedure for attaching the outlet tubing to the nozzle.

Fig. 57a gives an overview for sleeving the outlet tubing into the nozzle. Gather the Sidekick and a 1/8″ drill bit (Fig. 57b). Match the pump tubing with the pump order given by the diagram in Fig. 57a. There are notches in both the pump holder and the nozzle that indicate the pump order. Press fit the tubing outlet into the nozzle, if the tubing does not fit in the holders in the nozzles, drill them out with the 1/8″ drill bit (Fig. 57c). Once the holders are drilled out, sleeve the outlet tubing in (Fig. 57d). Repeat four times for each pump, then push the tubing through the nozzle until it matches the center screw (Fig. 57e).

Step 6: Creating the Reagent Reservoir.

**Fig. 58:**
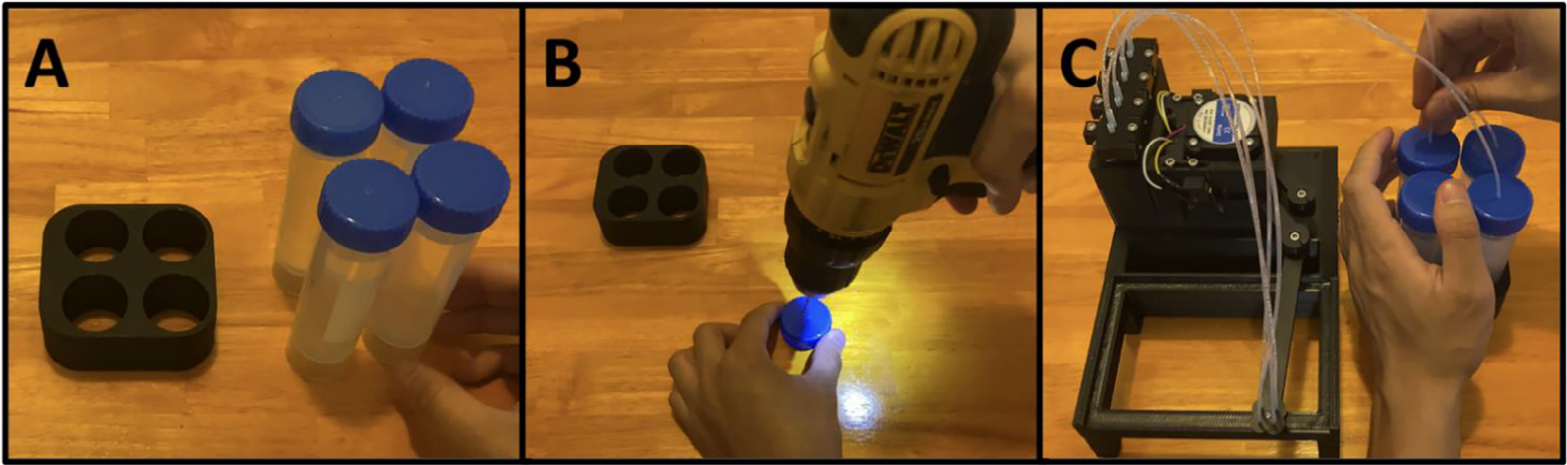
a-c: The procedure for making the reagent reservoir.

Gather the 50 ml Tube Holder and four 50 mL centrifuge tubes (Fig. 58a). With the same 1/8″ drill bit, drill out the caps of all four centrifuge tubes (Fig. 58b). Pass the tubing through the drilled holes (Fig. 58c).

Step 7: Attach the 96 Well Plate and the Purge Vial.

**Fig. 59:**
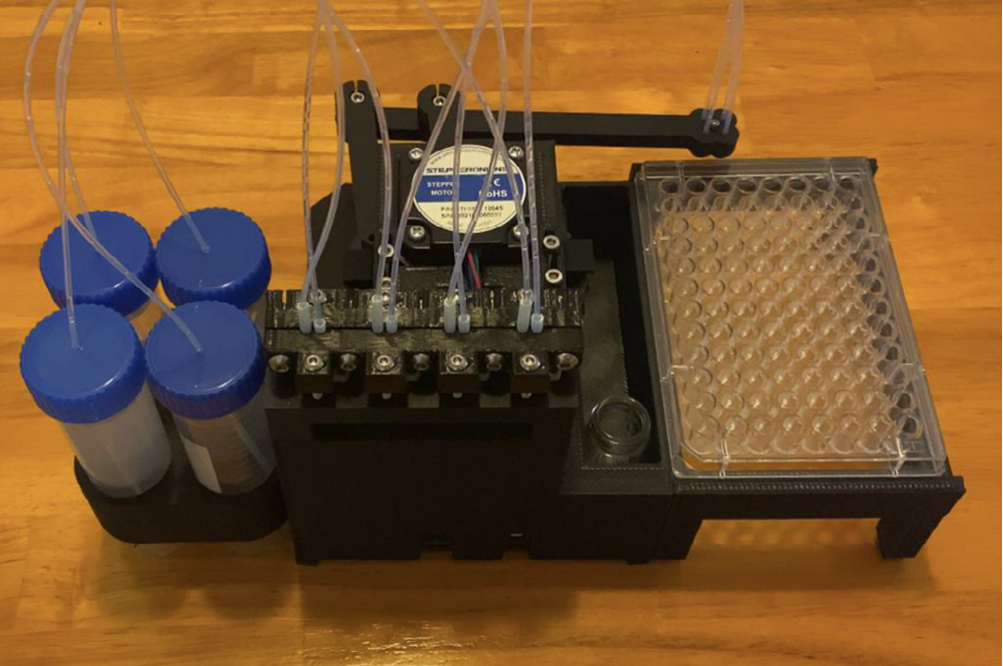
The completed Sidekick.

Slide in the 96-well plate and the purge vial as pictured in Fig. 59. The assembly of the Sidekick is now complete.

**Fig. 60:**
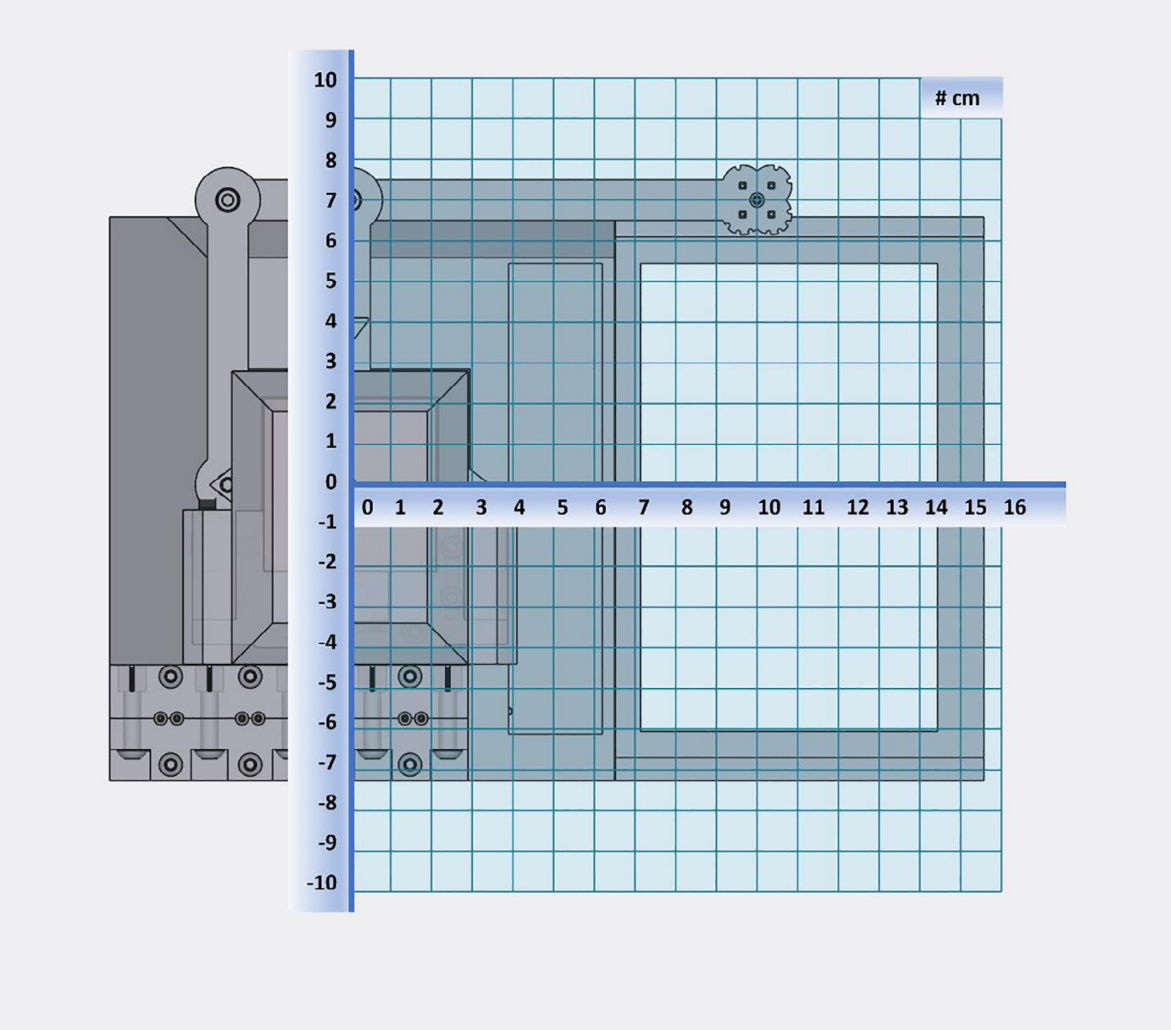
An XY coordinate (cm) overlay to aid in G-Code positioning. Note that the base of the armature is at (0,0).

Operation instructions.

Step 1: Connecting via Thonny’s Serial Console.

After assembling the Sidekick and validating the correct wiring, reconnect the Sidekick to the USB port of the controlling computer, and connect it to the power supply. The Sidekick reads commands from the USB serial port, allowing any application or programming environment that supports serial I/O to communicate with it. For ease of use, we recommend starting with the serial console within Thonny. Once the Sidekick is connected to the host computer, it will start homing against the limit switches. Then type any command into the Thonny serial console. For an example of how to use the Sidekick with another software, an example of connecting through Putty is given in the project’s repository.

Step 2: Calibrating the Plate Map, and setting the Purge Location.

Before doing anything with the Sidekick, first set up the plate map and a purge location. Setting a plate map is important for locational accuracy, as slight variations in assembly may create slight differences from the preloaded map for a standard 96-well plate. To calibrate the Sidekick, load a 96-well SBS microplate into the tray. Then type the command: “remap” (return) and follow the prompts. You can remap as many times as you wish.

The purge location is any arbitrary place that you can use to purge the pump lines and dump excess liquid. The Sidekick has been designed with a tray to accommodate small waste vials, but you can easily set up a purge location elsewhere. To set up a purge location, type the command: “set purge” (return) then follow the prompts. The purge location can always be reset.

Once the purge location has been set, and the plate map recalibrated, your Sidekick is now ready to dispense to a plate. Before dispensing from a pump, clear the air from the lines. This can be done by directing the Sidekick to dispense to the purge location, “p1 purge 1000” or with the “manual purge” command. Repeat this process after leaving liquid in the lines for an extended period, or to clean the lines after changing liquids.

6.2 Command Library:

The Sidekick supports both a limited subset of G-Code (RS-274) commands and a simplified command language that is suited for dispensing into 96-well SBS microplates. In both cases, commands are provided over the USB serial port connection. Before describing the supported commands, we briefly compare and contrast the strengths and weaknesses of these two approaches. G-Code (RS-274) is a widely used instruction set for controlling computer numerical control (CNC) mills and 3d-printers. A set of “G”-prefix commands control motion specification and extrusions/pumping, and a set of “M”-prefix commands provide other miscellaneous features. Because G-Code was developed to support CNC mills and 3D-printers, it is natural to express continuous movements to arbitrary positions and continuous extrusions (expressed as a rate). In contrast however, our goal is to perform motion to a predefined set of discrete locations (the wells in standard SBS microplate, using the standard nomenclature, e.g., “h3”) and to perform discrete dispensing operations using our digital pump only once we have reached that location. Doing this with G-Code requires that the user handle the additional complexity of specifying the X, Y positions of the individual SBS locations and the relevant offsets of each dispensing head. Our simplified control specification hides much of that complexity internal to the parser, allowing direct typing of serial commands or simple programming through a serial port.

### Simplified Command Language

Dispensing and Moving:

A dispense command should be a single line indicating three things:

The desired pump to dispense from: “p1”.

The desired location to dispense to. This can be a well location, or the purge vial location: “a8” or “purge”.

The desired volume in microliters to dispense: “200”.

For example, a command dispensing 200 μL from pump 1 into well H3 is:

“p1 h3 200”.

A movement command should be a single line indicating two things:

The desired pump to move: “p1”.

The desired location to move to: “a8” or “purge”.

For example, a command moving pump 1 into well H3 is:

“p1 h3”.

Things to note:

All commands are case insensitive (e.g., “p1″ and “P1” are identical).

Volume is given in microliters and rounded to the nearest 10 μL increment.

Entries are blank space delimited.

Command List:

**Initialize:** Homes the armature against the limit switches. Use if the Sidekick has bumped against something and skipped steps.

**Hardware check:** Runs through all the hardware to validate that everything has been wired correctly. Use after wiring the Sidekick, or for troubleshooting.

**Free move:** Allows the user to freely move the armature.

**Sleep:** De-energizes the motors, allowing the armature to move freely.

**Wake:** Re-energizes the motors so that the armature can move again. Use after the “sleep” command.

**Return home:** Returns the armature to the home location.

**Manual purge:** Used to purge the liquid lines. Use after swapping reagents, or for cleaning the lines.

**Remap:** Used to calibrate a new plate. Use if swapping in a plate with different well locations.

**Set purge:** Sets the purge location. Use if changing the location of the purge tray/vial.

**Execute Saved Protocol:** Executes a pre-set queue of commands editable in the “saved_protocol.csv” file stored on the Raspberry Pi Pico storage.

#### G-Code support

Users may prefer to use G-Code. Because our kinematics is not supported by standard open-source G-Code firmware software like Marlin [39], and because we want to implement the other features discussed above, we have chosen to implement support for a limited subset of G-Code commands, described below.

**G0 [Ea;b;c;d] [X < pos > ] [Y < pos > ]:** where E defines the extrusion for each of the pumps a;b;c;d (in units of μL, discretized to 10 μL), and X and Y indicate the desired absolute position of the center of the dispense head (in units of millimeters). Figure 60 shows the underlying coordinate system for these operations. As in standard G-Code, these can be provided in any order. If the X or Y positions are omitted, then the current value is used. We assume that motion to the desired coordinates is performed first, followed by the dispensing operation specified by the extrusion command.

**G28:** move to home; equivalent to “return home”.

**G29:** performs calibration; equivalent to “remap”.

**M17:** enables steppers; equivalent to “wake”.

**M18:** disables steppers; equivalent to “sleep”.

**M24:** executes the “saved_protocol.csv” file stored on the Raspberry Pi Pico storage; equivalent to “execute saved protocol”

## Validation and characterization

The accuracy of the armature movement was determined qualitatively by plating liquid to each well and ensuring the Sidekick can accurately dispense from each channel into each well. The volumetric accuracy and precision of the pumps were tested through gravimetric analysis of deionized water. We built two Sidekick devices and measured their performance to get a sense of both the variation of the pumps and construction processes.

**Fig. 61:**
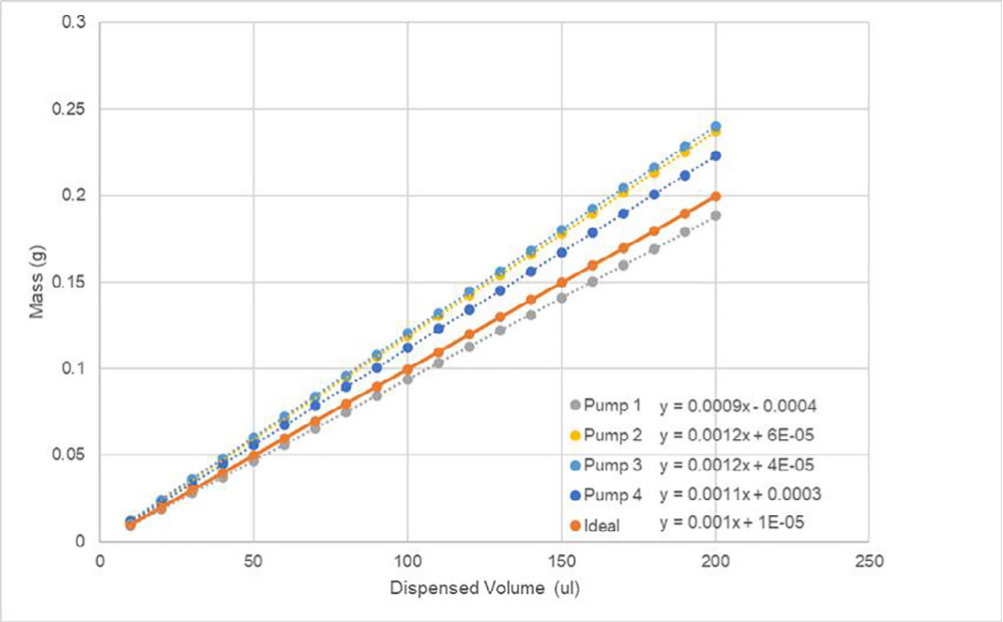
Pumps 1–4 dispensed water mass in a series of 10 μL aliquots.

In a test of the first Sidekick’s multichannel-pump accuracy, a nominal 10 μL of water (the volume of one dispense cycle) was pipetted 20 times by each pump, and the resulting mass was recorded. The gravimetric data for each of the pumps 1–4 are given in Fig. 61. A second gravimetric test was run with nominal 100 μL aliquots repeated 11 times. Given that volumes greater than 10 μL are dispensed through repeated cycles of 10 μL aliquots, the error will scale with the square root of the number of dispenses required to obtain the target volume.

**Fig. 62:**
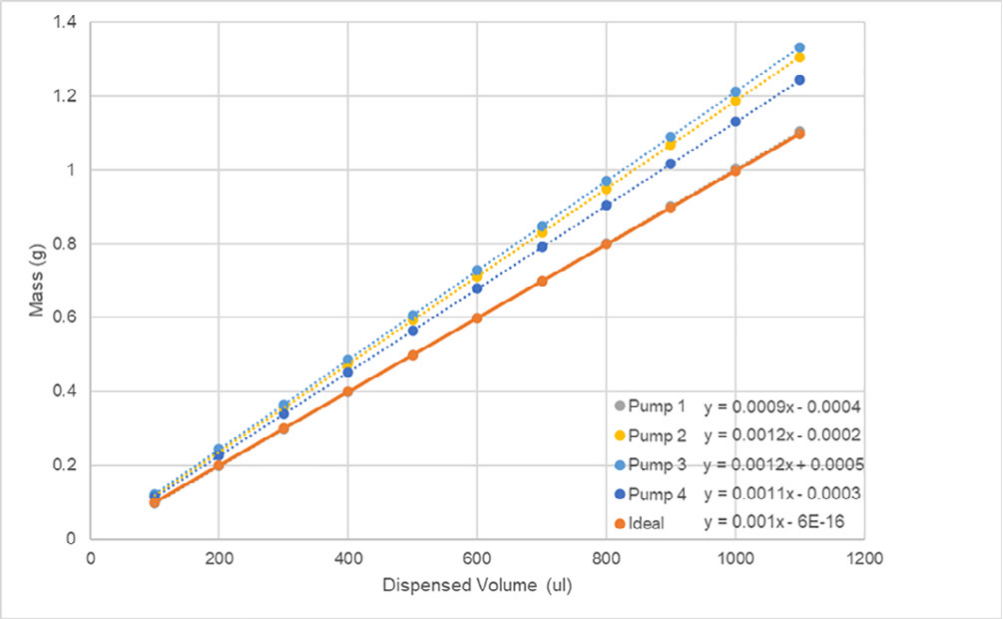
Pumps 1–4 dispensed water mass in a series of 100 μL aliquots.

The manufacturer specified pump tolerance is +/- 15%. The four pumps used in one Sidekick were found to deliver between 9.5 and 12.0 μL (Table 1), determined by measuring the mass of 100 μL aliquots of water dispensed (Fig. 62). Since the precision of the pumps is very high (0.1 μL), the pump tolerance can be accounted for through calibration, if desired.

**Table 1:** Pumps 1–4 standard deviation, and average dispense volume per 10 μL aliquot.

	Average Volume (μL) per Dispense Cycle
Ideal	10.0
Pump 1	9.5 ± 0.1
Pump 2	11.9 ± 0.1
Pump 3	12.0 ± 0.1
Pump 4	11.2 ± 0.1

The second Sidekick’s volume precision and accuracy were evaluated by dispensing between 80 and 350 μL of water into pre-weighed vials. The predicted sample mass was calculated from the density of water at ambient (21.4 °C) temperature and compared to the actual mass dispensed. Five volumes were selected within the aforementioned range and three replicates of each volume were obtained. Mass was measured using a four-decimal place balance with an estimated uncertainty of ± 0.2 mg. The relative error for three replicates ranged from 0.6 to 2.1 %, with the largest error observed for the smallest volume dispensed. For all volumes evaluated, the amount of water dispensed was approximately 11% greater than the desired volume. Given that the tolerance of the pumps reported by the manufacturer is 15%, this deviation is unexpected. As in the case of the first Sidekick, the systematic error could be accounted for, should increased accuracy be desired.

**Fig. 63:**
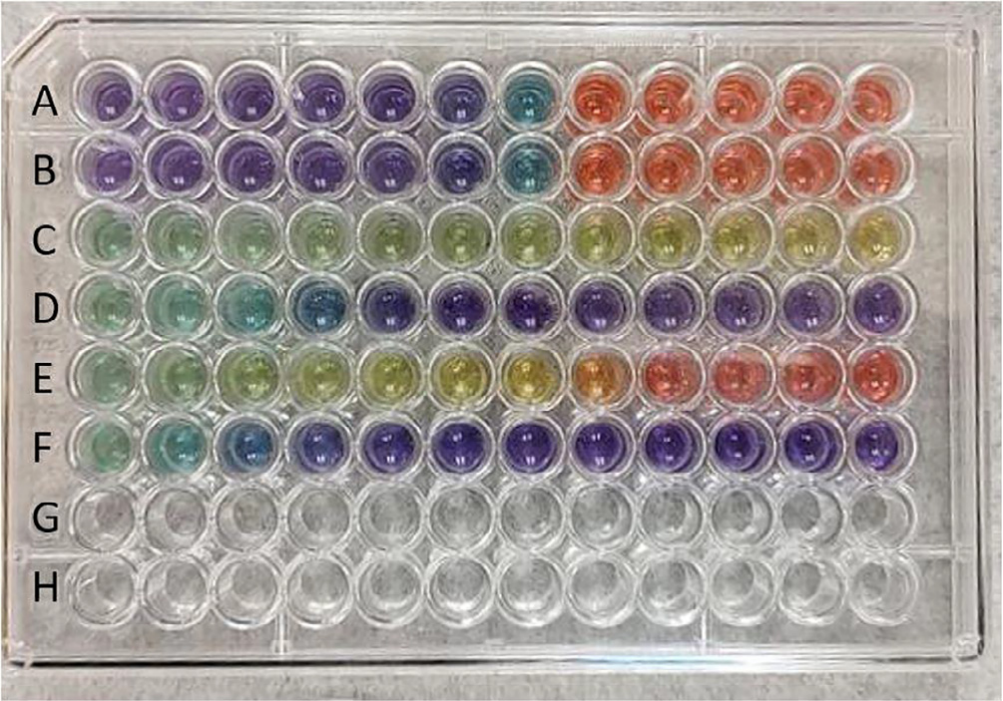
Well plates filled with varying amounts of 0.1 M hydrochloric acid, 0.1 M sodium hydroxide and 0.1 M phosphate buffer (pH 7.6). Rows A and B: Total volume kept constant (120 μL) with acid increasing from 0 to 110 μL. Row C: 100 μL phosphate buffer with increasing amounts of acid from 0 to 120 μL. Row D: 100 μL phosphate buffer with increasing amounts of base from 0 to 120 μL. Rows E and F: as rows C and E but with 50 μL phosphate buffer. All wells received 10 μL universal indicator solution.

One example application of the Sidekick is in exploring acid/base chemistry, a topic commonly covered in an introductory Analytical Chemistry course. Typically, students explore the capacity of buffers to resist pH changes by performing titrations. The Sidekick enables a colorimetric approach to the same concept. In Fig. 63 a 96 well plate is filled with varying mole fractions of sodium hydroxide and hydrochloric acid (Rows A and B). Addition of universal indicator clearly shows the acidic (red) and basic (purple) conditions as well as the sharp transition through a neutral (blue) solution. Rows C and D show the influence of a buffer, in this case a 0.1 M phosphate buffer with a pH of 7.6. Addition of acid (row C) to the buffer shows a slight decrease in pH (blue to yellow). Addition of base (row D) shows a more prominent increase to basic conditions, which is expected since the buffer was prepared with a smaller fraction of conjugate acid than base. When using a smaller amount of buffer (rows E and F) one observes that the buffer is unable to maintain a constant pH with addition of either strong acid or strong base. Automating the mixing of 72 solutions in this case provides a facile approach to visualizing an important chemical concept.

## Conclusion

We presented the design of an open-source, low-cost, small, and easily constructed automated liquid dispenser. An unusual kinematics approach allows for the use of primarily 3D-printed parts, reducing the cost of the mechanical assembly to approximately $151 USD. This design also has the benefit of providing a small operating footprint, which enables our device to be easily stored or used inside space-constrained environments such as gloveboxes and anaerobic chambers. Using digital dispensing pumps minimizes the need for calibration. The system is controlled over a serial port, either by a set of simplified serial commands or using a subset of G-Code commands.

## Declaration of Competing Interest

The authors declare that they have no known competing financial interests or personal relationships that could have appeared to influence the work reported in this paper.
